# Genetic alterations of the SUMO isopeptidase SENP6 drive lymphomagenesis and genetic instability in diffuse large B-cell lymphoma

**DOI:** 10.1038/s41467-021-27704-8

**Published:** 2022-01-12

**Authors:** Markus Schick, Le Zhang, Sabine Maurer, Hans Carlo Maurer, Konstandina Isaakaidis, Lara Schneider, Upayan Patra, Kathrin Schunck, Elena Rohleder, Julia Hofstetter, Apoorva Baluapuri, Anna Katharina Scherger, Julia Slotta-Huspenina, Franziska Hettler, Julia Weber, Thomas Engleitner, Roman Maresch, Jolanta Slawska, Richard Lewis, Rouzanna Istvanffy, Stefan Habringer, Katja Steiger, Armin Baiker, Robert A. J. Oostendorp, Cornelius Miething, Hans-Peter Lenhof, Florian Bassermann, Björn Chapuy, Matthias Wirth, Elmar Wolf, Roland Rad, Stefan Müller, Ulrich Keller

**Affiliations:** 1 Department of Hematology, Oncology and Cancer Immunology, Campus Benjamin Franklin, Charité - Universitätsmedizin Berlin, corporate member of Freie Universität Berlin and Humboldt-Universität zu Berlin, 12203 Berlin, Germany; 2 Internal Medicine III, School of Medicine, Technische Universität München, 81675 Munich, Germany; 3 Internal Medicine II, School of Medicine, Technische Universität München, 81675 Munich, Germany; 4 Center for Bioinformatics, Saarland Informatics Campus, Saarland University, 66123 Saarbrücken, Germany; 5 Institute of Biochemistry II, Goethe University, Medical School, 60590 Frankfurt, Germany; 6 Cancer Systems Biology Group, Theodor Boveri Institute, University of Würzburg, 97074 Würzburg, Germany; 7 Institute of Pathology, School of Medicine, Technische Universität München, 81675 Munich, Germany; 8 Institute of Molecular Oncology and Functional Genomics, TUM School of Medicine, Technische Universität München, 81675 Munich, Germany; 9 Center for Translational Cancer Research (TranslaTUM), Technische Universität München, 81675 Munich, Germany; 10 Bavarian Health and Food Safety Authority, 85764 Oberschleißheim, Germany; 11 Department of Hematology, Oncology, Stem Cell Transplantation, University Medical Center Freiburg, 79106 Freiburg, Germany; 12 Department of Hematology and Oncology, University Medical Center Göttingen, 37075 Göttingen, Germany; 13 German Cancer Consortium (DKTK), German Cancer Research Center (DKFZ), 69120 Heidelberg, Germany; 14 Max-Delbrück-Center for Molecular Medicine, 13125 Berlin, Germany

**Keywords:** B-cell lymphoma, Tumour biomarkers, Sumoylation

## Abstract

SUMOylation is a post-translational modification of proteins that regulates these proteins’ localization, turnover or function. Aberrant SUMOylation is frequently found in cancers but its origin remains elusive. Using a genome-wide transposon mutagenesis screen in a MYC-driven B-cell lymphoma model, we here identify the SUMO isopeptidase (or deconjugase) SENP6 as a tumor suppressor that links unrestricted SUMOylation to tumor development and progression. Notably, *SENP6* is recurrently deleted in human lymphomas and SENP6 deficiency results in unrestricted SUMOylation. Mechanistically, SENP6 loss triggers release of DNA repair- and genome maintenance-associated protein complexes from chromatin thereby impairing DNA repair in response to DNA damages and ultimately promoting genomic instability. In line with this hypothesis, SENP6 deficiency drives synthetic lethality to Poly-ADP-Ribose-Polymerase (PARP) inhibition. Together, our results link *SENP6* loss to defective genome maintenance and reveal the potential therapeutic application of PARP inhibitors in B-cell lymphoma.

## Abstract (web summary)

SUMOylation is a post-translational modification that has been shown to be altered in cancer. Here, the authors show that loss of the SUMO isopeptidase SENP6 leads to unrestricted SUMOylation and genomic instability promoting lymphomagenesis and generating vulnerability to PARP inhibition.

## Introduction

Covalent ligation of small ubiquitin-like modifiers SUMO1, SUMO2, or SUMO3 to a target protein (SUMOylation) is an important post-translational modification that regulates the localization, stability, and activity of these target proteins. As such, SUMOylation serves as an essential regulatory mechanism for fundamental cellular processes such as cell cycle progression, DNA damage repair, nucleocytoplasmic transport, transcription, and chromatin remodeling^1,2^.

Whereas SUMO1 is typically conjugated as a monomer or a chain terminator, SUMO2 and SUMO3 are prone to form SUMO chains via internal lysine residues^3–5^. SUMO modification typically facilitates transient protein–protein interactions and SUMO chains provide a binding interface for a specific subtype of ubiquitin ligases, known as SUMO-targeted ligases (StUbL). RNF4, the best characterized StUbL in humans, catalyzes proteolytic- or non-proteolytic ubiquitylation of polySUMOylated targets^4^. SUMOylation is a dynamic and fully reversible process. Deconjugation of SUMOs from substrates is primarily catalyzed by sentrin/SUMO-specific proteases (SENPs), which comprises six members (SENP1, SENP2, SENP3, SENP5, SENP6, and SENP7) in human cells. Whereas SENP1, SENP2, SENP3, and SENP5 display activities on both C-terminal SUMO maturation, which is a prerequisite for conjugation, and deconjugation, SENP6 and SENP7 specifically edit polymeric SUMO chains^3^. Generally, the balanced SUMO conjugation–deconjugation orchestrates the plasticity of SUMO-dependent protein–protein interactions. Therefore, the activities of SENPs are critical determinants of cellular SUMO homeostasis and SUMO signaling.

Importantly, activation of oncogenes, such as *MYC*, or mutant *KRAS* or *NOTCH*, results in the upregulation of cellular SUMOylation, pointing to a role of enhanced SUMOylation in tumor formation or progression. This is best established in cellular models of MYC-driven cancers, where loss-of-function screens revealed a cancer-specific dependency on SUMOylation^6,7^. However, the underlying mechanisms for enhanced SUMOylation are not well understood. Furthermore, it has remained elusive if deregulated SUMOylation causally contributes to cancer pathogenesis.

Diffuse large B-cell lymphoma (DLBCL) is the most common aggressive B-cell lymphoma (BCL) and a clinically and molecularly heterogeneous disease with a complex genetic background^8,9^. Recent large-scale genomic studies highlighted multiple potential driver alterations that co-occur and underscore the central role of MYC in BCL pathogenesis^9–11^. Due to the large number of genes altered by genetic and non-genetic mechanisms, it remains challenging to pinpoint functionally relevant drivers of B-cell lymphomagenesis. Therefore, these genomic studies need to be complemented by unbiased functional in vivo screens. The recently described in vivo *piggyBac* (PB) transposon mutagenesis system has evolved as a powerful tool to dissect cancer pathogenesis^12–14^. An unbiased selection for biologically relevant events and the combination with OMICs data from human cancers allows to assess the quality of alterations and to filter relevant from non-relevant genetic and non-genetic alterations. Therefore, we here performed a genome-wide in vivo transposon mutagenesis screening in a model of aggressive BCL and link *SENP6* loss to defective genome maintenance and synthetic lethality to PARP inhibition.

## Results

### Transposon mutagenesis accelerates MYC-driven lymphomagenesis

**Fig. 1:**
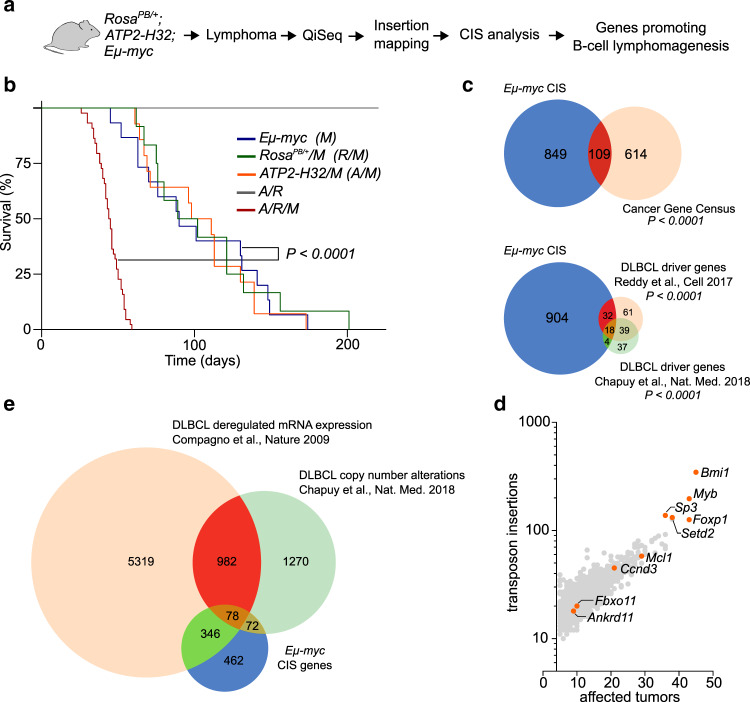
Transposon mutagenesis promotes B-cell lymphomagenesis. **a** Outline of experimental setup for the identification of genetic alterations that promote MYC-mediated B-cell lymphomagenesis. **b** Kaplan–Meier survival curves of the indicated cohorts of mice. In total 90 mice were aged up to 220 days to investigate the effects of *piggyBac* transposon mutagenesis on lymphomagenesis on an MYC-activated background. *Eµ-myc* (M), *n* = 15; *Rosa*^*PB/+*^*/M* (R/M), *n* = 12; *ATP2-H32/M* (A/M), *n* = 14; A/R, *n* = 5; A/R/M, *n* = 44. The mean survival times (days) were 44.5 for the A/R/M cohort and 90 for the M cohort. *P* < 0.0001. *P*-value determined by log-rank (Mantel–Cox) test. **c** Venn diagram showing the overlap between common insertion sites (CISs) in A/R/M lymphomas and genes listed in the Cancer Gene Census or B cell lymphoma (DLBCL) driver genes described in refs. ^9,10^. *P*-value determined by Fisher’s exact test. **d** Graph showing the number of transposon insertions per CIS against the number of affected tumors. Every dot represents one CIS. The information on the number of transposon insertions and the number of affected tumors are provided in Supplementary Data 1. Selected examples listed in the Cancer Gene Census database are highlighted in orange. **e** Venn diagram showing the overlap between CIS genes derived from the *Eµ-myc* transposon mutagenesis screen and genes with differential mRNA expression (FDR *P*-value < 0.05) in DLBCL samples when compared to healthy GC B-cells^21^, or genes affected by copy number alterations in DLBCL cases^10^.

Over 70% of all human cancers show elevated MYC levels and B-cell specific MYC expression in mice initiates BCL with full penetrance. However, to fully transform B-cells, alterations of other cancer genes are required^15,16^. To identify these functional co-dependencies of MYC-driven B-cell lymphomagenesis, we performed a genome-wide forward-genetic in vivo screen using the *PB* mutagenesis system^12^. To achieve mutagenesis in MYC-driven lymphoma, *Eµ-myc (M)* mice were sequentially crossed to mice expressing the *piggyBac* transposase *(R)* and to transgenic mice carrying the *ATP-H32* transposon *(A)*, the latter being mobilized by the transposase^12^. A total of 48 *ATP2-H32;Rosa26;*^*PB/+*^*Eµ-myc (A/R/M)* triple-transgenic mice were generated and further analyzed (Fig. 1a). PB mutagenesis led to accelerated lymphomagenesis and significantly reduced animal survival (median survival of 44.5 days and 90 days in *A/R/M* mice and *M* mice, respectively) (Fig. 1b). Extensive phenotype analysis of lymphomas from *A/R/M* mice revealed the expected lymphoma characteristics comparable to those observed in *Eµ-myc* control mice (Supplementary Fig. 1). Thus, PB mutagenesis significantly accelerated BCL onset.

To identify the genetic alterations responsible for accelerated B-cell lymphomagenesis, we performed quantitative insertion-site sequencing (QiSeq) of the murine lymphomas and subsequent bioinformatics analysis with established pipelines^17^ (Fig. 1a). Analyzing 48 lymphomas, we identified 126,770 non-redundant transposon insertion sites in total. Next, we identified genomic regions that harbored more transposon insertions than expected by chance by applying Gaussian Kernel convolution analysis^17^ and identified 958 common insertion sites (CISs) (Supplementary Data 1). Importantly, we observed only a minor overlap to previously described transposon screenings investigating B-cell lymphomagenesis^17,18^, which we attribute to the different and characteristic genetic backgrounds of each screening (Supplementary Fig. 2).

To interrogate the potential biological and clinical importance of these CISs, we analyzed their enrichment in genes known to be involved in human cancers. We found that these 958 CISs were significantly enriched (*P* < 0.0001) among genes listed in the Cancer Gene Census^19^ (Fig. 1c, upper panel and Supplementary Data 2) and that many well-known cancer genes were identified (Fig. 1d). Importantly, relating to the chosen experimental lymphoma model, CISs were also significantly enriched in the Reddy et al.^9^ set of 150 DLBCL driver genes (*P* < 0.0001) and in the Chapuy et al.^10^ set of 98 DLBCL driver genes (*P* < 0.0001) (Fig. 1c, lower panel and Supplementary Data 2). Notably, 52% of the CISs identified in *A/R/M* mice were deregulated in human BCL, by either gene expression or copy number alteration (Fig. 1e and Supplementary Data 2), indicating the potential relevance of these CIS in human BCL. Thus, our PB screen defined a large catalog of genes that potentially promote B-cell lymphomagenesis in murine and human lymphoma.

### SENP6 is a tumor suppressor of B-cell lymphomagenesis

**Fig. 2:**
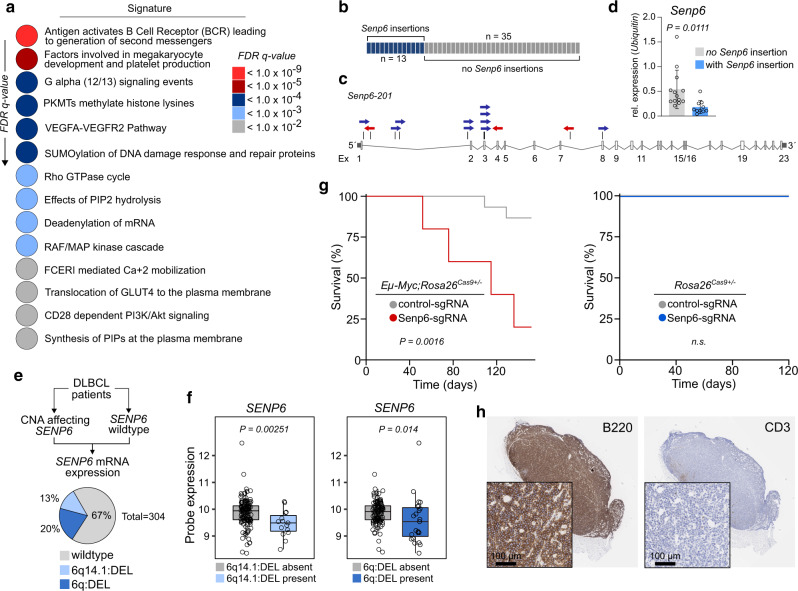
*SENP6* is a tumor suppressor of B-cell lymphomagenesis and is recurrently deleted in human DLBCL. **a** *Eµ-myc* CISs were analyzed using the Reactome database. Color-coded *FDR q-value* is shown for the top fourteen pathways. **b** Schematic depiction of *A/R/M* lymphomas with *Senp6* transposon insertion and without *Senp6* transposon insertion. **c** Transposon insertion pattern in *Senp6* indicates tumor suppressor function and the number of affected tumors. Only the dominant insertion per tumor is shown. **d** *Senp6* expression in *A/R/M* lymphomas with *Senp6* transposon insertions (*n* = 10) and *A/R/M* lymphomas without *Senp6* transposon insertion (*n* = 13). *Senp6* expression was normalized to *Ubiquitin*. Data are presented as mean ± SD. *P*-value determined by unpaired *t*-test (two-tailed). **e** Copy number alterations (CNA) affecting *SENP6* in human DLBCL (*n* = 304). *Wildtype*, *n* = 205; 6q14.1:DEL, *n* = 38; 6q:DEL, *n* = 61. **f** *SENP6* mRNA levels in DLBCL groups according to their *SENP6* copy number status. 6q14.1:DEL absent, *n* = 122; 6q14.1:DEL present, *n* = 15; 6q:DEL absent, *n* = 114; 6q:DEL present, *n* = 23. The centerline of the box plot is the median. The box extends from the 25th to 75th percentiles. Whisker length is from minimum to maximum. *P-value* determined by Wilcoxon Rank Sum Test (two-sided). **g** Left panel: Kaplan–Meier survival curves of mice transplanted with *Eµ-myc;Rosa26*^*Cas9*^ HSPCs transduced with sgRNAs targeting *Senp6*. *Senp6*, *n* = 5; control *n* = 15; *p* = 0.0016. *P-value* determined by log-rank (Mantel–Cox) test. Right panel: Kaplan–Meier survival curves of mice transplanted with *Rosa26*^*Cas9*^ HSPCs transduced with sgRNAs targeting *Senp6*. *Senp6*, *n* = 6; control *n* = 5. **h** Representative histological and immunohistochemical analysis of one of three analyzed *Eµ-myc;Rosa26*;^*Cas9*^Senp6-sgRNA HSPC-derived lymphomas for B220 and CD3 expression. The analysis was repeated three times for each cohort with similar results.

The in vivo *PB* screening approach allows positive selection of driver alterations for B-cell lymphomagenesis. Hypothesizing that several of the identified CISs converge in common pathways during B-cell lymphomagenesis, we performed pathway enrichment analysis using the GeneTrail2 1.6 web service^20^ and the Reactome database. Among the 41 significantly enriched pathways were several pathways with well-defined roles in cancer and lymphomagenesis, including the *“*VEGFA-VEGFR2*”* pathway and the “Antigen activates B-cell Receptor (BCR) leading to generation of second messengers” pathway (Fig. 2a; full list of enriched pathways, Supplementary Data 3). Notably, “SUMOylation of DNA damage response (DDR) proteins” (*P* = 7.81 × 10^−5^) scored among the top altered pathways (Fig. 2a) prompting us to hypothesize that proteins promoting B-cell lymphomagenesis, and more generally cancer pathogenesis, are part of the DDR network and are preferentially deregulated by the SUMO modification system. This hypothesis is supported by the frequent finding that aberrant SUMOylation is linked to a particularly robust cancer phenotype, treatment resistance, and poor prognosis^1^. To investigate which specific genes in the positive or negative regulatory SUMOylation pathway were integration sites within the transposon screen, we searched the CISs identified in the *A/R/M* screen and identified the SUMO protease gene *Senp6* as a putative cancer driver gene in 13 out of 48 *A/R/M* lymphomas (Fig. 2b and Supplementary Data 1). SENP6 belongs to the family of SUMO deconjugating cysteine proteases and preferentially acts by dismantling SUMO chains^3^. The transposon insertion pattern of *Senp6* was characterized by scattered and bi-directional insertions, suggesting *Senp6* as a tumor suppressor gene (Fig. 2c). In line with the suggested function as a tumor suppressor, *Senp6* mRNA expression was significantly lower *in A/R/M* lymphomas with transposon insertions in *Senp6* (Fig. 2d). To assess the relevance of this finding for human B-cell lymphomagenesis, we queried representative human BCL datasets for *SENP6* alterations. While we found only infrequent somatic mutations in *SENP6* (1% in Chapuy et al.^10^), we found recurrent focal deletions of 6q14.1/*SENP6* and recurrent arm level deletions of 6q/*SENP6* in DLBCL with frequencies of 13% (38/304) and 20% (61/304) respectively (Fig. 2e)^10^. Importantly, focal 6q14.1/*SENP6* loss and arm level 6q/*SENP6* loss had significantly reduced abundance of *SENP6* transcripts (Fig. 2f and Supplementary Fig. 3a). Moreover, we also found *SENP6* deletions with a frequency of 29% (14/48) in a second DLBCL dataset (Supplementary Fig. 3b, TCGA DLBCL dataset), underscoring the relevance of the screening result for human lymphomas. To stress the association of *SENP6* loss and MYC, we performed gene set enrichment analysis (GSEA) and found that gene sets showing activated MYC signaling were significantly enriched in the subgroup of DLBCL cases harboring *SENP6* deletion (Supplementary Figs. 3c and 4a, b). However, the SENP6 status did not directly affect MYC protein expression nor MYC binding as determined by immunoblot and ChIP-sequencing analysis (Supplementary Fig. 5a–d). Further, SENP6 mRNA expression was not affected in DLBCLs harboring MYC alterations (Supplementary Fig. 4c).

We also identified *SENP6* deletions in a broad range of BCL sub-entities, including marginal zone (4.2%), mantle cell (7.7%), follicular (15.6%), and Burkitt lymphoma (6.5%), indicating a possible role of SENP6 or the SUMOylation pathway in other lymphomas (Supplementary Fig. 3d). Finally, to prove a functional role for SENP6 loss in an established model of B-cell lymphomagenesis in vivo, we generated hematopoietic stem cell grafts from *Eµ-myc;Rosa26*^*Cas9*^ mice. E13.5 fetal liver-derived hematopoietic stem and progenitor cells (FL-HSPC) were transduced with lentivirus encoding a sgRNA targeting *Senp6* (Supplementary Fig. 3e). Transduction efficiency was between 20 and 30% as assessed by the GFP reporter. Syngeneic wild-type mice receiving *Senp6* sgRNA FL-HSPC grafts were monitored for lymphoma onset. Loss of *Senp6* significantly promoted B-cell lymphomagenesis in vivo (Fig. 2g, left panel and Fig. 2h), validating the findings from the PB screen for this specific gene. As expected, we detected insertions and deletions (InDels) with associated loss of SENP6 protein in *Senp6-*sgRNA lymphomas (Supplementary Fig. 3f, g). Importantly, depletion of SENP6 did not promote tumorigenesis in *Rosa26*^*Cas9*^ control FL-HSPCs recipients (Fig. 2g, right panel), indicating that a co-driver such as MYC is needed for SENP6 loss to promote tumorigenesis. Thus, this in vivo experiment proved that loss of *Senp6* accelerated MYC-driven B-cell lymphomagenesis in mice, providing direct experimental evidence that deregulated SUMO deconjugation accelerates cancer formation.

**Fig. 3:**
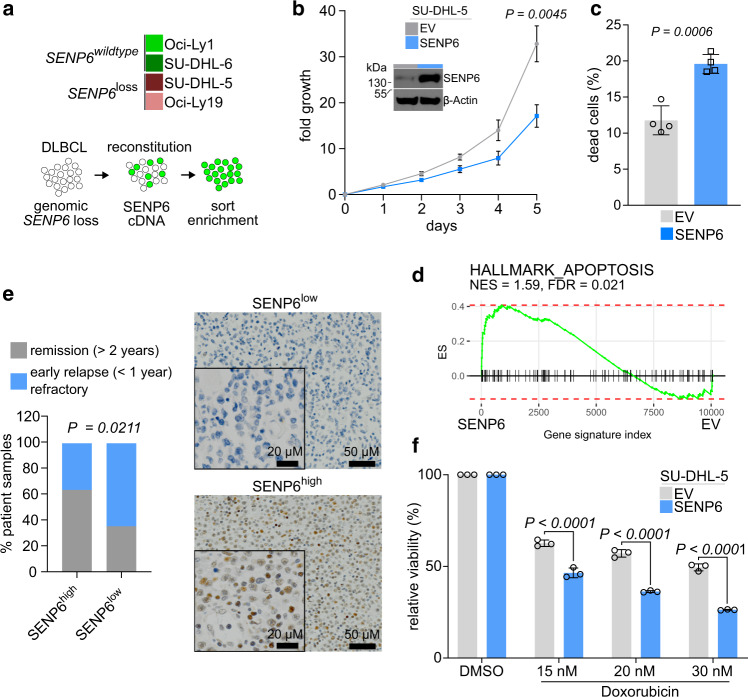
Low SENP6 expression is associated with aggressive tumor biology. **a** Results of *SENP6* copy number analysis of human DLBCL cell lines derived from cancer cell line encyclopedia (top) and experimental workflow for SENP6 reconstitution (bottom). **b** Analysis of SENP6 protein expression and cell proliferation upon SENP6 reconstitution versus empty vector (EV) transduced control cells (*n* = 3 independent experiments). Data are presented as mean ± SD. *P*-value determined by unpaired *t*-test (two-tailed). **c** Flow cytometry analysis of cell death of the cell lines described in **b** using propidium iodide staining (*n* = 4 independent experiments). Data are presented as mean ± SD. *P*-value determined by unpaired *t*-test (two-tailed). **d** GSEA of expression data derived from transcriptome profiling of the cell lines described in **b** with the indicated gene set. **e** Representative immunohistochemical stainings of human DLBCL cases with SENP6 low (score 0) and high SENP6 expression (score 3), magnification ×200, insert ×400. SENP6 analysis revealed an association between SENP6 expression levels (high versus low) and clinical outcome (remission > 2 years, *n* = 37 versus early relapse/refractory <1 year, *n* = 38). *P*-value determined by Fisher’s exact test (two-sided). **f** Viability of indicated cell lines after 48 h treatment with the indicated concentrations of doxorubicin relative to control. Viability is determined by propidium iodide staining and flow cytometry measurement. *P*-value determined by two-way ANOVA; Bonferroni’s multiple comparisons test. Data are presented as mean ± SD from three independent experiments.

To gain insight into the associated mechanisms, we searched the Cancer Cell Line Encyclopedia (https://portals.broadinstitute.org/ccle) and identified DLBCL cell lines with (SU-DHL-5, OCI-Ly19) and without (SU-DHL-6, OCI-Ly1) genomic *SENP6* loss (Fig. 3a, upper panel). Next, we reconstituted SENP6 expression in *SENP6*-deleted SU-DHL-5 cells and analyzed the cellular consequences of reduced and reconstituted SENP6 levels (Fig. 3a, lower panel, Fig. 3b inset). Reconstituted SENP6 expression in SU-DHL-5 cells significantly reduced cell growth (Figs. 3b and S3h), which was associated with increased cell death (Fig. 3c). Moreover, transcriptome profiling and subsequent GSEA indicated enriched expression of genes associated with apoptosis (Fig. 3d).

To investigate the association of SENP6 expression with the aggressiveness of human BCL, we generated tissue microarrays (TMAs) derived from 75 DLBCL patients. Nuclear SENP6 expression was evaluated on TMAs by immunohistochemistry. Based on the percentage of positive tumor cells patients were divided into groups of SENP6^high^ (*N* = 36) vs. SENP6^low^ (*N* = 39). Tumors from DLBCL patients with primary refractory disease or early relapse were enriched in the SENP6^low^ *group* (*P* = 0.0211, Fig. 3e), indicating that low SENP6 levels were associated with inferior prognosis. To demonstrate an adverse association of SENP6 expression and susceptibility to doxorubicin (DRB), we showed that SU-DHL-5 DLBCL cells exhibited increased cell death upon DRB treatment after reconstitution of SENP6 expression (Figs. 3f and S3i).

Taken together, these data identify SENP6 as a functionally relevant tumor suppressor in murine and human BCL. Moreover, low SENP6 expression is associated with adverse prognosis in DLBCL patients.

### SENP6 is a critical determinant for SUMO homeostasis in BCL

**Fig. 4:**
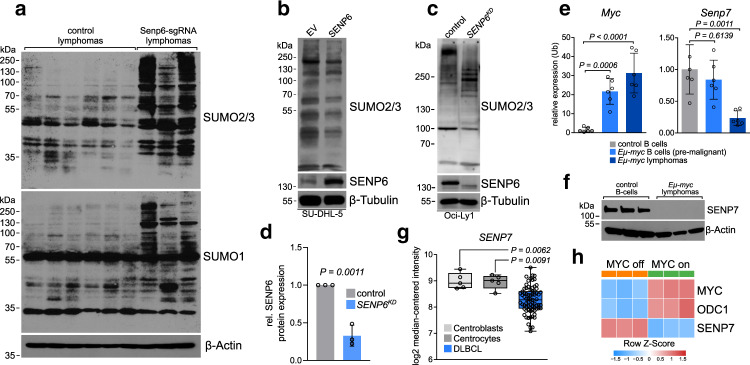
Loss of SENP6 promotes unrestricted SUMOylation in B-cell lymphoma. **a** Immunoblot analysis of overall SUMOylation using SUMO1 and SUMO2/3 antibodies. *Eµ-myc* control lymphomas (*n* = 6) are compared to *Senp6-sgRNA* lymphomas (*n* = 3) derived from in vivo validation experiments (Fig. 2g, left panel). **b** Immunoblot analysis of human SU-DHL-5 cells after reconstitution of SENP6 is described in Fig. 3b to investigate the overall status of SUMO2/3-conjugated proteins. EV empty vector control. **c** Immunoblot analysis of human OCI-Ly1 DLBCL cells after CRISPR/Cas9-mediated depletion of *SENP6* to investigate the overall status of SUMO2/3-conjugated proteins. SUMO chains induced by SENP6 depletion are indicated. **d** Quantification of the SENP6 western blots in OCI-Ly1 control and *SENP6*^*KD*^ cells. Protein expression of SENP6 in control cells was arbitrarily set to 1. Each dot represents a biological replicate (Data are presented as mean ± SD from *n* = 3 independent experiments). *P*-value determined by unpaired *t*-test (two-tailed). **e** *Senp7* and *Myc* expression in CD19^+^ B-cells derived from wild type mice (*n* = 6), CD19^+^ B-cells from premalignant *Eµ-myc* mice (*n* = 6) and *Eµ-myc* lymphomas (*n* = 6). *Senp7* and *Myc* expression was normalized to *Ubiquitin*. Data are presented as mean ± SD. *P*-value determined by one-way ANOVA; Tukey’s post hoc test. **f** Immunoblot analysis of the indicated proteins comparing splenic CD19^+^ control B-cells (*n* = 3) and *Eµ-myc* lymphomas (*n* = 3). **g** *SENP7* expression in control B-cell (centroblasts, *n* = 5; centrocytes, *n* = 5) and in primary DLBCL samples (*n* = 73) in the GSE12195 dataset. The centerline of the box plot is the median. The box extends from the 25th to 75th percentiles. Whisker length is from minimum to maximum. *P*-value was determined by one-way ANOVA; Tukey’s post hoc test. **h** *MYC, ODC1*, and *SENP7* expression after the repression of MYC for 24 h in the human P493-6 cell line carrying a tet-repressible *MYC* transgene in the GSE32219 dataset.

To test whether SENP6 is crucial for regulating the SUMO state in MYC-driven lymphoma, we performed immunoblot analysis of SENP6-depleted lymphomas derived from the in vivo validation experiments described above (Fig. 2g). The high level of SUMOylated proteins in *Eµ-myc* lymphomas was further enhanced upon deletion of *Senp6* (Fig. 4a). The effect was more pronounced on SUMO2/3 than SUMO1 conjugates underscoring the preference of SENP6 for SUMO2/3 (Fig. 4a). Notably, loss of SENP6 triggers the accumulation of high molecular weight SUMO2/3 conjugates indicative of compromised polySUMO chain editing. To investigate whether reconstitution of SENP6 is sufficient to effectively control the level of global protein SUMOylation in human BCL, we analyzed the level of SUMO2/3 conjugated proteins in the SU-DHL-5 DLBCL cell line after reconstitution of SENP6 expression and found a strong reduction of SUMO2/3 conjugates (Fig. 4b). Accordingly, we found accumulation of SUMO2/3 conjugates following CRISPR/Cas9-mediated depletion of SENP6 in human OCI-Ly1 cells (OCI-Ly1 *SENP6*^*KD*^) (Fig. 4c, d). Considering that the SENP6-related isopeptidase SENP7 also primarily functions in trimming SUMO chains we expected that SENP7 might, at least partly, rescue the effects of SENP6 depletion on SUMO deconjugation. To delineate a potential interplay of both isopeptidases in tumor suppression, we first investigated the *Senp7* expression level during murine B-cell lymphomagenesis in the *Eµ-myc* model. Notably, *Senp7* transcript level was suppressed during lymphomagenesis and SENP7 protein was absent in *Eµ-myc* lymphomas (Fig. 4e, f). To confirm this result in human BCL, we analyzed a gene expression data set comparing human DLBCL samples to control B-cells derived from germinal center (GC) (GSE12195)^21^. Despite the presence of *SENP7* amplifications (Supplementary Fig. 6a, b), the *SENP7* transcript level was reduced in human DLBCL (Fig. 4g). Also, as suggested by the broad range of mRNA expression, we observed a broad range of nuclear SENP7 protein expression in human DLBCL tissue samples (Supplementary Fig. 6c, d). The broad spectrum of *SENP7* expression levels in human DLBCL samples suggested that *SENP7* might be suppressed by various genetic lesions. We analyzed the effects of *MYC*, the primary genetic lesion in our screen, on *SENP7* expression in a dataset of the human P493-6 lymphoma cell line (GSE32219)^22^ carrying a tetracycline-repressible *MYC* transgene. *SENP7* expression was rapidly up-regulated upon repression of *MYC* (Fig. 4h).

In summary, our data show that loss of SENP6 leads to unrestricted SUMOylation in BCL. Moreover, our results suggest that the suppression or inactivation of the related SENP7 contributes to the hyperSUMOylation phenotype in MYC-driven lymphomas.

### SENP6 is induced by MYC and required for proper DNA damage checkpoint activation

**Fig. 5:**
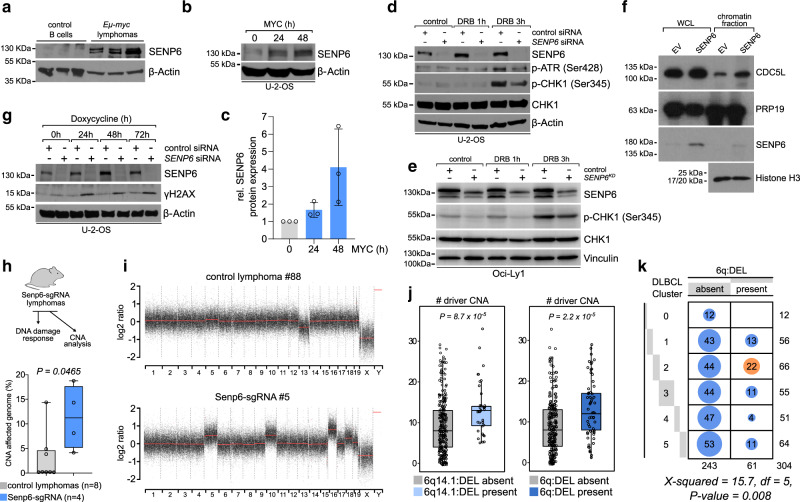
SENP6 is activated by MYC and controls DNA damage checkpoint activation. **a** Immunoblot analysis of the indicated proteins in splenic CD19^+^ B-cells (*n* = 3) purified from wild-type mice and in *Eµ-myc* lymphomas (*n* = 3). **b** Immunoblot analysis of indicated proteins in U-2-OS cells after induction of MYC for the indicated times. **c** Quantification of the SENP6 western blots in U-2-OS cells after induction of MYC for the indicated times. Protein expression of SENP6 at 0 h was arbitrarily set to 1. Data are presented as mean ± SD from *n* = 3 independent experiments. **d** Immunoblot analysis of indicated proteins in U-2-OS cells after transfection with specific SENP6 siRNA or control siRNA and doxorubicin (DRB) treatment for the indicated times. The experiment was repeated three times with similar results. **e** Immunoblot analysis of indicated proteins in OCI-Ly1 human DLBCL cell line described in Fig. 4c after doxorubicin (DRB) treatment for the indicated times. The experiment was repeated three times with similar results. **f** Immunoblot analysis of whole-cell lysate (WCL) and chromatin fraction of SU-DHL-5 EV and SENP6 cells with the indicated antibodies. Equal loading of WCL was controlled by Ponceau S staining. The experiment was repeated three times with similar results. **g** Immunoblot analysis of indicated proteins in U-2-OS cells after transfection with specific SENP6 siRNA or control siRNA and induction of MYC for the indicated times. The experiment was repeated three times with similar results. **h** Experimental setup of copy number and DNA damage analysis in *Senp6*-sgRNA lymphomas from in vivo validation experiments described in Fig. 2g left panel (upper panel). Copy number alterations (CNA) of control and *Senp6-sgRNA* lymphomas were analyzed by low coverage WGS and the fraction of CNA affected genome was compared (lower panel). X and Y chromosomes have been excluded from the analysis. The center line of the box plot is the median. The box extends from the 25th to 75th percentiles. Whisker length is from minimum to maximum. *P*-value determined by Mann–Whitney test (two-tailed). **i** Copy number plots of one *Eμ-myc* control and one *Senp6-sgRNA* lymphoma derived from in vivo validation experiments as determined by low coverage WGS. A list of all genomic alterations in *Senp6*-sgRNA lymphomas is provided in Supplementary Data 6. **j** Analysis of driver CNAs in DLBCL patients (*n* = 304). Groups were classified according to their *SENP6* copy number status. 6q14.1:DEL absent, *n* = 266; 6q14.1:DEL present, *n* = 38; 6q:DEL absent, *n* = 243; 6q:DEL present, *n* = 61. The centerline of the box plot is the median. The box extends from the 25th to 75th percentiles. Whisker length is from minimum to maximum. *P*-value determined by Wilcoxon Rank Sum Test (two-sided). **k** Enrichment of the 6q:DEL deletion affecting the SENP6 locus in the DLBCL patient cluster C2. *P*-value determined by Pearson’s Chi-squared test (one-sided).

Recent work has implicated SENP6 in DDR and checkpoint activation^23–25^. The DDR promotes checkpoint activation following DNA damage or enforced oncogene expression, which typically causes replicative stress. Activated checkpoints limit tumorigenesis by allowing DNA repair in order to maintain genomic integrity^26^. Ectopic MYC expression alone is insufficient to induce cellular transformation because it triggers checkpoint activation, cell-cycle arrest, and apoptosis through intrinsic tumor-suppressive pathways^27^. To test if SENP6 is involved in the response to MYC-induced oncogenic stress, we analyzed SENP6 protein expression in murine MYC-driven lymphomas. SENP6 protein expression was substantially elevated in *Eµ-myc* lymphomas in comparison to normal B-cells (Fig. 5a). Furthermore, in a human cell line with doxycycline-inducible MYC^28^, enforced MYC expression rapidly increased the total SENP6 protein level (Fig. 5b, c). In line with this idea, SENP6 depletion in the human OCI-Ly1 DLBCL cell line led to the enrichment of the hallmark transcriptome signature “DNA repair” indicating a high level of DNA damage following SENP6 loss (Supplementary Fig. 7). Thus, SENP6 may be involved in the response to MYC-induced oncogenic stress.

Cleavage of poly-SUMO2/3 chains by SENP6 plays a general role in the DDR^23–25^ and SENP6 has been linked to checkpoint activation. To assess the role of SENP6 in regulating DDR checkpoints, we depleted SENP6 in U-2-OS cells with a specific siRNA (Supplementary Fig. 8a) and treated the cells with DRB to promote checkpoint activation. Compared to control cells, ATR phosphorylation and phosphorylation of the downstream kinase CHK1 were reduced upon depletion of SENP6 (Figs. 5d, S8b, c). Accordingly, we observed reduced CHK1 phosphorylation in human OCI-Ly1 *SENP6*^*KD*^ cells in comparison to control cells (Figs. 5e, S8d, e).

Our previous work suggests that SENP6 affects CHK1-ATR activation by safeguarding chromatin association of the mammalian hPSO4/PRP19 complex^23^. The hPSO4/PRP19 complex, which consists of the core components PRP19, BCAS2, CDC5L, and PLRG1, facilitates recruitment of the ATR coactivator ATRIP to chromatin^29,30^. Depletion of any component compromises ATR activation as exemplified by defective CHK1 phosphorylation upon lack of CDC5L in U-2-OS cells (Supplementary Fig. 9a). To test if SENP6 controls chromatin residency of CDC5L during oncogene-induced replicative stress, we isolated chromatin from SENP6-depleted and control cells following induction of MYC expression. In this context, SENP6 depletion substantially reduced CDC5L levels in the chromatin fraction (Supplementary Fig. 9b–d). To further test whether SENP6 deficiency affects the hPSO4 complex in BCL, we performed chromatin fractionation experiments in the SENP6-deficient SUDHL5 cell line and the corresponding isogenic line, where re-expression of SENP6 restores wild-type levels. Strikingly, SENP6 loss drastically impairs chromatin occupancy of the CDC5L subunit, but not PRP19, likely contributing to the impairment of CHK1 activation (Fig. 5f).

Since proper DNA damage checkpoint activation is crucial for the maintenance of genome integrity in response to replication stress, we tested whether the cellular SENP6 status affects the genomic integrity in response to MYC-induced oncogenic stress. To this end, we depleted SENP6 and concomitantly induced MYC expression in U-2-OS cells. Cells lacking SENP6 showed a strong accumulation of DNA double-strand breaks (DSBs), indicated by γH2AX phosphorylation (Fig. 5g). Moreover, shRNA-mediated depletion of *Senp6* mRNA in murine NIH-3T3 fibroblasts induced DSBs as monitored by increased γH2AX phosphorylation and foci formation (Supplementary Fig. 8f–h) correlating with defective CHK1 phosphorylation (Supplementary Fig. 8i–k). In summary, these cell-based assays suggest that SENP6 has a critical safeguard function to maintain genome integrity during MYC-induced oncogenic stress.

To investigate if *Senp6* loss controls genome stability in vivo, we performed low coverage whole genome sequencing (WGS) on *Senp6*-deficient murine lymphomas derived from the above-mentioned in vivo validation experiments (Fig. 2g) and analyzed somatic copy number alterations (SCNAs) (Fig. 5h, upper panel). Intriguingly, in comparison to control lymphomas, Senp6-sgRNA lymphomas showed a significantly larger genome fraction with SCNAs (Fig. 5h, lower panel, Fig. 5i and Supplementary Data 6). To investigate this finding in human BCL, we investigated the effects of the *SENP6* status on genomic stability in a dataset of 304 DLBCL^10^. DLBCL were classified according to their *SENP6* copy number status and the total number of co-occurring somatic driver copy number alterations (driver CNAs) was assessed. Notably, primary DLBCL samples harboring *SENP6* loss showed a significantly higher number of SCNAs (Fig. 5j). Moreover, the 6q:DEL affecting *SENP6* was enriched in the C2 DLBCL subgroup (Fig. 5k), which is characterized by a high level of genomic instability^10^.

In summary, this suggests that SENP6 serves as a gatekeeper of genome stability in both murine and human lymphomas. Further, these data identify SENP6 as the critical deSUMOylating enzyme, which controls genome stability during oncogene-induced stress in BCL.

### SENP6 controls the SUMO/chromatin landscape in BCL

**Fig. 6:**
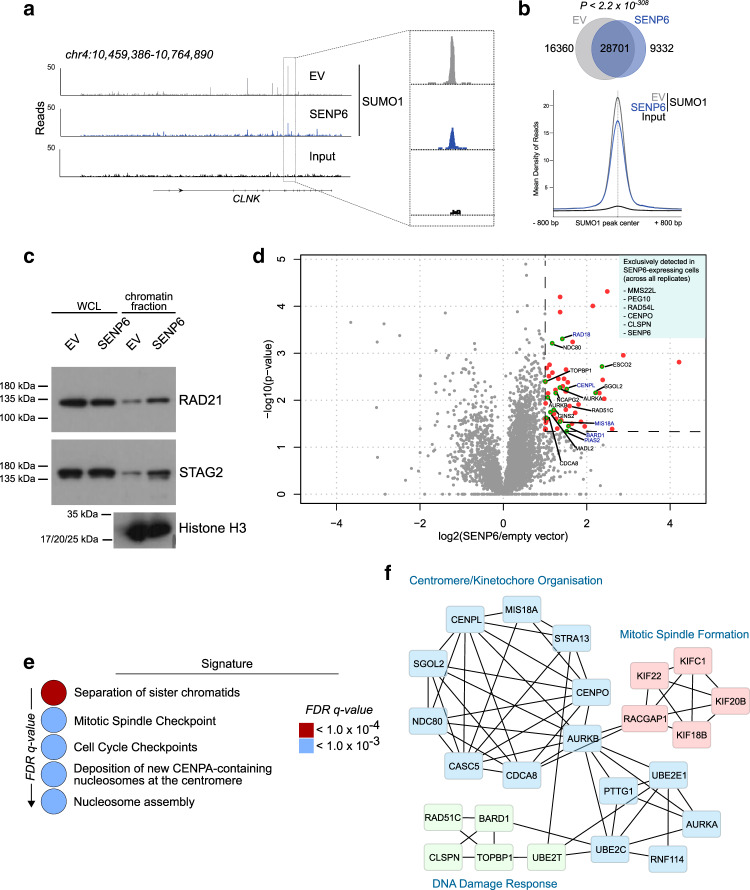
SENP6 controls the SUMO/chromatin landscape in BCL. **a** Genome browser picture of read-normalized SUMO1 ChIPseq profiles from SU-DHL-5 cells with low SENP6 expression (EV, gray) or reconstituted for SENP6 expression (SENP6, blue) described in Fig. 3b. Input is shown in black. **b** Venn diagram (top) showing overlap of SUMO1 peaks in SU-DHL-5 cells with low SENP6 expression (EV, gray) or reconstituted for SENP6 expression (SENP6, blue) described in Fig. 3b. Density plot centered at 28701 common SUMO1 peaks (lower panel). Input is shown in black. *P*-value determined by hypergeometric test for enrichment in common peaks. **c** Immunoblot analysis of whole-cell lysate (WCL) and chromatin fraction of SU-DHL-5 EV and SENP6 cells with the indicated antibodies. Equal loading of WCL was controlled by Ponceau S staining. **d** Comparative MS results of chromatin fractionation in SU-DHL-5 EV and SU-DHL-5 SENP6 cells. Volcano plot depicting proteins exhibiting significantly reduced chromatin association SENP6 EV cells (absolute log2 ratio ≥ 1 and –log10 *p* > 1.3). High-confidence hits were determined using the Student’s *t-test* comparing LFQ values of SU-DHL-5 EV and SENP6 cells (two-tailed). Most enriched targets are shown here by plotting the negative log10 *P*-value against the log2 ratio of SU-DHL-5 SENP6/SU-DHL-5 EV. Significant hits are shown by red dots, bona fide SENP6 targets are highlighted by green circles. Experiments were performed in triplicate. The inset on the right shows the six proteins that were found exclusively on chromatin in the SU-DHL-5 SENP6 cells in all three replicates. **e** Candidates from Fig. 6d were analyzed using the Reactome database. Color-coded *FDR q*-value is shown for the top five enriched pathways. **f** STRING network analysis depicting the interconnection of proteins exhibiting reduced chromatin association in SU-DHL-5 EV cells when compared with SU-DHL-5 SENP6 cells. Only connected proteins are visualized.

The above-mentioned data support the idea that SENP6 controls the SUMOylation status of chromatin-associated proteins and protein complexes^23,25^. To directly test whether alterations in SENP6 expression affect SUMOylation at chromatin, we performed ChIPseq analysis with specific antibodies against SUMO1 and SUMO2/3 in parental SU-DHL-5 DLBCL cells and the cells reconstituted with SENP6. The results revealed reduced SUMO1 (Fig. 6a) and SUMO2/3 modified proteins (Supplementary Fig. 9e) on individual genes when comparing parental cells with SENP6 re-expressing cells. Analysis of the peaks called for SUMO1 showed a substantial reduction in the number of peaks after reconstitution of SENP6 expression (Fig. 6b, upper panel). Additionally, reduced binding of SUMO1 modified proteins was found for the common peaks when SENP6 is expressed (Fig. 6b, lower panel). Similar results were noted for binding of SUMO2/3 modified proteins (Supplementary Fig. 9f), suggesting that SENP6 restricts SUMOylation of chromatin-bound proteins in BCL.

To identify critical SENP6 targets that potentially mediate its tumor-suppressive role in BCL, we scrutinized two recent proteomics studies that defined SENP6 targets (Supplementary Fig. 10a)^23,25^. Importantly, 17 of these candidate SENP6-controlled proteins were also identified as putative cancer genes in our transposon screen (Supplementary Fig. 10a, b). Pathway enrichment analysis of these common candidates using the Reactome database revealed, “SUMOylation of DDR and repair proteins” as the most highly enriched pathway underscoring the critical role of SENP6 in the control of genome stability (Supplementary Fig. 10c). Further, four out of the five other most enriched pathways are related to sister chromatin cohesion (Supplementary Fig. 10c). Intriguingly, we previously described^23^ the core components of the cohesin complex, including RAD21, STAG1, and STAG2 as bona fide SENP6 targets^23^ (Supplementary Fig. 10a), and now identified the respective genes as putative tumor suppressors in the transposon screen (Supplementary Figs. 10b and 9g). Our recent work also indicated that unrestricted SUMOylation of the cohesion complex perturbs its proper chromatin residency^23^. To explore whether the SENP6 status affects chromatin association of the cohesin complex also in BCL, we performed chromatin fractionation in parental SU-DHL-5 DLBCL cells and the corresponding cells reconstituted with SENP6. Strikingly, SENP6 deficiency strongly decreased the level of chromatin-bound STAG2 and RAD21 but did not affect their protein levels in whole-cell extracts (Fig. 6c), indicating that SENP6 deficiency in BCL impairs chromatin occupancy of cohesin core subunits. To test whether loss of cohesin occurs at distinct genomic regions we performed anti-RAD21 ChIPseq analysis in parental SU-DHL-5 DLBCL cells and isogenic cells reconstituted with SENP6. Among ~40,000 identified peaks RAD21 binding was altered at 6936 peaks upon SENP6 re-expression, with 3207 peaks exhibiting enhanced RAD21 association in the presence of SENP6 (Supplementary Fig. 11a, b). The alterations were evenly distributed in promoter regions, exons, introns, or intergenic regions (Supplementary Fig. 11b). It is worth noting, that repetitive pericentromeric regions, which represent a major binding region of the cohesin complex, are excluded from the ChIPseq analysis. This likely explains why the chromatin fractionation experiments revealed a more drastic difference in chromatin occupancy of RAD21 than ChIPseq analysis.

To more globally explore SENP6-dependent chromatin association in BCL, we used an unbiased MS-based approach on chromatin fractions isolated from parental SENP6-deficient SU-DHL-5 cells or the SENP6 reconstituted cells. The data revealed an at least 2-fold reduced chromatin association of 58 proteins and additionally identified 6 proteins that were only found at chromatin upon SENP6 re-expression in all three replicates (Fig. 6d, Supplementary Data 4). Notably, SENP6 is among these six proteins indicating that it can restrict SUMO chain formation at chromatin. With the exception of SENP6, the alterations in chromatin association of all other proteins did not correspond to alterations in protein levels in whole-cell extracts (Supplementary Fig. 9b and Supplementary Data 5). Pathway analysis of the 64 proteins that were differentially enriched at chromatin upon SENP6 re-expression shows “separation of sister chromatids”, “mitotic spindle checkpoint” and “cell cycle checkpoints” as the most significant pathways (Fig. 6e). Cohesin core components, such as PDS5B or SMC1B were up to 35% enriched at chromatin upon SENP6 re-expression (Supplementary Data 4). Furthermore, the cohesin establishment factor ESCO2 and Shugoshin 2, which protects centromeric cohesin^31^, exhibit strongly reduced chromatin binding in SENP6-deficient cells strengthening the idea that SENP6 is controlling centromeric cohesion (Fig. 6d, Supplementary Data 4). In line with a more general role of SENP6 in centromere organization, the chromosomal passenger complex (Aurora B and Borealin) as well as the CENP-A loading factor Mis18A, a well-established SENP6 substrate^25,32^, showed impaired binding at chromatin under low SENP6 expression (Fig. 6d, Supplementary Data 4). The finding that Claspin, BARD1 and TopBP1 are also partially lost from chromatin under SENP6-deficiency is in full agreement with the function of SENP6 in the DDR, since these factors are known to cooperate in ATR-CHK1 activation^33,34^. These data demonstrate that SENP6 deficiency in BCL affects chromatin residency of a network of proteins involved in DDR and centromer/kinetochore organization (Fig. 6f).

To further explore whether impaired chromatin association of cohesin complex or other centromere/kinetochore proteins leads to defects in sister chromatid separation, we performed metaphase chromosome spreads from parental SENP6-deficient SU-DHL-5 cells or the SENP6-reconstituted cells. Strikingly, in the vast majority of SENP6-deficient cells, metaphase chromosomes have lost their characteristic X shape and instead appeared as single chromatids (Supplementary Fig. 12).

Altogether, we propose that SENP6 deficiency in BCL triggers polySUMOylation of a larger group of chromatin-associated proteins thereby leading to multifaceted deregulation of chromatin organization and genome integrity.

### SENP6 deletion drives synthetic lethality to PARP inhibition in DLBCL cells

**Fig. 7:**
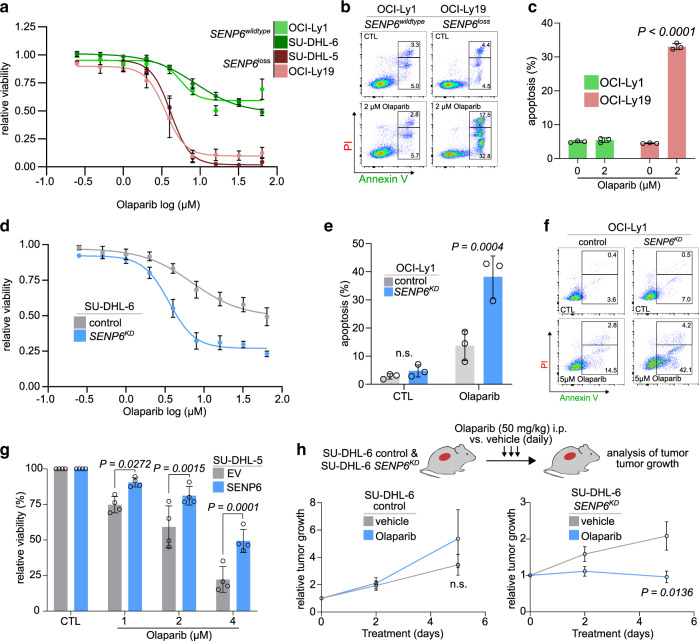
SENP6 deficiency drives synthetic lethality to PARP inhibition. **a** Olaparib dose-response curves for two *SENP6*^wildtype^ and two *SENP6*^loss^ human DLBCL cell lines. Cells were treated for 72 h with olaparib and viability was determined by PI staining and flow cytometry measurement. Data are presented as mean ± SD from *n* = 3 independent experiments. **b** Representative plots of the flow cytometry experiment are described in (**c**). **c** Quantification of flow cytometry results for PI and Annexin V staining in human OCI-Ly1 and OCI-Ly19 DLBCL cell lines after olaparib treatment for 72 h. *P*-value determined by two-way ANOVA; Bonferroni’s multiple comparisons test. Data are presented as mean ± SD from *n* = 3 independent experiments. **d** Olaparib dose-response curves of *SENP6*^*KD*^ and control SU-DHL-6 cells. Cells were treated for 72 h and viability was determined by PI staining and flow cytometry measurement. Data are presented as mean ± SD from *n* = 3 independent experiments. **e** Quantification of flow cytometry results for PI and Annexin V staining of *SENP6*^*KD*^ and control OCI-Ly1 cells after olaparib treatment for 72 h. *P*-value determined by ANOVA; Bonferroni’s multiple comparisons test. Data are presented as mean ± SD from *n* = 3 independent experiments. **f** Representative plots of the flow cytometry experiment are described in Fig. 7e. (**g**) Viability of human SU-DHL-5 SENP6 cells relative to SU-DHL-5 EV control cells after 48 h olaparib treatment with the indicated concentrations. Viability is determined by DAPI staining and flow cytometry measurement. *P*-value determined by two-way ANOVA; Bonferroni’s multiple comparisons test. Data are presented as mean ± SD from *n* = 4 independent experiments. **h** The human SU-DHL-6 *SENP6*^*KD*^ and control SU-DHL-6 cell line was used to generate murine xenograft models in NOD scid mice. Mice were treated with vehicle or 50 mg/kg olaparib by intraperitoneal injection daily and tumor size was measured over time. Tumor size at day 5 of olaparib treated *SENP6*^*KD*^ SU-DHL-6 mice revealed a significant reduction in tumor size in treated mice. SU-DHL-6 control: vehicle (*n* = 5) and olaparib (*n* = 6). SU-DHL-6 *SENP6*:^*KD*^ vehicle (*n* = 5) and olaparib (*n* = 7). *P*-value was determined by unpaired *t*-test (two-tailed). Data are presented as mean ± SEM.

DNA damage repair pathways are commonly compromised in cancers and provide an Achilles’ heel for synthetic lethality. Poly ADP ribose polymerase (PARP) inhibitors (PARPi), such as olaparib, are the prototype of a synthetic lethality-based therapy, for example in breast cancers with BRCA1/2 gene mutations that exhibit homologous recombination repair (HRR) deficiency. PARPi blocks alternative DNA repair pathways on which cancer cells with compromised HRR rely^35^. In addition to BRCA1/2, other genetic determinants of PARPi sensitivity have been identified^36^. This includes components of the cohesin complex as well as other DDR factors implicated in the resolution of replication fork stalling, such as TopBP1^37^. Based on our finding that SENP6 depletion affects cohesin functions and the DDR response, we asked whether SENP6-deficient DLBCL cells were vulnerable to PARPi. Indeed, *SENP6*^loss^ DLBCL cells were sensitive to olaparib treatment, whereas *SENP6*^wildtype^ DLBCL cells were less sensitive (Fig. 7a). Similarly, olaparib increased apoptotic cell death of *SENP6*^loss^ cells but had minimal effect on *SENP6*^wildtpye^ cells (Fig. 7b, c). Of note, CRISPR/Cas9-mediated depletion of SENP6 in SU-DHL-6 (Supplementary Fig. 13a, b) and OCI-Ly1 cells led to a striking increase in olaparib sensitivity when compared to control cells, demonstrating that olaparib-induced cell death is dependent on the SENP6 status (Figs. 7d and S13c). Accordingly, we observed increased apoptotic cell death in SENP6-depleted OCI-Ly1 cells after olaparib treatment (Figs. 7e, f and S13d). In support of this observation, ectopic expression of SENP6 increased the resistance of SU-DHL-5 cells to olaparib treatment in comparison to SU-DHL-5 EV control cells (Figs. 7g and S13e, f).

We anticipated that the phenotypes and PARPi sensitivity of SENP6-deficient BCL cells are at least partially caused by unscheduled activation of the RNF4 pathway resulting from unrestricted polySUMOylation^23,25,38,39^. Given the abundant expression of *Rnf4* in MYC-driven lymphomas (Supplementary Fig. 14a), we hypothesized that co-depletion of RNF4 and SENP6 may impair PARPi sensitivity. Indeed depletion of RNF4 reduced the sensitivity to PARPi triggered by SENP6 depletion (Supplementary Fig. 14b–d).

To promote clinical translation of the SENP6 status as a biomarker for PARPi treatment in DLBCL, we generated xenografts of control and *SENP6*^*KD*^ SU-DHL-6 cells. Upon tumor formation, we treated the experimental cohorts with either vehicle control or olaparib and monitored tumor growth (Fig. 7h). Whereas olaparib treatment did not affect the growth of SU-DHL-6 control cells, we observed significantly reduced growth of SU-DHL-6 *SENP6*^*KD*^ xenograft tumors (Fig. 7h). Altogether, these data reveal PARPi as a potential therapeutic option for SENP6-deficient DLBCL.

## Discussion

We performed a genome-scale discovery screen to identify genes that accelerate MYC-driven B-cell lymphomagenesis using a murine model. Starting from this approach, we identified recurrent *SENP6* deletions in human BCL and provided direct experimental evidence that SENP6 loss-mediated hyperSUMOylation accelerates lymphomagenesis. Moreover, we demonstrate that SENP6 loss affects the chromatin association of protein complexes implicated in the response to replication stress and the repair of damaged or stalled replication forks. We propose that this induces genomic instability and sensitizes cells to PARP inhibitors, pointing to therapeutic options for a subgroup of patients with BCL.

Deletions of *SENP6* were recurrently found in human BCL. Comprehensive sequencing studies have shown that hundreds of genes are altered in BCL with frequencies comparable to that of *SENP6* deletions^10^. In order to interpret and translate findings from such sequencing studies into mechanism-based therapeutic options for patients, functional studies thus seem indispensable.

Although several studies have provided evidence that SUMO conjugation is activated in human cancers^1,6,7^, we here show experimentally that aberrant polySUMOylation caused by the loss of *SENP6* promotes tumorigenesis in vivo. With regard to MYC-dependent cancers, we found a strong SENP6-dependent increase of SUMO2/3 conjugates, revealing that SUMO conjugation is activated either by induction of the SUMO conjugation machinery or by loss of the cellular safeguard deSUMOylase SENP6. Previous work suggests that enhanced SUMOylation in MYC-driven tumors is needed for a distinct gene expression program^6^. However, we provide compelling evidence that the enhanced polySUMOylation, which is triggered by SENP6 deficiency, primarily affects genome stability in BCL. In addition, we also identified CIS including *Senp1*, however, *SENP1* was not affected by a genetic alteration in human BCL and the potential relevance of SENP1 in human cancers needs to be investigated in further studies

Insights into the cellular caretaker mechanisms allowing tumor cells to handle increased levels of DNA damage are crucial for the rational development of mechanism-based therapeutic strategies. This is of particular interest in BCL, where DNA DSBs represent a physiological process during the formation and modification of specific antigen receptors and where failure in the repair of DNA breaks is crucial for malignant transformation. Here, we demonstrate that SENP6 is a crucial caretaker protein in BCL that functions as a deSUMOylase of chromatin-associated proteins that are instrumental for the control of genome stability. Accordingly, SENP6 is critical for the genome integrity in murine as well as in human lymphomas, and deficiency of SENP6 leads to a significant increase of somatic DNA copy number variations. Moreover, we find that low SENP6 expression is associated with poor outcomes of DLBCL patients after standard treatment and that the SENP6 status affects the sensitivity of DLBCL cells to chemotherapy.

Our biochemical and cell-biological data indicate that SENP6 deficiency in BCL impairs chromatin residency of a network of proteins involved in centromer/kinetochore organization and the DDR. We propose that the cohesin complex is one potential target of SENP6-mediated tumor suppression. The cohesin core subunits RAD21, STAG1, and STAG2 are well-known tumor suppressors in human cancers^19^, and the corresponding murine genes were identified as tumor suppressor genes in our *Eµ-myc* transposon mutagenesis screen. Cohesins hold sister chromatids together until metaphase to anaphase transition. In addition, cohesins are present at replication sites and promote restart of stalled replication forks thereby promoting replication stress tolerance^40^. Finally, cohesins are also involved in homologous recombination-mediated DSB repair^41^. In accordance with our previous work^23^, we demonstrate impaired chromatin association of RAD21 and STAG2 in a human BCL cell line deficient for SENP6. Moreover, our unbiased proteomics experiments in this cellular system revealed a strongly reduced chromatin association of ESCO2 and Shugoshin 2, which control the establishment and protection of centromeric cohesin, respectively^31^. Altogether, these data support the idea that SENP6 deficiency affects chromatin loading or maintenance of the cohesin complex, thereby affecting chromosome stability. Notably, however, in line with published work, SENP6 deficiency generally affects centromere/kinetochore organization indicating that the observed phenotypes reflect combinatorial effects resulting from the disturbance of several pathways. This interpretation is in line with the recently proposed model of SENP6-catalyzed SUMO group de-modification of centromeric proteins^25^. Importantly, SENP6 is not only implicated in centromere/kinetochore organization but is also a critical determinant for proper DNA damage checkpoint activation in BCL. We show that in a BCL cell line deficient for SENP6, ATR-CHK1 activation is strongly impaired. ATR is recruited to stalled replication forks and is activated by its cofactor ATRIP, which binds to RPA‐coated single‐stranded DNA. Recruitment of ATRIP and the activation of the ATR-CHK1 axis requires an intertwined network of regulators at DNA lesions. ATRIP recruitment is initiated by TopBP1 and facilitated by the hPSO4/PRP19 complex, while Claspin promotes ATR-dependent phosphorylation of CHK1^34^. Intriguingly, our unbiased MS data demonstrate that SENP6 deficiency disturbs the network of ATR-CHK1 regulators. We show that the chromatin association of TopBP1, the hPSO4 subunit Cdc5L, and Claspin are strongly reduced in SENP6-deficient cells. The observed loss of these factors from chromatin during oncogene-induced replicative stress after depletion of SENP6 provides a molecular rationale for compromised DNA damage checkpoint activation. The loss of the HR factors RAD51C, RAD54, and MMS22L which we detected upon SENP6 deficiency, further indicates that SENP6-dependent deSUMOylation affects several pathways associated with DDR and genome maintenance and acts as a central signaling hub controlling critical DDR signaling pathways. We further conclude that the caretaker function of SENP6 in these pathways is linked to its role as a rheostat of chromatin occupancy. PolySUMOylation in conjunction with RNF4-mediated ubiquitylation normally initiates the release of genome maintenance factors from sites of DNA damage upon the termination of the DDR^42,43^. In the early response phase, SENPs counterbalance SUMOylation to avoid premature release or limit turnover. Our data suggest that in analogy to what was proposed for SENP2, SENP6 is required to restrict an “over before it has begun” repair response^42^. SENP6 loss consequently impairs DNA repair and causes genomic instability. The observation that RNF4 depletion does not totally abrogate PARPi sensitivity of SENP6-deficient DBCL lines is in agreement with the finding that at least for some targets polySUMOylation alone can affect their chromatin association without the subsequent RNF4-dependent ubiquitylation.

A striking observation of our study is the sensitivity of SENP6-deficient BCL cells to PARPi. Following the initial discovery of synthetic lethality between PARP inhibition and BRCA1 or BRCA2 deficiency, other cancer types that harbor mutations in genes functioning in the DDR and DNA repair networks were also shown to be sensitive to PARP inhibition^44^. This includes mutations in components of the cohesin complex as well as other DDR factors, such as TopBP1^37,45^. It has been proposed that inhibition of replication fork stability by PARPi creates a synthetic lethality in cancer cells deficient of STAG2^46^. The finding that loss of SENP6 impaired cohesin function and DDR activation is therefore in perfect agreement with the observed sensitivity of SENP6-deficient DLBCL to PARPi. Because several PARP inhibitors are approved for clinical use in other indications, PARPi could rapidly be investigated in clinical trials as a therapeutic option for patients with SENP6-deficient lymphoma.

In summary, starting from an in vivo cancer gene discovery screen, we have identified *SENP6* deletions and associated unrestricted SUMOylation as a functional and clinically important driver of lymphomagenesis, and we have demonstrated SENP6’s downstream effectors as potential effective lymphoma treatment targets.

## Methods

### Mice

*ATP2-H32* transgenic mice were crossed to *Eµ-myc* mice to generate the *ATP2-H32;Eµ-myc* line. The resulting line was crossed to *Rosa26*^*PB*^ mice to generate triple-transgenic *Eµ-myc;ATP2-H32;Rosa26*^*PB/+*^ mice. *Eµ-myc* (002728) mice were purchased from the Jackson Laboratory. Mice were examined twice a week and sacrificed as soon as lymph nodes were well palpable (5 mm diameter) or any of the approved thresholds were reached. The survival data in each experiment were analyzed using the Kaplan–Meier method and the log-rank (Mantel–Cox) test was applied to test statistical significance. All animal experiments were performed in accordance with local authorities (Regierung von Oberbayern, Munich, Germany).

### Transplantation experiments

For the in vivo validation of the tumor suppressor function of SENP6, a single guide RNA (sgRNA) sequence (5′-AGAGGAAAGTCCAGCAGAAG-3′) was designed and selected with the CHOPCHOP (http://chopchop.cbu.uib.no/) sgRNA design resource and cloned into the pLKO5.sgRNA.EFS.GFP (Addgene #57822) construct. Transduction-transplantation experiments have been performed as described before^17^. The transduction efficacy was typically between 15–25% and 2.5 × 10^5^ eGFP-positive HSPCs and 2 × 10^5^ CD45.1 bone marrow helper cells were transplanted into lethally irradiated (8.5 Gy) recipient mice. For all transplantation experiments female C57Bl6/J mice aged 6–8 weeks were used. Mice were purchased from Charles River. After transplantation mice were monitored daily for lymphoma development.

### Xenograft experiments

The tumor cells were suspended in RPMI, mixed 1:1 with Matrigel (BD Biosciences), and transplanted subcutaneously into the flanks of immunocompromised NOD SCID mice (Charles River). 10 × 10^6^ SU-DHL-6 control or *SENP6*^*KD*^ cells were used per mouse. Mice were monitored daily and tumors were measured using calipers three times per week. The metric tumor volume (*V*) was calculated by measurements of length (*L*) and width (*W*) by using the following equation: *V* = 0.5 × (*L* × *W*^2^). Treatments were started when the tumors were actively growing, judged by increasing volumes on repeated caliper measurements. Olaparib was dissolved in 4% DMSO + 30% PEG300 + H_2_O and mice were treated by daily intraperitoneal injection with either 50 mg/kg body weight per dose or vehicle. The maximal permitted tumor size was a diameter of 1.5 cm and the maximal tumor size was not exceeded.

### Quantitative transposon insertion site sequencing and DNA isolation

Infiltrated lymph nodes from triple transgenic mice were harvested and snap-frozen. Genomic DNA was extracted using the Qiagen blood and tissue kit according to the manufacturer’s instructions. Transposon insertion sites were recovered using the QiSeq pipeline as described earlier^17^. Statistical analysis for CIS identification using CIMPL (Common Insertion site Mapping Platform) analysis based on a Gaussian kernel convolution framework was performed as previously reported^17^.

### Flow cytometry

Flow cytometry analysis was performed following standard protocols. Primary cell suspensions from lymph nodes were directly labeled with fluorescently labeled antibodies against the following surface proteins: CD45 (anti-mouse CD45, FITC, 30-F11), B220 (anti-mouse/anti-human CD45R (B220), PE-Cyanine7, RA3-6B2), IgM (anti-mouse IgM, PE, eB121-15F9). Data were acquired using a Beckman Coulter CyAn or CytoFLEX flow cytometer and analyzed by FlowJo software (FlowJo LLC).

### Fluorescence microscopy

Cells were cultured on poly-l-lysine-coated slides for 3 days and fixed with 4% paraformaldehyde (Merck Millipore) in PBS. Slides were blocked with 10% FCS (PAA, Germany), 0.1% Triton‐X (Carl Roth) in PBS, and stained with primary phospho-Histone H2A.X (Ser139) (Millipore). As a secondary antibody, we used goat anti-mouse IgG (H + L) Superclonal™ Secondary Antibody, Alexa Fluor 488 (Thermo Fisher). All stains were counterstained with SlowFade^®^ Gold Antifade Reagent with DAPI (Thermo Fisher). Staining was assessed on a Leica DM RBE fluorescent microscope (Leica). Fluorescence intensities of stained cells were quantified in total pixels from at least 15 cells after background correction using ImageJ (NIH). Each stain included a negative Ig control, the detected pixels of which were deducted from the total pictures as background.

### Chemicals

Doxycycline hyclate (D9891) was purchased from Sigma (Sigma, Munich, Germany). A concentration of 1 µg/ml was used to induce MYC expression in U-2-OS cells with a doxycycline-inducible MYC construct. Olaparib was purchased from Selleck, USA, and LC Laboratories, USA.

### Immunohistochemistry on TMAs of human DLBCL samples

For TMA construction, formalin-fixed and paraffin-embedded tissue from 75 patients suffering from DLBCL were used. Patients were treated during a time period from 1991 to 2019 in the University Clinic of the Technical University Munich. Corresponding to the remission status, patients were divided into two biological groups, either showing long-term remission (duration of remission >2 years; 37 patients) or early relapse (<1 year)/refractory disease 38 patients). Evaluation of SENP6 and SENP7 immunohistochemistry was performed by a certified pathologist, blinded to clinical data. Expression was assessed based on the percentage of stained tumor cells, irrespective of the staining intensity, and scored as 0 (no expression), 1 (<10%), 2 (10–50%), 3 (51–80%), and 4 (>80%). Tumor samples with scores 0–2 were considered as SENP6^low^ or SENP7^low^, and samples with scores 3–4 as SENP6^high^ or SENP7^high^. Informed consent for the scientific use of biopsy material was obtained from patients. The responsible ethics committees of the Technische Universität München approved data analysis (ethics approval 498/17 s).

### Histology

Mouse lymph nodes were fixed in 10% neutral-buffered formalin solution for min. 48 h, dehydrated under standard conditions (Leica ASP300S, Wetzlar, Germany) and embedded in paraffin. Serial 2 µm-thin sections prepared with a rotary microtome (HM355S, Thermo Fisher Scientific, Waltham, USA) were collected and subjected to histological and immunohistochemical analysis. Hematoxylin–Eosin (H–E) staining was performed on deparaffinized sections with Eosin and Mayer’s Haemalaun according to a standard protocol.

### Immunohistochemistry

Immunohistochemistry on human TMAs and whole slide specimen as well as on murine tissues was performed using a Bond RXm system (Leica, Wetzlar, Germany, all reagents from Leica) with primary antibodies diluted as mentioned in the supplemental methods. Briefly, slides were deparaffinized using deparaffinization solution and pretreated with Epitope retrieval solution. Antibody binding was detected with a polymer refine detection kit without post-primary reagent and visualized with DAB as a dark brown precipitate. Counterstaining was done with hematoxylin.

### Analysis of CRISPR/Cas9 target regions

Genomic DNA from infiltrated lymph nodes was isolated using the Quiagen Blood and Tissue kit. PCR amplification of targeted loci was carried out with Q5^®^ Hot Start High Fidelity 2× Master Mix. Afterward, the PCR products were analyzed using Engen T7 Endonuclease I following the manufacturer’s protocol.

### Metaphase spreads

To microscopically analyze chromosome spreads, SU-DHL-5 cells grown in suspension culture were treated with RO-3306 (5 μM) for 16 h. Subsequently, cells were washed three times with a pre-warmed RPMI medium and further treated with colcemid (0.1 μg/ml) for 4 h. Subsequently, cells were washed once with pre-warmed PBS followed by incubation in hypotonic solution (75 mM KCl) for 20 min at 370 °C. Cells were further pelleted down, treated with freshly prepared Carnoy’s fixative [75% (v/v) methanol, 25% (v/v) acetic acid], incubated at room temperature for 30 min, and kept at −200 °C overnight. Next, cells were pelleted down and treated again with freshly prepared Carnoy’s fixative. After finally pelleting the cells down, the fixative was removed keeping 1 ml at the bottom. 50–200 μl of cell suspension was dropped from a distance of 0.5 m onto wet slides kept in a slanted manner. The slides were then air-dried and the nuclei were stained with DAPI (1 min) followed by mounting of coverslips using Prolong Gold anti-fade reagent as the mounting medium. The slides were further subjected to microscopic analysis.

### Gene expression analysis

Gene expression data were retrieved from the Gene Expression Omnibus (GEO) using the accession numbers GSE44672^28^ and GSE12195^21^, respectively. For the former, normalized counts supplied by the authors were log2 transformed before downstream analysis. For the latter, all CEL files were retrieved and normalized using the GCRMA R package. Differential gene expression (DEG) analysis between conditions was carried out using the limma framework^47^ and an FDR < 0.1 was considered significant. Selected DEG results were illustrated in a heatmap using the pheatmap R package after scaling all genes to have a mean of 0 and a standard deviation of 1.

### Viral infections and cell culture

NIH-3T3, HEK293T, U-2-OS, and Phoenix-Eco cells were cultured in DMEM with 10% FCS. Human DLBCL cell lines were cultured in RPMI-1640 or IMDM medium supplemented with 20% FCS and 2 mM l-glutamine. SU-DHL-5, SU-DHL-6, OCI-Ly1, and OCI-Ly19 cells were purchased from DSMZ (Leibniz Institute DSMZ-German Collection of Microorganisms and Cell Cultures). U-2-OS cells with inducible MYC expression have been described^28^. For the generation of lentiviral particles, HEK293T cells were co-transfected with the indicated lentiviral plasmids and helper virus plasmids (Lipofectmanie 2000, Invitrogen). For shRNA knockdown, specific short hairpin RNA (shRNA) constructs targeting human *RNF4* or murine *Senp6* were ordered from Sigma MISSION (Senp6.21: TRCN0000031021, Senp6.22: TRCN0000031022, Senp6.24: TRCN0000031024, RNF4: TRCN0000017056). These plasmids are based on plko.1. The puromycin^R^ gene was replaced by an eGFP complementary DNA. For the generation of ecotropic retroviral particles, Phoenix-Exo cells were transfected with the indicated retroviral plasmids. Virus supernatants were collected 48 h after transfection and used to transduce the indicated cell lines in the presence of 1 µg/ml polybrene (Sigma-Aldrich). Suspension cells were transduced using spin-transduction at 400 × g for 1 h at 32 °C.

### CRISPR/Cas9-based generation of SENP6 depleted cell lines

For depletion of SENP6 in human DLBCL cell lines, exon 2 of the SENP6 open-reading frame was removed by CRISPR/Cas9 gene editing. To this end, 150,000 OCI-Ly1 or SU-DHL-6 cells were transfected with 500 ng of each of the sgRNA’s and 1 µg Cas9 protein (PNA Bio) with a Neon Transfection System (Thermo Fisher/Invitrogen) (parameters: 1450 V; 10 ms; 4 pulses). The cleavage efficacy was tested 24 h following transfection with the Terra™ PCR Direct Card Kit and primers flanking exon 2. Cells were then separated into single cells by serial dilution. Cell clones were screened for efficient gene editing and selected clones were analyzed for SENP6 protein expression by immunoblot analysis. Used sgRNAs: SENP6Ex2_g2:AGATCAGAGTCTAAGAGAGA, SENP6Ex2_g3:GGAGATACAGATAAAGAGTA.

### Cell fractionation

Cell fractionation of U-2-OS cells has been performed as described in ref. ^48^. The chromatin fraction was purified by resuspending the final pellet in 0.2 N HCl and incubating for 20 min on ice. Afterward, the suspension was neutralized with Tris–HCl (pH 8). Chromatin fractionation in SU-DHL-5 cells was performed according to Kustatscher et al. with minor modifications as detailed in sample preparation for proteome analysis^49^.

### Immunoblot analysis

Protein extracts were prepared by incubating cell pellets in lysis buffer (50 mM HEPES, 150 mM NaCl, 1 mM EDTA, 2.5 mM EGTA, and 0.1% Tween) supplemented with NaF, PMSF and NaVO_4_ followed by sonification. For analysis of SUMOylation, 20 mM of N-Ethylmaleimide (NEM) was added to inhibit SUMO-proteases. Protein lysates were fractioned on SDS–PAGE gels, transferred to Immobilon-P (Millipore) membranes, and incubated with the specific antibodies listed below.

### Sample preparation for proteome analysis

Cells were lysed in 2% SDS lysis buffer, shortly heated to 95 °C, then sonicated and centrifuged at 16,000×*g* for 5 min. In the following, protein content was determined using the DC Protein Assay Kit from BioRad. For in-solution digestion, 20 µg of each sample were precipitated using 4 volumes of acetone for 1 h at −20 °C. After centrifugation, a wash step with 90% acetone was included. The precipitated pellet was shortly dried at room temperature and then resuspended in 6 M urea/2 M thiourea. Proteins were reduced with DTT, following an alkylation step using chloroacetamide. Digestion was performed in only 2 M urea with the endopeptidase Lys-C (Wako) in combination with trypsin (sequence grade, Promega) overnight at 37 °C. Digestion was stopped by acidifying. Finally, peptides were desalted and concentrated by the STAGE tipping technique (Stop and Go Extraction) described by Rappsilber et al.^50^.

### Mass spectrometry of chromatin-associated proteins

Chromatin fractionation in SU-DHL-5 cells was done according to Kustatscher et al.^49^. The chromatin-associated proteins were subsequently subjected to Filter-Aided Sample Preparation for proteome analysis as described in Wiśniewski^51^. Tryptic peptides were subsequently desalted using the STAGE technique^50^. Proteomic analyses were performed on an Easy nano-flow UHPLC system (Thermo Fisher) coupled to Q Exactive HF mass spectrometer (Thermo Fisher). The mass spectrometer was operated in a data-dependent mode (MS scans, 300–1650*m*/*z*). Full-scan MS spectra of proteomic samples were acquired using 3E6 as an AGC target with a resolution of 60,000 at 200*m*/*z* with a maximum injection time of 20 ms. The 15 most intense ions were fragmented by high collision-induced dissociation (HCD). Resolution for MS/MS spectra was set to 15,000 at 200*m*/*z*, AGC target to 1E5, maximal injection time to 25 ms.

All acquired raw files of mass spectra were analyzed using MaxQuant Software (version 1.6.17.0)^52^ and the implemented Andromeda database search engine^53^. Fragmentation spectra were correlated with the Uniprot human database (v.2015/2017). To perform searches, tryptic digestion and default settings for mass tolerances of MS and MS/MS spectra were applied. False discovery rate (FDR) was set to 1%, minimal LFQ ratio count was set to 2 and FastLFQ option was enabled for relative label-free quantification of proteins. For the analysis, the match between run features was used.

MS data analysis and statistics were done with the Perseus software (version 1.6.15.0). First, contaminants and reverse entries, as well as proteins only identified by a modified peptide were removed. The log2 value of all LFQ intensities was calculated. Using the histogram analysis function of the software, the normal distribution of the LFQ values was visually checked. Good correlation of the experimental replicates was assured by multi-scatterplot analysis. Samples were then grouped into triplicates and a Student’s *t*-test was performed with randomization of 500 and an s0 factor of 0.1. Then the datasets were exported and used for further analysis in Microsoft Excel. Significant enrichment was defined in Excel based on the *P*-value and the Student’s *t*-test difference applying the following criteria: −log 10 *P*-value > 1.3 and log2 ratio ≥ 1 or ≤−1. Visual representation of data in volcano plots was done using the R Studio software.

### Generation of STRING network

The freely available STRING software (version 11.0) was used to generate the STRING networks. For network analysis, proteins that are exclusively present in three replicates of SENP6 reconstituted SU-DHL-5 cells and enriched significantly at least 2 folds after SENP6 reconstitution was chosen. For all analyses, we set the parameters to the highest confidence and used an MCL clustering with an inflation of 3. Experiments and databases were enabled. Non-connected proteins were excluded from the visualized interaction network. Cytoscape (version 3.8.2) was further used to process the STRING network.

### Quantitative RT-PCR

RNA was isolated using the RNeasy Plus Mini Kit (Qiagen). Complementary DNA (cDNA) was prepared using the Omniscript RT kit (Qiagen). qPCR was performed using a TaqMan cycler (Applied Biosystems) and the Platinum SYBR Green qPCR SuperMix-UDG kit (Invitrogen) and analyzed using the ΔΔCt method with control samples set as 1. Primers used for RT-PCR: *Myc* (fw: TTCCTTTGGGCGTTGGAAAC, rv: GCTGTACGGAGTCGTAGTCG), *Senp6* (fw: CGGCACTGTAGCACTTACCA, rv: GGCTTGTCGGCAATTTCTT), *Senp7* (fw: GGAT-GTTCTTGCTCAGTCACC, rv: ACCTTGCTGGGAGCACATAA), *Ubiquitin* (fw:GCAAGTGGCTAGAGTGCAGAGTAA, rv: TGGCTATTAATTATTCGGTCTGCAT), *RNF4* (fw: GGATACTCAGAGATCGTGCAGA, rv: AGGCACTGGCTACAGAAGACA), *GAPDH* (fw: GGTATCGTGGAAGGACTCATGAC, rv: ATGCCAGTGAGCTTCCCGTT-CAG).

### RNA sequencing

Cells and RNA isolation were prepared as described for RT-PCR. RNA quality was assessed with Agilent RNA 6000 Pico Kit according to the manufacturer’s instructions in the Agilent Bioanalyzer 2100. RNA concentration was determined with a Nanodrop spectrophotometer. For SU-DHL-5 EV and SENP6 cells, total RNA was enriched in mRNA with NEBNext Poly(A) mRNA Magnetic Isolation (NEB, 187 # E7490). Libraries were prepared with NEBNext Ultra II Directional RNA Library (NEB, 188 #E7765L) and indexes were added by PCR with NEBNext Multiplex Oligos for Illumina (NEB, 189 #E7600) according to the manufacturer’s protocols. Libraries were quantified and checked for fragment size with Agilent High Sensitivity DNA Kit (Agilent Technologies). They were pooled in equimolar ratios and sequenced on an Illumina NextSeq500 for 75 bps in a single-ended fashion. Raw reads were quality checked, adapters trimmed using Trimmomatic v0.36. Reads were aligned to the human reference genome (GRCh38) using HISAT2 with default 194 parameters. GSEA was carried out on a Wald statistic differential gene expression signature using the fgsea R package. Gene sets were retrieved from the MSigDb v7.3^54,55^. Enrichment results for select pathways were illustrated using the fgsea package. For OCI-Ly1 control and SENP6^KD^ cells, library preparation and paired-end sequencing was performed by Novogene (Cambridge, UK) on a HiSeq2500 (Illumina, San Diego, CA) with a sequencing depth of more than 20M reads/sample. The resulting Fastq files were mapped to the human reference genome hg38 with STAR^56^. Reads were estimated for each transcript using the transcript sequences from the human reference hg38/GRCh38 and the Salmon software (v1.3.0)^57^. Counts were normalized and differential gene expression has been analyzed by DeSeq2^58^. Normalized count tables were subsequently used for GSEA, using the Kolmogorov–Smirnov test and Hallmark Signatures of the Molecular Signature Database^54^ implemented in GeneTrail 3.0^59^.

### Spike-in ChIP-sequencing

ChIP-seq was performed as described previously^60^. In brief, 50 million SU-DHL-5 cells per IP condition were fixed using formaldehyde at 1% final concentration for 5 min at room temperature and fixation was stopped with the addition of 125 mM glycine for 5 min at room temperature. After washing, cells were lysed in lysis buffer I (5 mM PIPES pH 8, 85 mM KCl, 0,5% NP40, 10 mM Glycine), and 6% murine T-lymphoma^MYC-Tet-Off^ cells were added for exogenous spike-in. After 20 min lysis, nuclei were collected (400 × *g*, 15 min, 4 °C). Nuclei were incubated in lysis buffer II (RIPA Buffer: 10 mM Tris/HCl pH 7.5, 150 mM NaCl, 1 mM EDTA, 1% NP-40, 1% deoxycholic acid sodium salt, 0.1% SDS) for 10 min. Crosslinked chromatin was fragmented by sonication (total duration: 20 min, pulse of 10 s with 45 s pausing) or using the Covaris Focused Ultrasonicator M220 for 100 min per ml lysate. Efficient chromatin fragmentation was confirmed by agarose gel electrophoresis. Prior to immunoprecipitation, chromatin was cleared (20 min, 15,000 × *g*, 4 °C). Per IP reaction 100 µl Dynabeads (Protein A and Protein G 1:1 mixture, Thermo Fisher Scientific) were incubated overnight with 15 µg of the corresponding antibody in presence of 5 g/L BSA in PBS: SUMO1 (abcam, ab32058), SUMO2/3 (abcam, ab81371), MYC (abcam, ab32072), and RAD21 (Bethyl Laboratories, A300-080A) antibodies. Chromatin corresponding to 50 million cells per IP reaction was added to the beads and IP was performed for 6–8 h on rotating wheel (4 °C). After IP, beads were washed with washing buffer I (20 mM Tris–HCl pH 8,1; 150 mM NaCl; 2 mM EDTA; 0.1% SDS; 1%Triton-X-100), washing buffer II (20 mM Tris–HCl pH 8.1; 500 mM NaCl; 2 mM EDTA; 0.1% SDS; 1%Triton-X-100), washing buffer III (10 mM Tris–HCl pH 8,1; 250 mM LiCl; 1 mM EDTA; 1% NP-40; 1% deoxycholic acid sodium salt), and TE buffer three times each. Elution was performed twice in 150 µl elution buffer (1% SDS, 0.1 M NaHCO_3_) each for 15 min on rotating wheel (room temperature). De-crosslinking of eluted samples and input samples was carried out overnight, and RNA and proteins were digested by adding RNase A and proteinase K, respectively. After chloroform–phenol extraction and ethanol precipitation, DNA concentration was determined using the Quant-iT PicoGreen dsDNA assay (Thermo Fisher Scientific).

For ChIP-seq library preparation the NEBNext ChIP-Seq Library Prep Master Mix Set for Illumina (New England Biolabs) or NEBNext Ultra II DNA Library Prep with Sample Purification Beads (New England Biolabs) were used according to the manual’s instructions. Library quality was determined using the Fragment Analyzer (Advanced Analytical; NGS Fragment High Sensitivity Analysis Kit (1–6000 bp; Advanced Analytical)) prior to being sequenced on the Illumina Next-Seq500.

### ChIP-sequencing analysis

FASTQ file generation was carried out using Illumina CASAVA software within BaseSpace suit. Quality control of FASTQ files was performed using FASTQC. Reads were then aligned to hg19 build of the human reference genome and mm10 child of mouse genome for calculating the amount of spike in. Since the spike in reads was the same in all samples, the files were read-normalized to the same depth and used for further analyzes after combining the input samples. The read-normalized bam files were converted to bedGraphs for visualization in a genome browser. Peak calling was carried out using macs v1.4 and peak annotation was performed with bedtools v2.29.0 suit (Parameters: *P*-value*:* 1e−6; keepdup: 5). The density plots were generated with deep tools v3.3.1.

#### Antibodies

For western blotting: SUMO1 (1:1000, rabbit, Cell Signaling #4930), SUMO2/3 (1:1000, rabbit, Cell Signaling #4971), c-MYC (sc-764) (1:500, rabbit, Cell Signaling #9402), β-Actin (1:5000, mouse, Sigma-Aldrich A5316), RAD21 (1:1000, mouse, Santa Cruz sc-271601), STAG2 (1:1000, mouse, Santa Cruz sc-81852), p-CHK1 (1:1000, rabbit, Cell Signaling #2348), CHK1 (1:1000, mouse, Santa Cruz sc-56291), уH2AX (1:2000, rabbit, Abcam ab11174), CDC5L (1:1000, rabbit, Atlas HPA011361), PRP19 (1:1000, mouse, Santa Cruz sc-514338),Histone-H3 (1:2000, rabbit, Cell Signaling #4499), SENP6 (1:500, rabbit, Sigma-Aldrich HPA024376), SENP7 (1:500, rabbit, Abcam ab58422), β-Tubulin (0.4 µg/ml, mouse, DSHB #E7), mouse IgG HRP (1:10,000, sheep, GE Healthcare #NA931V), rabbit IgG HRP (1:10,000, donkey, GE Healthcare #NA934V), RNF4 (1:2000, rabbit, provided by A. Vertegaal), Vinculin (1:1000, rabbit, Cell Signaling #13901). For immunohistochemistry: SENP6 (1:100, rabbit, ER1 20 min pre-treatment, Sigma-Aldrich HPA024376), SENP7 (1:100, rabbit, ER1 30 min pre-treatment, Sigma-Aldrich HPA027259), IgM (1:75, rat, ER2 30 min pre-treatment, BD Biosciences #553435),B220 (1:50, ER1 20 min pre-treatment rat, BD Biosciences #550286). For flow cytometry: CD45 (30-F11, 1:50, rat, eBioscience #53-0451-82), B220 (PE-Cyanine7, RA3-6B2, 1:50, rat, eBioscience #25-0452-82), IgM (PE, eB121-15F), 1:50, rat, eBioscience #12-5890-82), AnnexinV (APC, 1:25, BioLegend #640919) For ChiP: SUMO1 (15 µg, rabbit, Abcam ab32058), SUMO2/3 (15 µg, mouse, Abcam ab81371), MYC (15 µg, rabbit, Abcam ab32072) RAD21 (15 µg, rabbit, Bethyl Laboratories A300-080A). For fluorescence microscopy: phospho-Histone H2A.X (Ser139) (2 µg/ml, mouse, Millipore #05-636), anti-Mouse IgG (H + L) Superclonal™ Secondary Antibody (Alexa Fluor 488, 1 µg/ml, goat, Thermo Fisher #A28175).

### Pathway enrichment analysis and GSEA

Pathway enrichment analysis was performed by GeneTrail2^20^ (using Reactome database) and was assessed using an over-representation analysis. Corresponding *P*-values are FDR-adjusted per database using the method of Benjamini and Yekutieli with a significance level of 0.05. Results obtained with Reactome have been visualized with Adobe Illustrator. GSEA was performed with the H gene sets from MSigDB (http://www.broadinstitute.org/gsea/msigdb) by GeneTrail2. Gene sets related to MYC signatures were selected. Gene set enrichment was assessed using the Kolmogorov-Smirnov test. Corresponding *P*-values were FDR-adjusted per database using the method by Benjamini and Yekutieli with a significance level of 0.05.

### Bioinformatic tools

The freely available STRING database https://string-db.org (version 10.5) was used for the generation of string networks. The following criteria were used for network analysis: Recently described SENP6 target protein datasets were used as input^23,25^. The parameters were set to the highest confidence and we used an MCL clustering with inflation of 3. All active interaction sources were enabled and non-connected proteins were excluded from the visible interaction network. VENN diagrams were generated using an online platform (https://www.meta-chart.com/venn). In the case of enrichment analysis *P*-values were determined with Fisher’s exact test.

### Analysis of CNAs in murine BCLs

Genomic DNA was isolated with DNeasy Blood & Tissue Kit. Library preparation was performed with 50 ng DNA per sample using the Illumina Nextera DNA Flex Library Prep Kit. Samples were sequenced single end with 75 bp reads on an Illumina NextSeq system. The resulting sequencing data were processed using a standardized set of pipelines^61^. Briefly, reads were trimmed using Trimmomatic and mapped to the mouse reference genome GRCm38.p6 using bwa mem. The GATK toolkit was used for base recalibration. CNAs were called by HMMCopy, using data from the tail of backcrossed C57BL/6J mice as control. For a detailed description, we refer to the Nature Protocol by Lange et al. ^61^.

### Analysis of CNAs in human BCL

To assess the *SENP6* copy number in a recently published human dataset^10^, we first queried if *SENP6* is targeted by a GISTIC2-identified recurrent SCNA or an arm-level SCNA by interrogating the available information from the published human dataset. Next, we classified the patients in groups in which the respective CNAs were absent or present and compared *SENP6* mRNA expression and driver SCNAs leveraging the respective data within this study. For analysis for *SENP6* mRNA expression, we analyzed both probes detecting normal length SENP6 transcripts (202319_at und 202318_s_at). To investigate genomic events, which are associated with SCNAs affecting the *SENP6* locus, we tested the co-occurrence of all other genomic events with 6q:DEL and 6q14.1:DEL, respectively, in a Chi-squared contingency table test. Similarly, we tested the association of 6q:DEL with previously described DLBCL clusters^10^ using Pearson’s Chi-squared test. To test the enrichment of transcriptional signatures in *SENP6* loss DLBCL patients, we performed genome-wide differential gene expression analysis between samples harboring a 6q:DEL or 6q14.1:DEL lesion, respectively, and wild-type samples using the provided microarray data (*n* = 137) and the *limma* R package^47^. Moderated *t*-statistics per probe were collapsed per gene using their weighted mean with the absolute *t*-statistic per probe as weights. GSEA was carried out with the resulting differential gene expression signature using the *fgsea* R package. Gene sets were retrieved from the MSigDb v7.3^54,55^.

### Statistical analysis

Statistical analyzes were performed using GraphPad Prism (GraphPad Software, La Jolla, CA, USA). The error bars shown in the figures represent the standard deviation (SD), unless specified otherwise. In each experiment, the statistical tests used are indicated in the figure legends.

### Reporting summary

Further information on research design is available in the Nature Research Reporting Summary linked to this article.

## Supplementary information

Supplementary Information

Description of Additional Supplementary Files

Supplementary Data 1

Supplementary Data 2

Supplementary Data 3

Supplementary Data 4

Supplementary Data 5

Supplementary Data 6

Reporting summary

## Data Availability

The WGS data generated in this study have been deposited at EBI European Nucleotide Archive under accession PRJEB44263. The transcriptome data of OCI-Ly1 control and *SENP6*^*KD*^ cells generated in this study have been deposited in the GEO database under accession code GSE180052. The mass spectrometry proteomics data generated in this study have been deposited in the ProteomeXchange Consortium via the PRIDE partner repository^62^ with the dataset identifier PXD027355. The ChIP and transcriptome data of SU-DHL-5 EV and SENP6 cells generated in this study have been deposited in the GEO database under accession code GSE141913. The data generated in this study are provided in the Source Data file. Source data are provided with this paper.

## Source data

Source Data
