# Supplementary material for: Genetic alterations of the SUMO isopeptidase SENP6 drive lymphomagenesis and genetic instability in diffuse large B-cell lymphoma
Source: Nat Commun. 2022 Jan 12;13:281. doi: 10.1038/s41467-021-27704-8 (PMC8755833; doi:10.1038/s41467-021-27704-8)
Supplement: Supplementary file 1 — Supplementary Information [file 41467_2021_27704_MOESM1_ESM.pdf]

Genetic alterations of the SUMO isopeptidase SENP6 drive lymphomagenesis and genetic instability in diffuse large B-cell lymphoma

Schick et al.

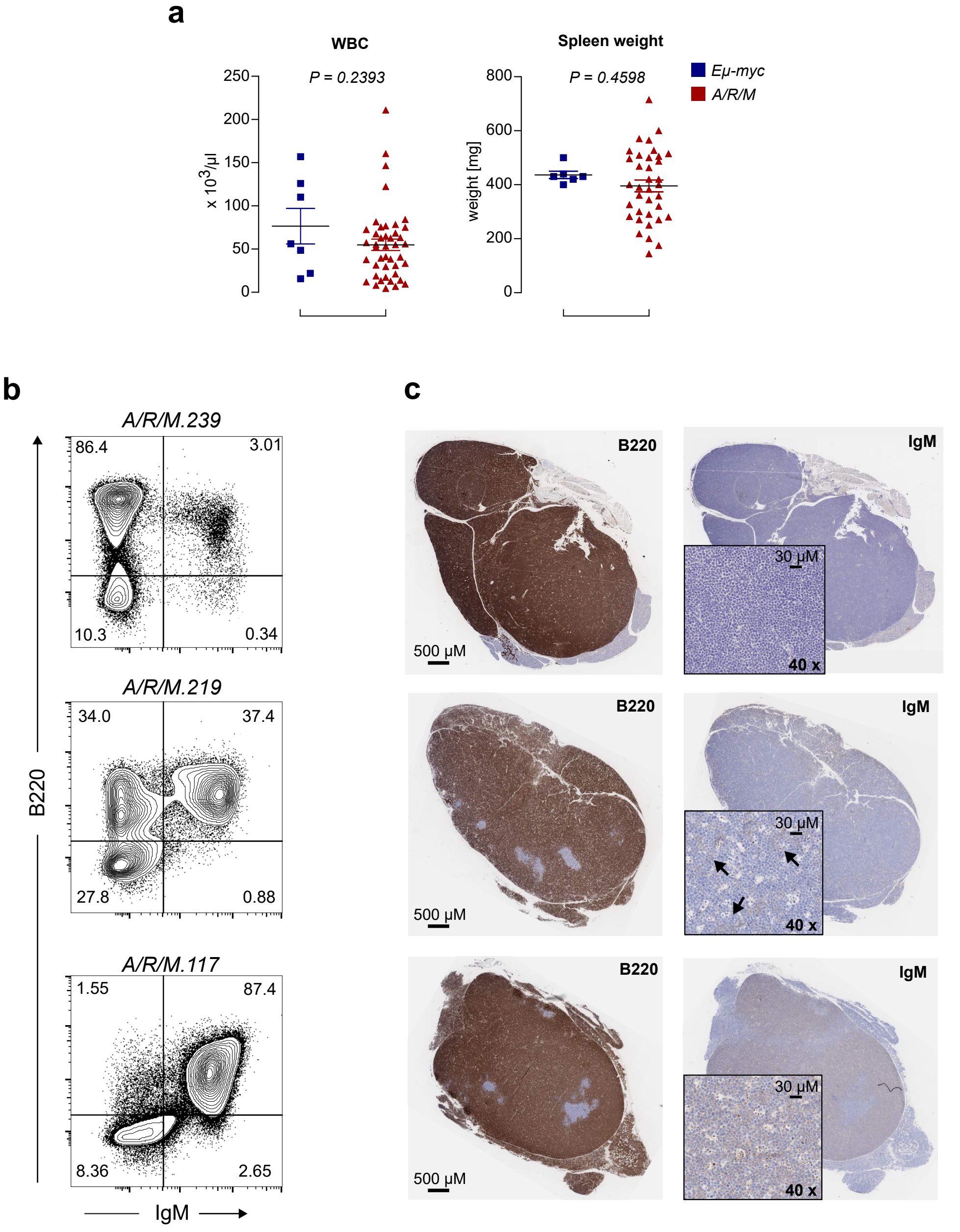

Supplementary Figure 1. **B -cell lymphoma phenotype of *A/R/M* mice.**

(a) Analysis of white blood cell counts (WBC) and spleen weight of *A/R/M* mice (n=36 for spleen weight, n=43 for WBC) and *Eµ-myc* control mice (n=6 for spleen weight, n=7 for WBC). *P-value* was determined by unpaired t-test (two-tailed) . Data are presented as mean +/- SD.

(b) Single cell suspensions of tumors arising in *A/R/M* mice stained with specific antibodies against the B-cell marker B220 and IgM. Shown is only the population of CD45+ viable cells.

(c) Histological and immunohistochemical analysis of three representative *A/R/M* lymphomas. The analysis was performed for three mice of each group with similar results.

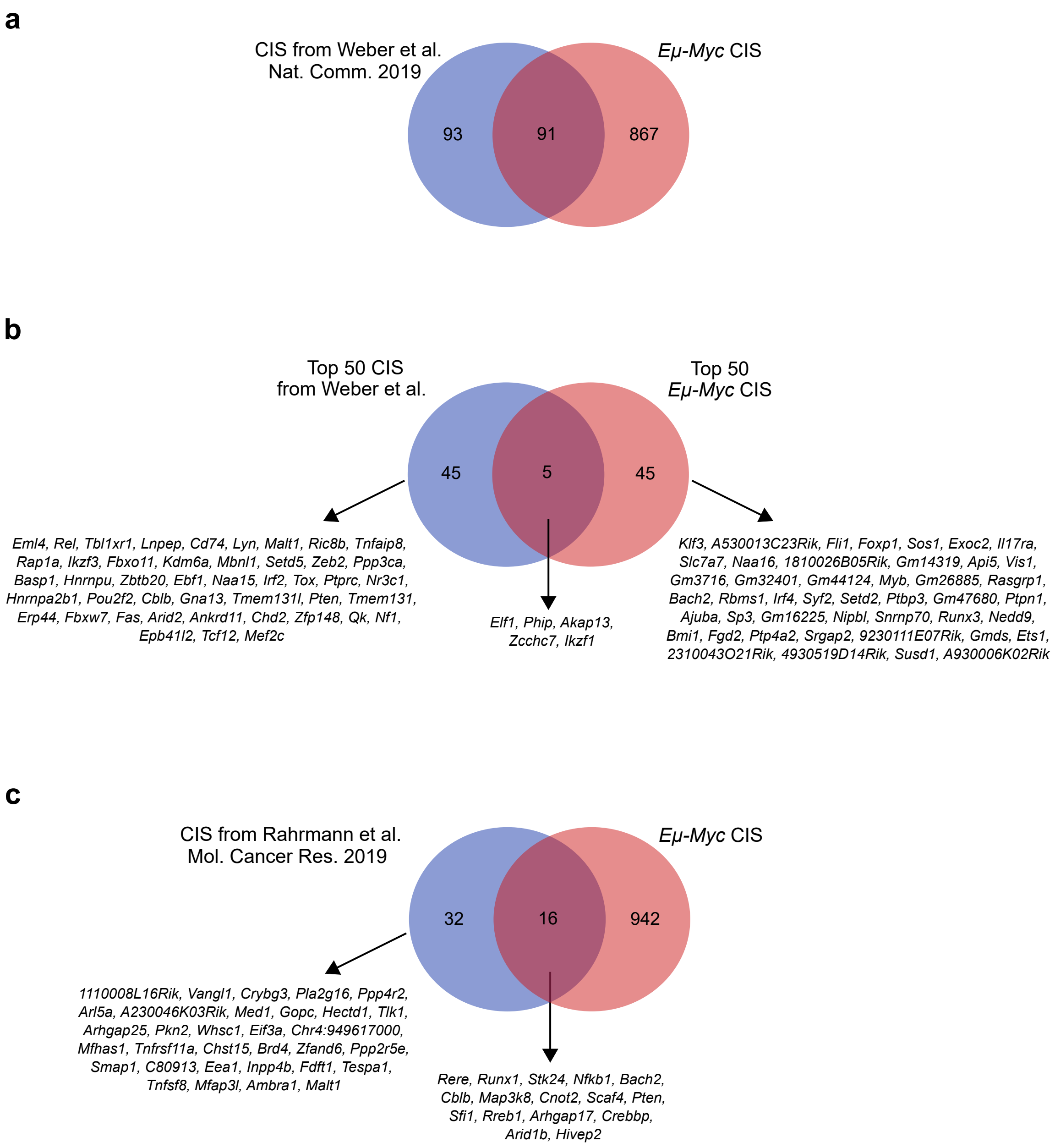

Supplementary Figure 2. Comparison of *Eµ-Myc PB* screening to related studies.

(a) Venn diagram showing the overlap between CISs in *A/R/M* lymphomas and KERNEL CISs described in Weber et al.[1] .

(b) Venn diagram showing the overlap between top 50 CISs in *A/R/M* lymphomas and top 50 KERNEL CISs described in Weber et al.[1]. CISs were sorted by number of transposon insertions.

(c) Venn diagram showing the overlap between CISs in *A/R/M* lymphomas and CISs described in Rahrmann et al.[2] .

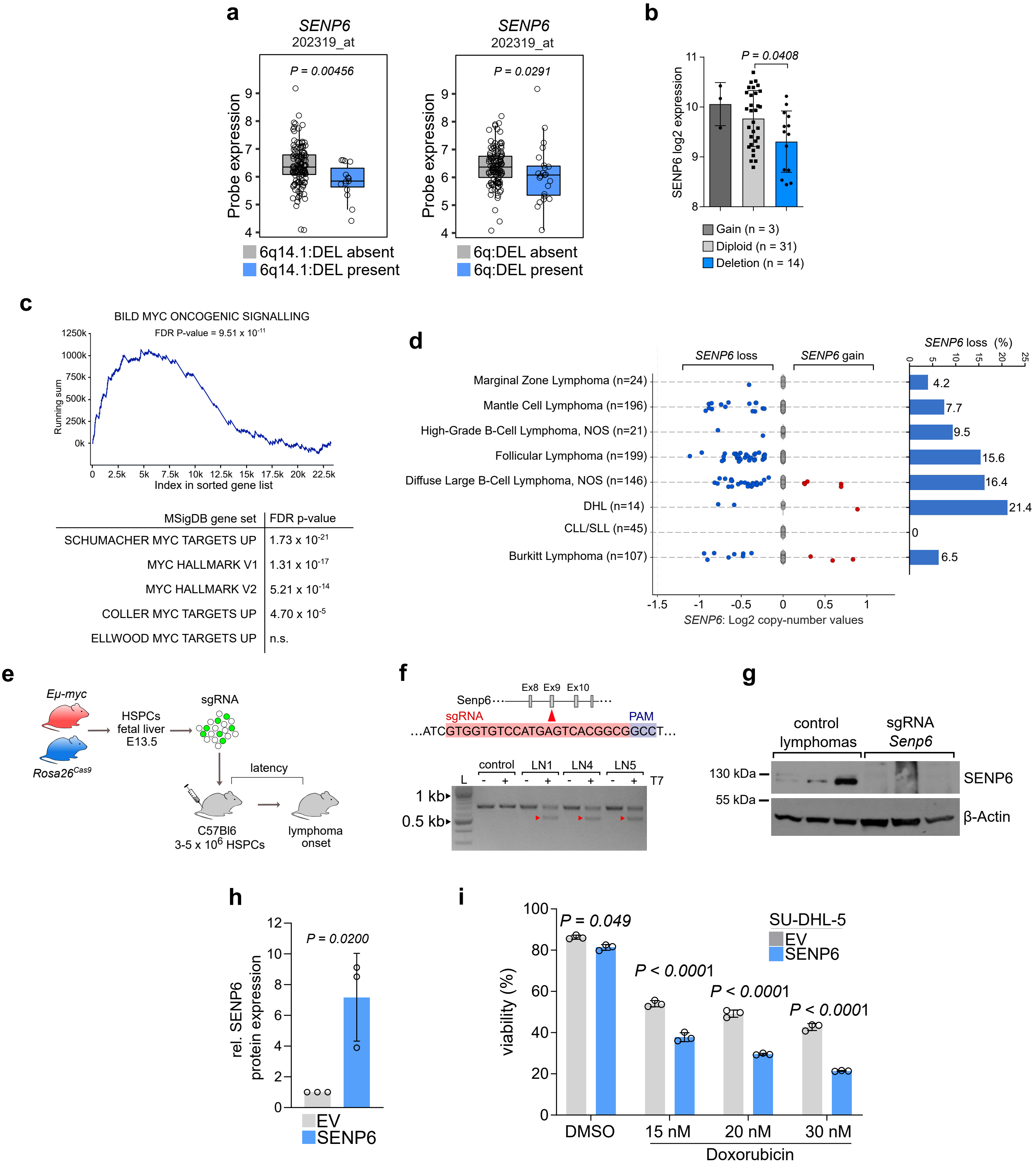
a
SENP6
202319_at
P = 0.00456
P = 0.0291
Probe expression
6q14.1:DEL absent
6q14.1:DEL present
6q:DEL absent
6q:DEL present
b
P = 0.0408
SENP6 log2 expression
Gain (n = 3)
Diploid (n = 31)
Deletion (n = 14)
c
BILD MYC ONCOGENIC SIGNALLING
FDR P-value = 9.51 x 10^-11
Running sum
Index in sorted gene list
MSigDB gene set | FDR p-value
SCHUMACHER MYC TARGETS UP | 1.73 x 10^-21
MYC HALLMARK V1 | 1.31 x 10^-17
MYC HALLMARK V2 | 5.21 x 10^-14
COLLER MYC TARGETS UP | 4.70 x 10^-5
ELLWOOD MYC TARGETS UP | n.s.
d
SENP6 loss
SENP6 gain
SENP6 loss (%)
Marginal Zone Lymphoma (n=24) 4.2
Mantle Cell Lymphoma (n=196) 7.7
High-Grade B-Cell Lymphoma, NOS (n=21) 9.5
Follicular Lymphoma (n=199) 15.6
Diffuse Large B-Cell Lymphoma, NOS (n=146) 16.4
DHL (n=14) 21.4
CLL/SLL (n=45) 0
Burkitt Lymphoma (n=107) 6.5
SENP6: Log2 copy-number values
e
Eμ-myc
Rosa26Cas9
HSPCs fetal liver E13.5
sgRNA
latency
C57Bl6
3-5 x 10^6 HSPCs
lymphoma onset
f
Senp6
Ex8 Ex9 Ex10
sgRNA
PAM
...ATCGTGGTGTCCATGAGTCACGGCGGCCT...
control LN1 LN4 LN5
L
T7
1 kb
0.5 kb
g
control lymphomas
sgRNA Senp6
130 kDa
55 kDa
SENP6
β-Actin
h
P = 0.0200
rel. SENP6 protein expression
EV
SENP6
i
P = 0.049
P < 0.0001
P < 0.0001
P < 0.0001
SU-DHL-5
EV
SENP6
viability (%)
DMSO
15 nM
20 nM
30 nM
Doxorubicin

Supplementary Figure 3. *SENP6* alterations in B-cell lymphomas.

(a) *SENP6* mRNA levels in DLBCL groups according to their *SENP6* copy number status. 6q14.1:DEL absent, n=122; 6q14.1:DEL present, n=15; 6q:DEL absent, n=114; 6q:DEL present, n=23. The center line of the box plot is the median. The box extends from the 25th to 75th percentiles. Whisker length is from minimum to maximum. *P-value* determined by Wilcoxon Rank Sum Test (two -sided).

(b) Expression analysis of *SENP6* mRNA in the human TCGA DLBCL dataset (n=48) derived from https://www.cbioportal.org/. Groups were classified according to their *SENP6* copy number status. Data are presented as mean +/- SD. *P-value* determined by one-way ANOVA; Tukey`s post hoc test.

(c) GSEA of expression data derived from primary DLBCL patient samples described in Figure S3b. Groups were classified according to their *SENP6* copy number status.

(d) *SENP6* copy number status in different mature B-cell lymphomas derived from https://www.cbioportal.org/.

(e) Experimental transduction-transplantation strategy for *in vivo* validation of tumor suppressor genes, here *Senp6*. HSPCs, hematopoietic stem and progenitor cells.

(f) *In vitro* T7 nuclease assay showing cutting efficacies of the Senp6-sgRNA in lymphomas from *in vivo* validation experiment. The experiment was repeated two times with similar results on lymphomas derived from three biologically independent mice.

(g) Western blot analysis of *Senp6-sgRNA* lymphomas (n=3) and control lymphomas (n=3) with the indicated antibodies.

(h) Quantification of the SENP6 Western blots from Figure 3b. Protein expression of SENP6 in EV cells was arbitrarily set to 1. Data are presented as mean +/- SD of n=3 independent experiments. *P-value* determined by unpaired t-test (two -tailed).

(i) Viability of indicated cell lines after 48h treatment with the indicated concentrations of doxorubicin. Viability determined by propidium iodide staining and flow cytometry measurement. Data are presented as mean +/- SD of n=3 independent experiments. *P-value* determined by two-way ANOVA; Bonferroni's multiple comparisons test.

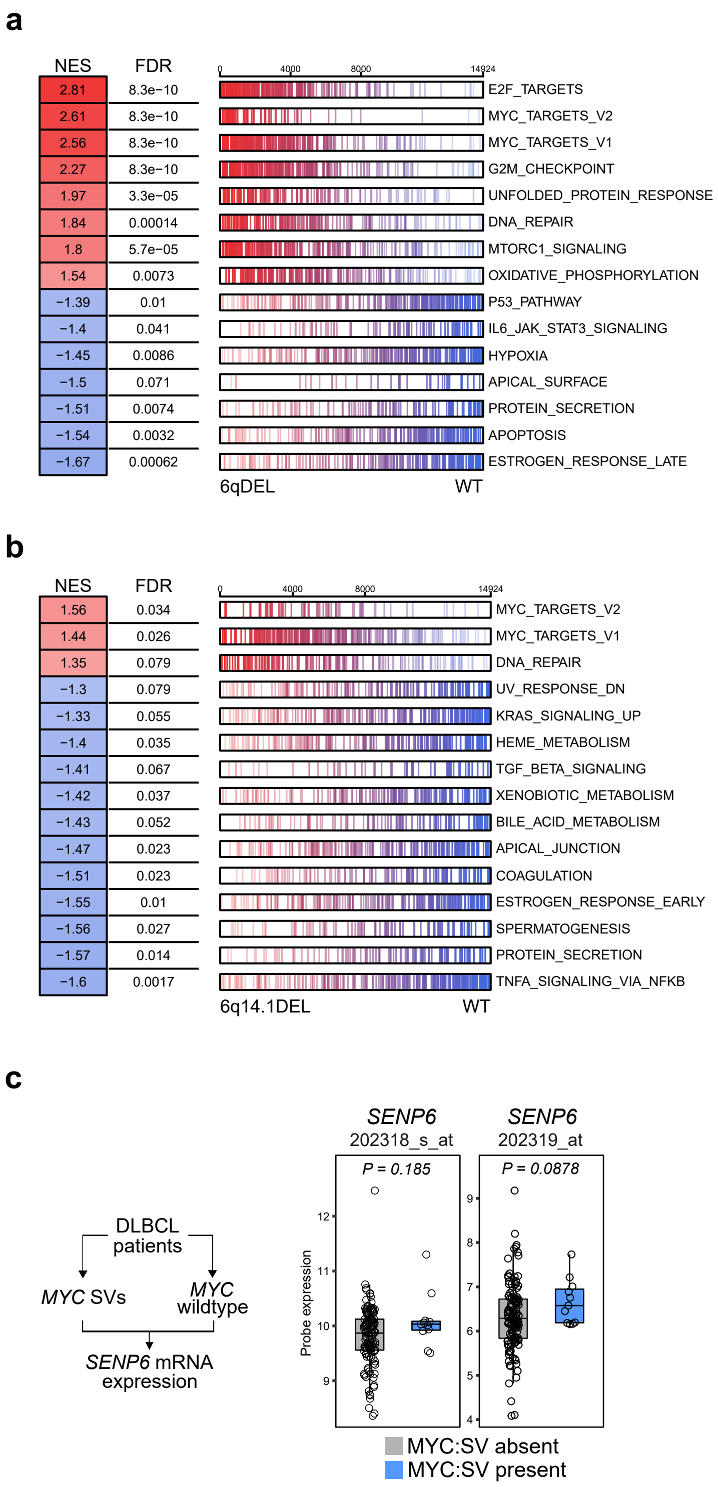

Supplementary Figure 4. MYC signaling in DLBCL patients harboring *SENP6* loss.

(a, b) Summary of GSEA of expression data derived from DLBCL patients harboring *SENP6* CNAs with the indicated gene sets. Assessed was the DLBCL patient dataset from Chapuy et al.[3].

(c) *SENP6* mRNA levels in DLBCL groups according to their *MYC* SV status. *MYC* SV absent, n=126; MYC SV present, n=11. Assessed was the DLBCL patient dataset from Chapuy et al.[3]. The center line of the box plot is the median. The box extends from the 25th to 75th percentiles. Whisker length is from minimum to maximum. *P-value* determined by Wilcoxon Rank Sum Test (two-sided).

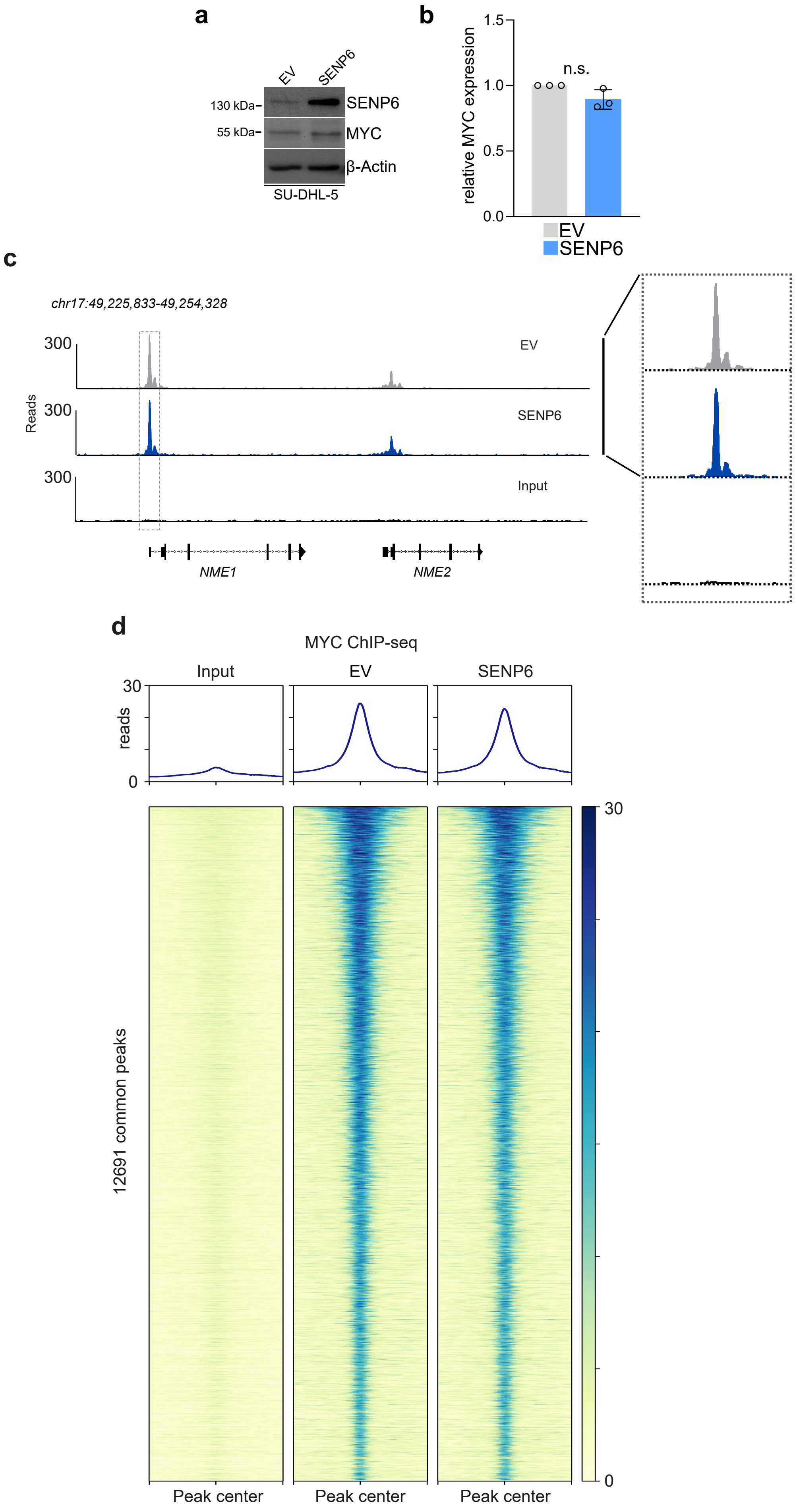

a
EV
SENP6
130 kDa
55 kDa
SENP6
MYC
β-Actin
SU-DHL-5
b
relative MYC expression
1.5
1.0
0.5
0.0
n.s.
EV
SENP6
c
chr17:49,225,833-49,254,328
300
300
300
Reads
EV
SENP6
Input
NME1
NME2
d
MYC ChIP-seq
Input
EV
SENP6
30
0
reads
12691 common peaks
30
0
Peak center
Peak center
Peak center

Supplementary Figure 5. MYC binding in DLBCL cell lines with defined SENP6 status.

(a) Immunoblot analysis of SU-DHL-5 EV and SENP6 cell lines for MYC and SENP6 expression. The experiment was repeated three times with similar results.

(b) Quantification of the MYC western blots from Figure S5a. MYC expression in SU -DHL-5 EV cells was arbitrarily set to 1. Data are presented as mean +/- SD of n=3 independent experiments. *P-value* determined by unpaired t-test (two -tailed).

(c) Genome browser picture of read-normalized MYC ChIPseq profiles from SU-DHL -5 cells with low SENP6 expression (EV, grey) or reconstituted for SENP6 expression (SENP6, blue) described in Figure S5a. Input is shown in black.

(d) Heat maps centered around the common MYC peaks showing z-score from binding of endogenous MYC in SU-DHL-5 EV and SU-DHL-5 SENP6 cell lines. Input is shown as control and intensity of color indicates binding strength.

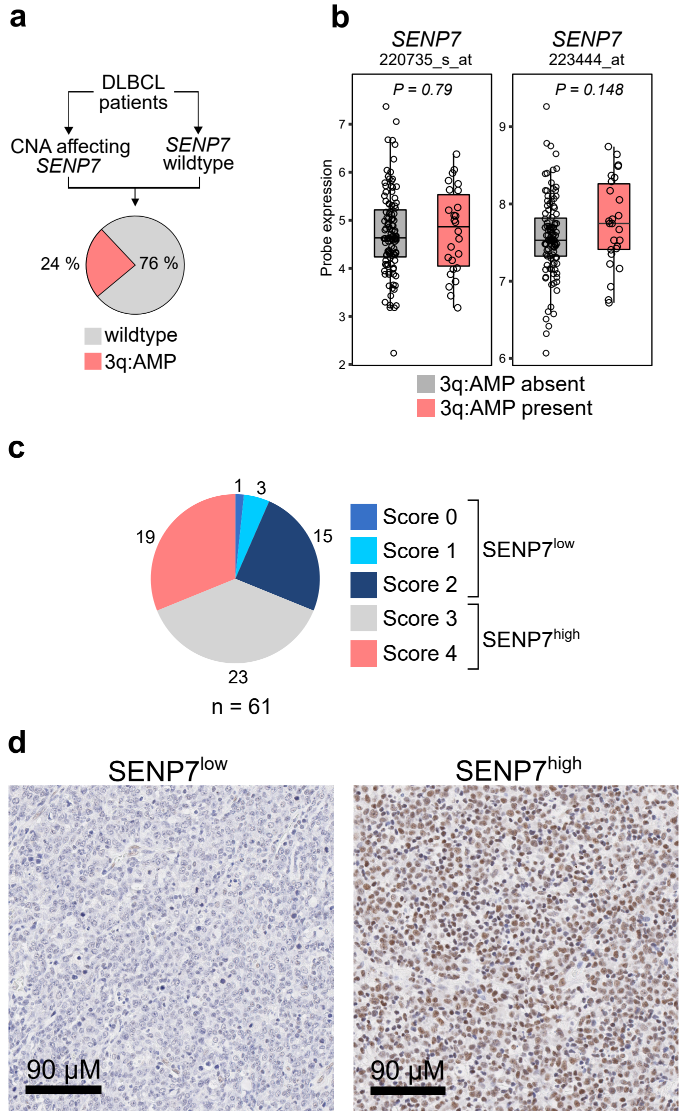

Supplementary Figure 6. SENP7 alterations in human DLBCL patients.

(a) Copy number alterations affecting *SENP7* in human DLBCL (n=304). W *ildtype*, n=231; 3q:AMP, n=73.

(b) *SENP7* mRNA levels in DLBCL groups according to their *SENP6* copy number status. 3q:AMP absent, n=137; 3q:AMP present, n=26. The center line of the box plot is the median. Assessed was the dataset from Chapuy et al.[3].The box extends from the 25th to 75th percentiles. Whisker length is from minimum to maximum. *P-value* determined by Wilcoxon Rank Sum Test (two -sided).

(c, d) SENP7 protein expression by immunohistochemistry in human DLBCL samples. The results were similar for all SENP7^{high} and SENP7^{low} patients.

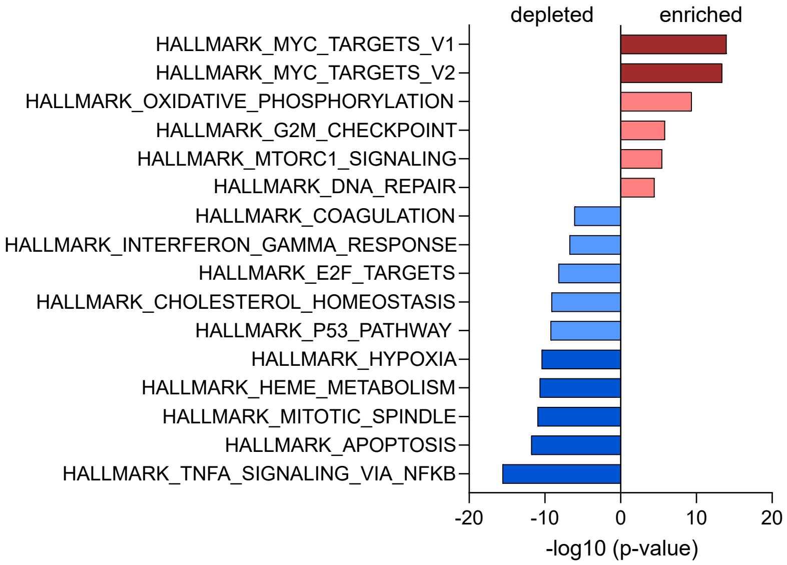

Supplementary Figure 7. Transcriptome profiling of SENP6-deficient DLBCL cells.

Summary of GSEA of expression data derived from transcriptome profiling of OCI -Ly1 control and OCI-Ly1 *SENP6*^{KD} cell lines with the indicated gene sets. *P-value* determined by Kolmogorov-Smirnov test.

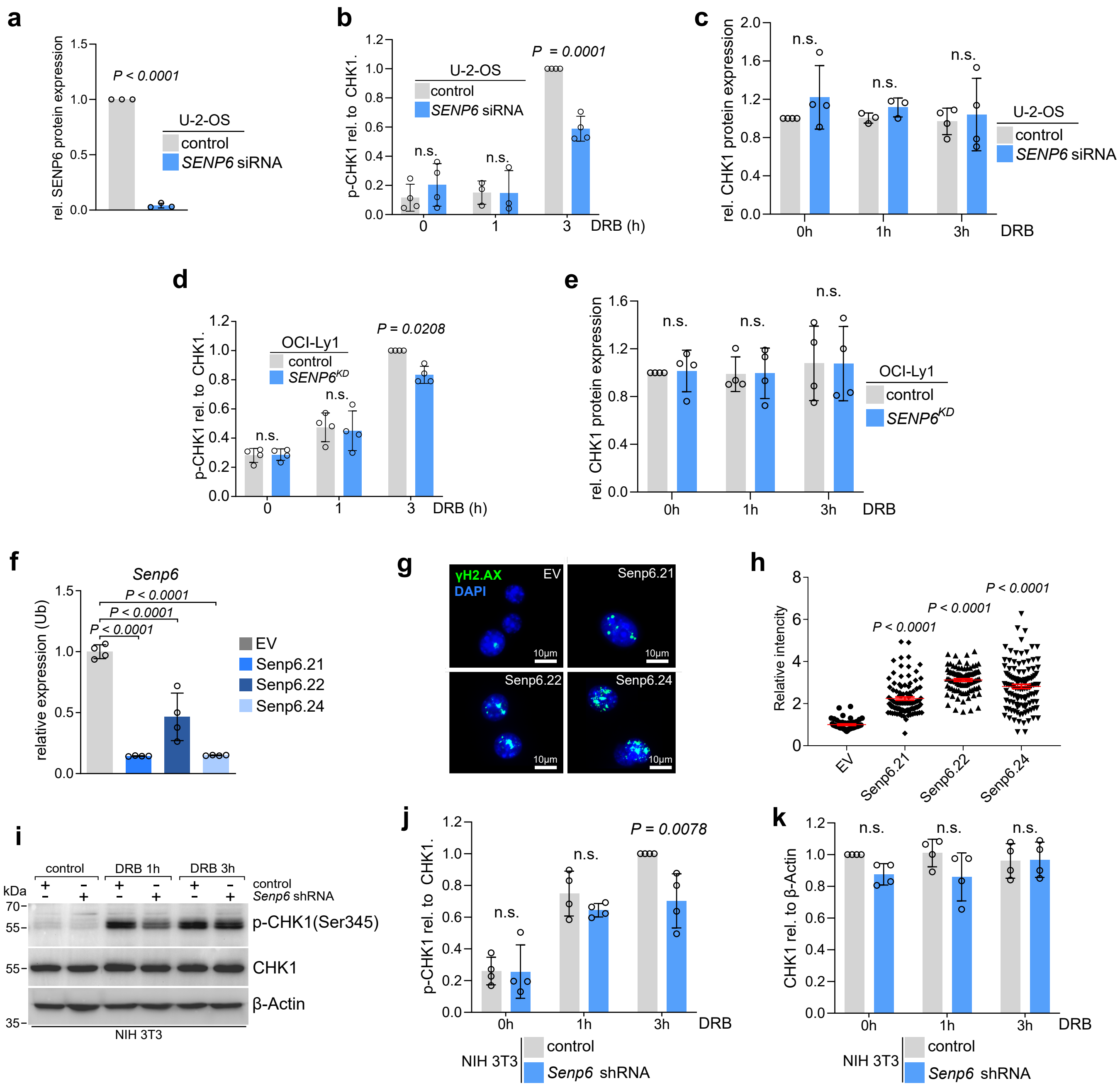

a
rel. SENP6 protein expression
P < 0.0001
U-2-OS
control
SENP6 siRNA
b
p-CHK1 rel. to CHK1.
U-2-OS
control
SENP6 siRNA
P = 0.0001
n.s.
n.s.
DRB (h)
c
rel. CHK1 protein expression
n.s.
n.s.
n.s.
U-2-OS
control
SENP6 siRNA
DRB
d
p-CHK1 rel. to CHK1.
OCI-Ly1
control
SENP6KD
P = 0.0208
n.s.
n.s.
DRB (h)
e
rel. CHK1 protein expression
n.s.
n.s.
n.s.
OCI-Ly1
control
SENP6KD
DRB
f
Senp6
relative expression (Ub)
P < 0.0001
P < 0.0001
P < 0.0001
EV
Senp6.21
Senp6.22
Senp6.24
g
γH2.AX
DAPI
EV
Senp6.21
Senp6.22
Senp6.24
10μm
h
Relative intencity
P < 0.0001
P < 0.0001
P < 0.0001
EV
Senp6.21
Senp6.22
Senp6.24
i
control
DRB 1h
DRB 3h
control
Senp6 shRNA
kDa
p-CHK1(Ser345)
CHK1
β-Actin
NIH 3T3
j
p-CHK1 rel. to CHK1.
P = 0.0078
n.s.
n.s.
DRB
NIH 3T3
control
Senp6 shRNA
k
CHK1 rel. to β-Actin
n.s.
n.s.
n.s.
DRB
NIH 3T3
control
Senp6 shRNA

Supplementary Figure 8. SENP6 loss is linked to deficient DDR.

(a) Quantification of the SENP6 western blots in U-2-OS cells transfected with control or *SENP6* siRNA. Protein expression of SENP6 in control siRNA transfected cells was arbitrarily set to 1. Data are presented as mean +/- SD of n=3 independent experiments. *P-value* determined by unpaired t-test (two-tailed).

(b) Quantification of the p-CHK1 western blots from Figure 5d. Protein expression of p -CHK1 was normalized to CHK1 protein expression. p-CHK1 expression in U -2-OS cells transfected with control siRNA at 3h doxorubicin treatment was arbitrarily set to 1. Data are presented as mean +/- SD of n=3 (1h) and n=4 (0h and 3h). Each dot represents a biological replicate. *P-value* determined by two -way ANOVA; Bonferroni's multiple comparisons test.

(c) Quantification of the CHK1 western blots from Figure 5d. Protein expression of CHK1 was normalized to loading control. CHK1 expression in U-2-OS cells transfected with control siRNA at 0h doxorubicin treatment was arbitrarily set to 1. Data are presented as mean +/- SD of n=3 (1h) and n=4 (0h and 3h) independent experiments. Each dot represents a biological replicate. *P-value* determined by two-way ANOVA; Bonferroni's multiple comparisons test.

(d) Quantification of the p-CHK1 western blots from Figure 5e. Protein expression of p -CHK1 was normalized to CHK1 protein expression. p-CHK1 expression in OCI -Ly1 control cells at 3h doxorubicin treatment was arbitrarily set to 1. Data are presented as mean +/- SD of n=4 independent experiments. *P-value* determined by two-way ANOVA; Bonferroni's multiple comparisons test.

(e) Quantification of the CHK1 western blots from Figure 5e. Protein expression of CHK1 was normalized to loading control. CHK1 expression in OCI-Ly1 control cells at 0h doxorubicin treatment was arbitrarily set to 1. Data are presented as mean +/- SD of n=4 independent experiments. *P-value* determined by two-way ANOVA; Bonferroni's multiple comparisons test.

(f) *Senp6* mRNA expression in NIH-3T3 cells after transduction with either empty vector or three *Senp6* shRNA constructs. *Senp6* expression was normalized to *Ubiquitin* . EV, empty vector. Data are presented as mean +/- SD of n=4 independent experiments. *P-value* determined by two-way ANOVA; Bonferr oni's multiple comparisons test.

(g) Immunofluorescence staining of γ.H2.AX expression of cells described in Figure S 8f. The experiment was repeated three times with similar results.

(h) Quantification of immunofluorescence staining described in Figure S8f. Data are presented as mean +/- SEM. In total n=51 (EV), n=103 (Senp6.21), n=92 (Senp6.22) and n=113 (Senp6.24) cells were quantified. *P-value* determined by one -way ANOVA; Dunnett's multiple comparisons test.

(i) Immunoblot analysis of indicated proteins in NIH-3T3 cell line transduced with empty vector (EV) or *Senp6* shRNA #21 after doxorubicin (DRB) treatment for the indicated times. The experiment was repeated three times with similar results.

(j) Quantification of the p-CHK1 western blots from Figure S8i. Protein expression of p-CHK1 was normalized to CHK1 protein expression. p-CHK1 expression in NIH-3T3 control cells at 3h doxorubicin treatment was arbitrarily set to 1. Data are presented as mean +/- SD of n=4 independent experiments. *P-value* determined by two-way ANOVA; Bonferroni`s multiple comparisons test.

(k) Quantification of the CHK1 western blots from Figure S8i. Protein expression of CHK1 was normalized to loading control. CHK1 expression in NIH-3T3 cont rol cells at 0h doxorubicin treatment was arbitrarily set to 1. Data are presented as mean +/- SD of n=4 independent experiments. *P-value* determined by two-way ANOVA; Bonferroni`s multiple comparisons test.

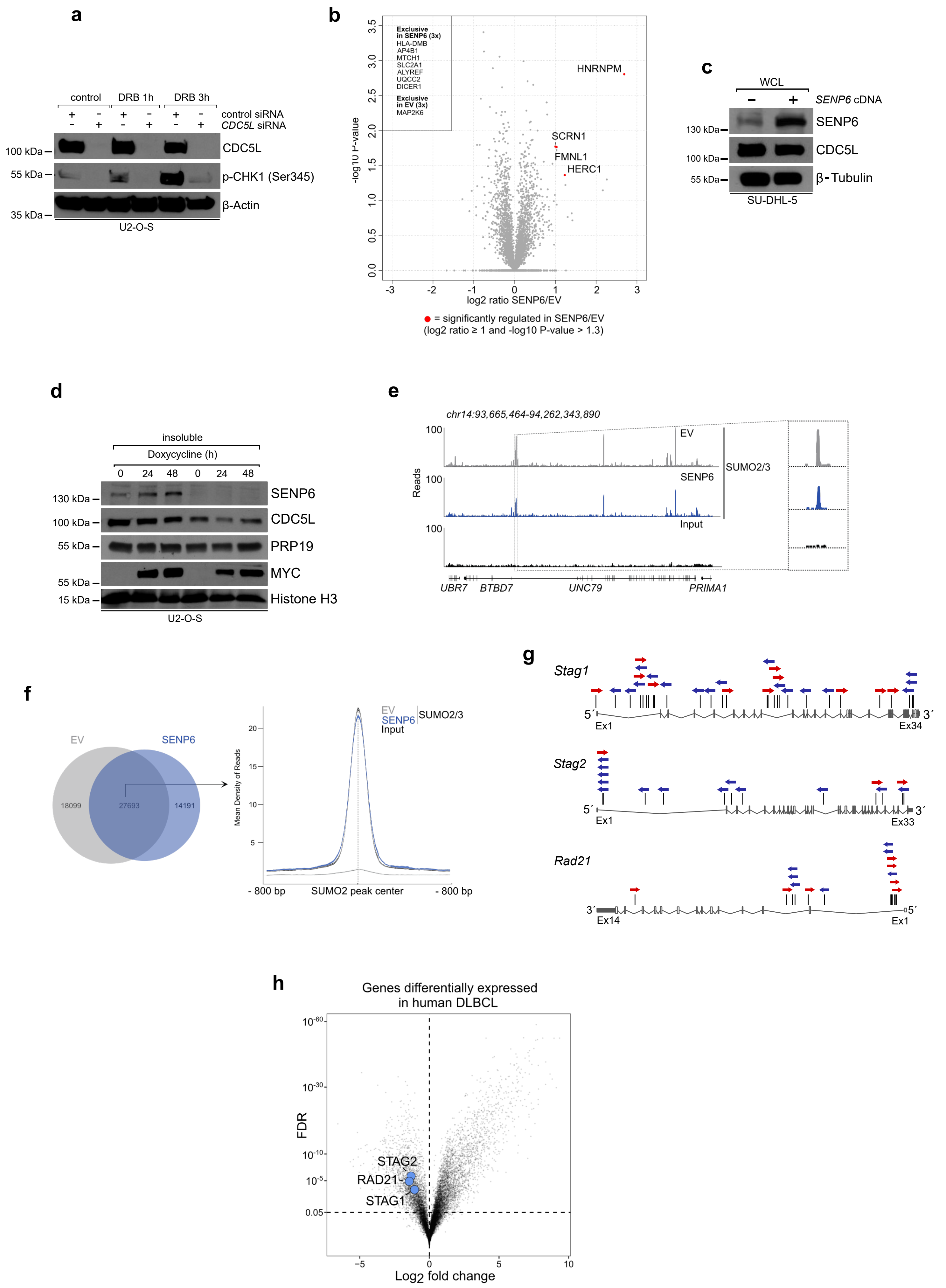

a
control
DRB 1h
DRB 3h
control siRNA
CDC5L siRNA
100 kDa
55 kDa
35 kDa
CDC5L
p-CHK1 (Ser345)
β-Actin
U2-O-S
b
Exclusive in SENP6 (3x)
HLA-DMB
AP4B1
MTCH1
SLC2A1
ALYREF
UQCC2
DICER1
Exclusive in EV (3x)
MAP2K6
HNRNPM
SCRN1
FMNL1
HERC1
-log10 P-value
log2 ratio SENP6/EV
= significantly regulated in SENP6/EV
(log2 ratio ≥ 1 and -log10 P-value > 1.3)
c
WCL
SENP6 cDNA
130 kDa
100 kDa
55 kDa
SENP6
CDC5L
β-Tubulin
SU-DHL-5
d
insoluble
Doxycycline (h)
0 24 48 0 24 48
130 kDa
100 kDa
55 kDa
55 kDa
15 kDa
SENP6
CDC5L
PRP19
MYC
Histone H3
U2-O-S
e
chr14:93,665,464-94,262,343,890
Reads
EV
SENP6
Input
SUMO2/3
UBR7
BTBD7
UNC79
PRIMA1
f
EV
SENP6
18099
27693
14191
Mean Density of Reads
SUMO2/3
Input
- 800 bp
SUMO2 peak center
- 800 bp
g
Stag1
Ex1
Ex34
Stag2
Ex1
Ex33
Rad21
Ex14
Ex1
h
Genes differentially expressed in human DLBCL
FDR
STAG2
RAD21
STAG1
Log2 fold change

Supplementary Figure 9. SENP6 loss and chromatin extraction.

(a) Immunoblot analysis of U-2-OS cells after transfection with specific CDC5L siRNA or control siRNA and doxorubicin (DRB) treatment for the indicated times. The experiment was repeated three times with similar results.

(b) Volcano plot summarizing the results of quantitative MS analysis from the SU-DHL -5 DLBCL cell line after reconstitution of SENP6 expression described in Figure 3b. Proteins considered as significantly enriched are color-coded (cut-offs are indicated in the figure). The experiment was performed in triplicates.

(c) Immunoblot analysis of whole cell lysates (WCL) of the SU-DHL-5 DLBCL cell line described in Figure 3b. The experiment was repeated three times with similar results.

(d) Immunoblot analysis of insoluble fraction of U-2-OS cells after transfection with specific SENP6 siRNA or control siRNA and induction of MYC for the indicated time points (in hours). The experiment was repeated three times with similar results.

(e) Genome browser picture of read normalized SUMO2/3 ChIPseq profiles from SU -DHL-5 cells with low SENP6 expression (EV, grey) or reconstituted for SENP6 expression (SENP6, blue). Input is shown in black.

(f) Venn diagram (left) showing overlap of SUMO2/3 peaks in SU-DHL-5 cells with low SENP6 expression (EV, grey) or reconstituted for SENP6 expression (SENP6, blue). Density plot centered at 27693 common SUMO2/3 peaks (right). Input is shown in black. Shadow of the curves indicates the SEM of the density.

(g) Transposon insertion pattern in *Rad21*, *Stag1* and *Stag2*. Only the dominant insertion per tumor is shown.

(h) Volcano plot of differentially expressed genes in human DLBCL (n=73) in comparison to germinal center B-cells (n=10). Assessed was GSE12195. Components of the cohesin complex which have been identified in the *PB* screen are highlighted.

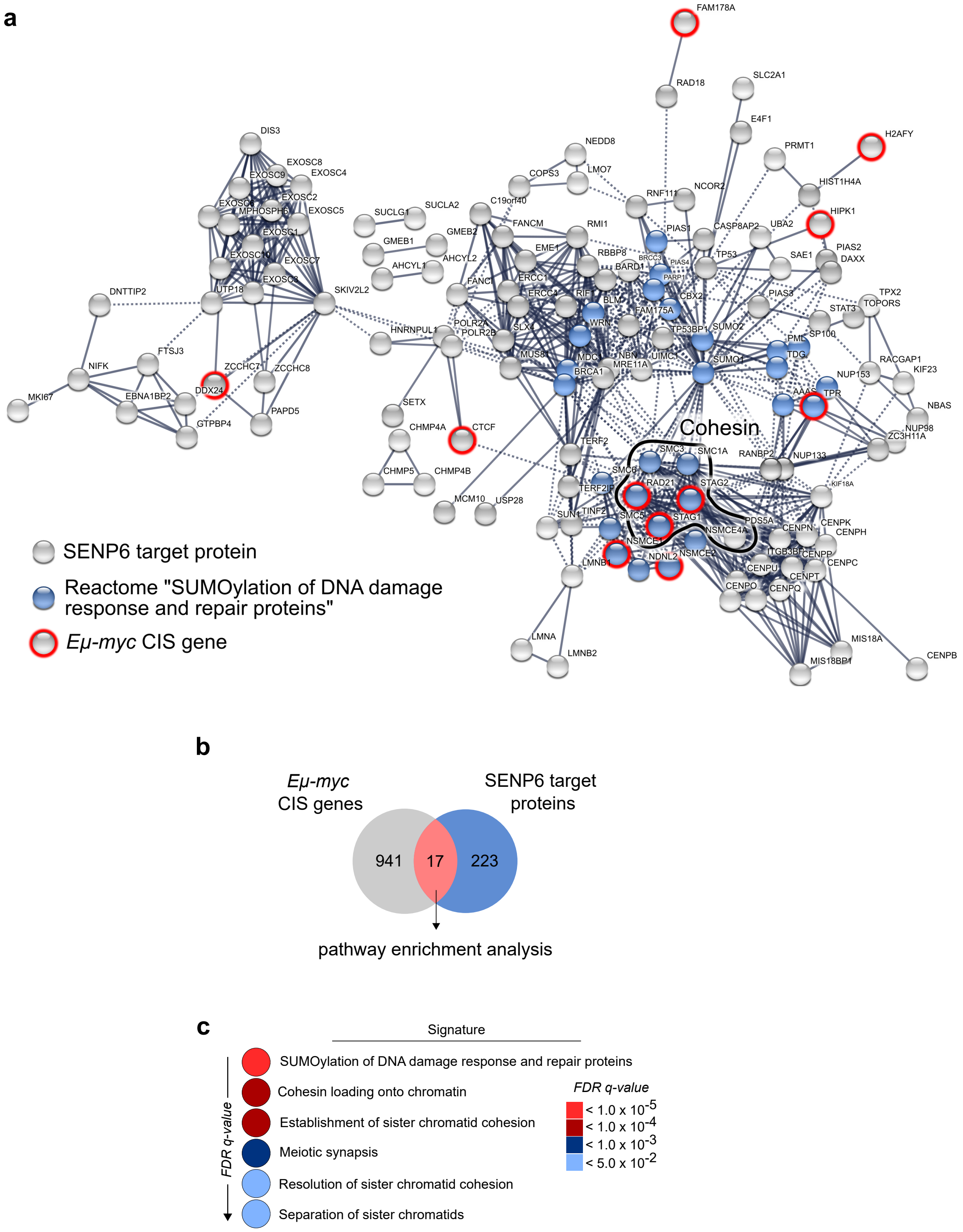

Supplementary Figure 10. Network analysis of SENP6 targets.

(a) STRING network analysis depicting the interconnection of SENP6 targets and the resulting clusters. For STRING analysis both recently described SENP6 targetomes[4,5] were combined. The highest confidence filter (0.9) was applied and only connected proteins are visualized.

(b) Venn diagram showing the overlap between CIS genes derived from the *Eμ-myc* transposon mutagenesis screen and SENP6 target proteins[4,5].

(c) Overlapping candidates from Figure 6d were analyzed using the Reactome database. Color-coded *FDR q-value* is shown for all significantly enriched pathways.

a

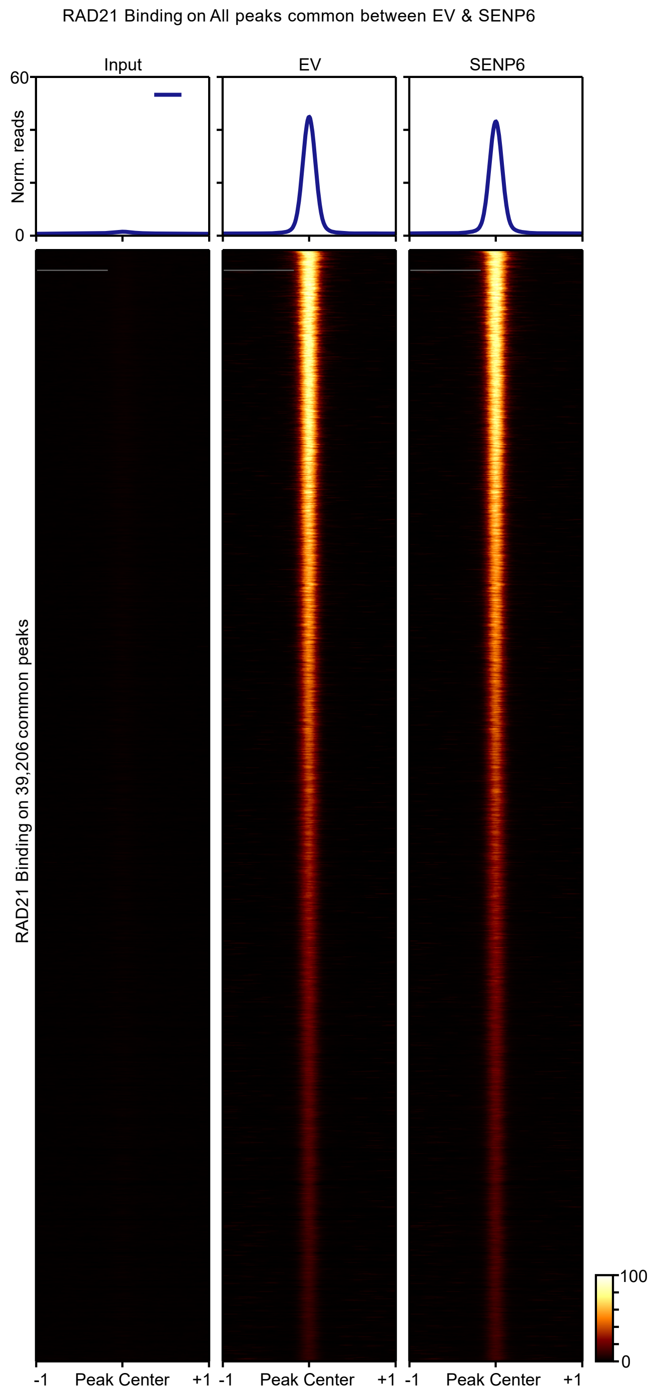
RAD21 Binding on All peaks common between EV & SENP6
Input
EV
SENP6
60
0
Norm. reads
RAD21 Binding on 39,206 common peaks
100
0
-1
Peak Center
+1

b

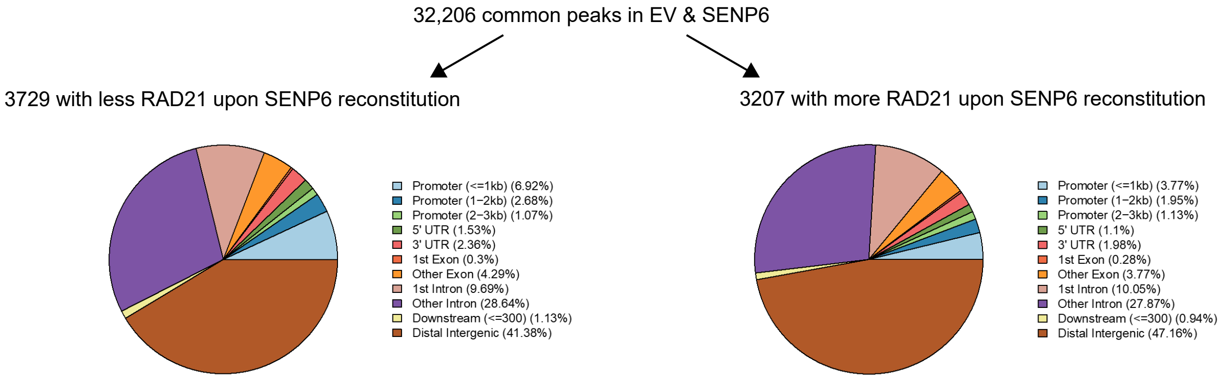
32,206 common peaks in EV & SENP6
3729 with less RAD21 upon SENP6 reconstitution
3207 with more RAD21 upon SENP6 reconstitution
Promoter (<=1kb) (6.92%)
Promoter (1−2kb) (2.68%)
Promoter (2−3kb) (1.07%)
5' UTR (1.53%)
3' UTR (2.36%)
1st Exon (0.3%)
Other Exon (4.29%)
1st Intron (9.69%)
Other Intron (28.64%)
Downstream (<=300) (1.13%)
Distal Intergenic (41.38%)
Promoter (<=1kb) (3.77%)
Promoter (1−2kb) (1.95%)
Promoter (2−3kb) (1.13%)
5' UTR (1.1%)
3' UTR (1.98%)
1st Exon (0.28%)
Other Exon (3.77%)
1st Intron (10.05%)
Other Intron (27.87%)
Downstream (<=300) (0.94%)
Distal Intergenic (47.16%)

Supplementary Figure 11. RAD21 binding in human DLBCL cells with defined SENP6 status.

(a) Heat maps showing z-score from for binding of endogenous RAD21 centered around common peaks in SU-DHL-5 EV and SU-DHL-5 SENP6 cell lines. Input is shown as control and intensity of color indicates binding strength.

(b) Pie-chart indicating the genomic location annotation of the selected common RAD21 peaks from (a), which are less bound (left) or stronger bound (right) by RAD21 upon SENP6 reconstitution. Downstream indicates 300 bp region after TES.

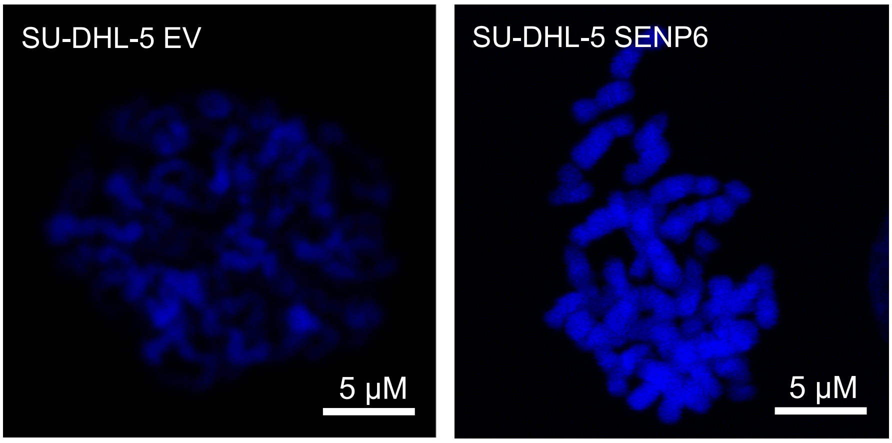

Supplementary Figure 12. Metaphase spreads of human DLBCL cell lines with defined SENP6 status.
Representative images of metaphase spreads from EV and SENP6 SU-DHL-5 cell lines described in Figure 3b.

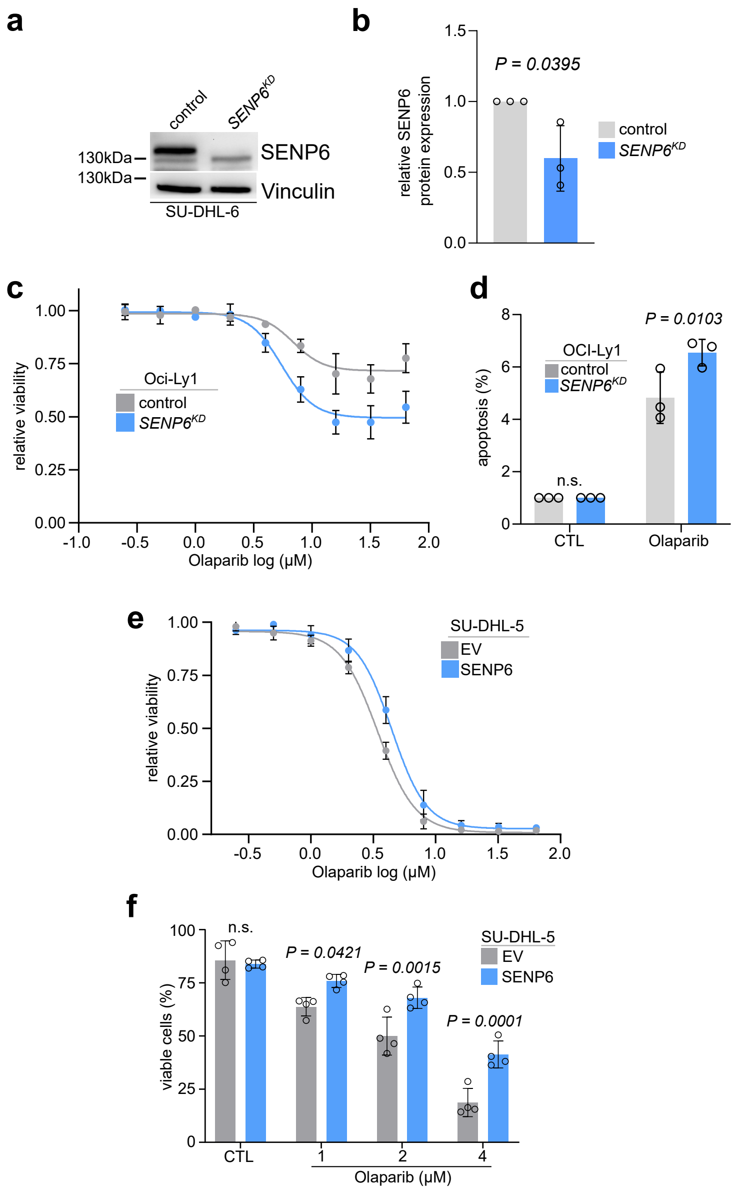

Supplementary Figure 13. SENP6 loss drives synthetic lethality to PARPi.

(a) Immunoblot analysis of human SU-DHL-6 DLBCL cells after CRISPR/Cas9-mediated depletion of SENP6. The experiment was repeated three times with similar results.

(b) Quantification of the SENP6 western blots in SU-DHL-6 control and *SENP6*^{KD} cells. Protein expression of SENP6 in control cells was arbitrarily set to 1. Data are presented as mean +/- SD of n=3 independent experiments. *P-value* determined by unpaired t-test (two-tailed).

(c) Olaparib dose response curves of *SENP6*^{KD} and control OCI-Ly1 cells. Cells were treated for 72h and viability was determined by PI staining and flow cytometry measurement. Data are presented as mean +/- SD of n=4 independent experiments.

(d) Quantification of flow cytometry results for PI and Annexin V staining of *SENP6* ^{KD} and control OCI-Ly1 cells after olaparib or control treatment for 72h. Apoptosis rate in control treated cells was arbitrarily set to 1. *P-value* determined by two-way ANOVA; Bonferroni`s multiple comparisons test. Data are presented as mean +/- SD of n=3 independent experiments.

(e) Olaparib dose response curves for human SU-DHL-5 SENP6 cells and SU-DHL -5 EV control cell line. Cells were treated for 72h and viability was determined by DAPI staining and flow cytometry measurement. Data are presented as mean +/- SD of n=3 independent experiments.

(f) Viability of human SU-DHL-5 SENP6 cells in comparison to SU-DHL-5 EV control cells after 48h olaparib treatment with the indicated concentrations. Viability determined by DAPI staining and flow cytometry measurement. P-value determined by two-way ANOVA; Bonferroni's multiple comparisons test. Data are presented as mean +/- SD of n=4 independent experiments.

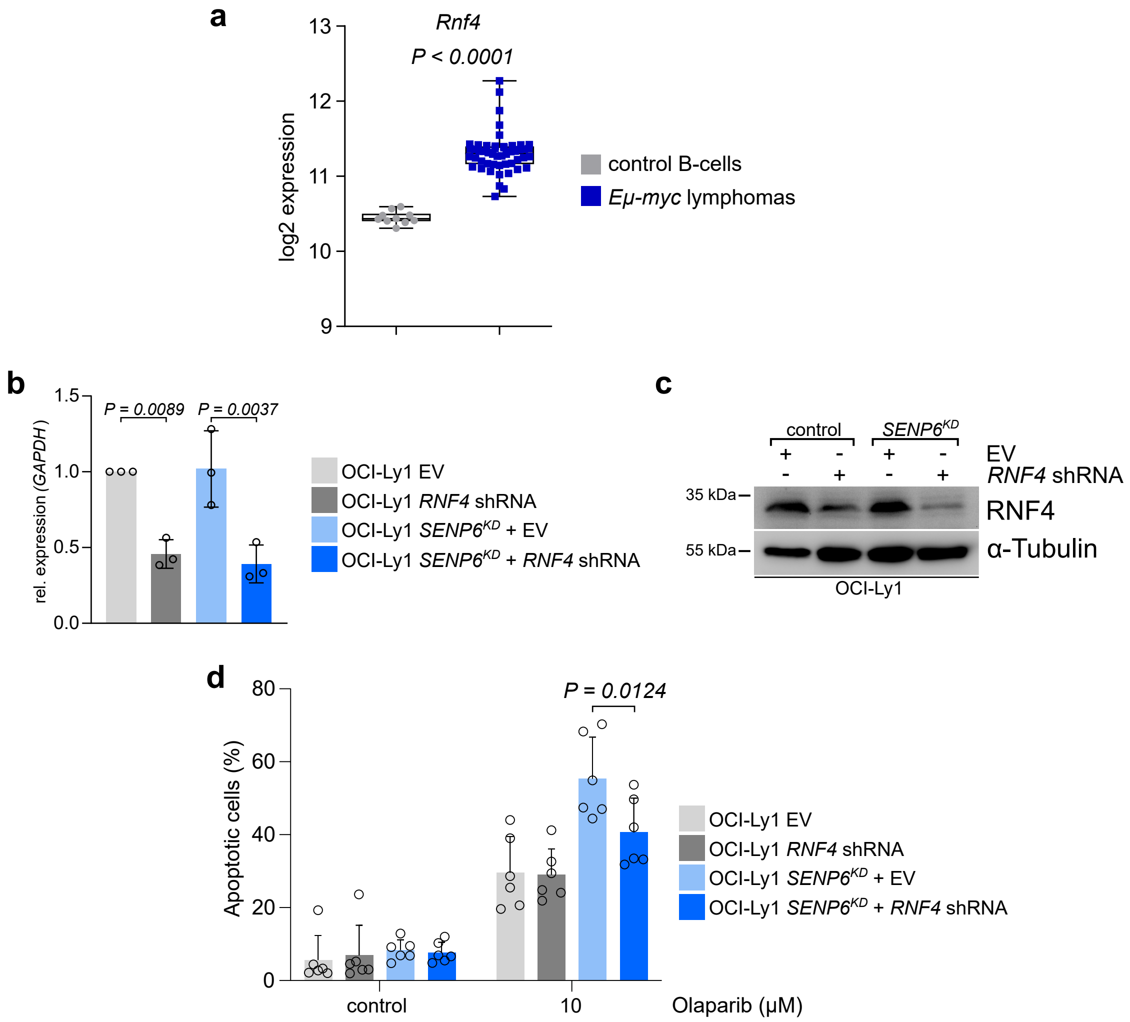

Supplementary Figure 14. SENP6 loss driven sensitivity to PARPi is mediated by RNF4.

(a) *Rnf4* expression in *Eμ-myc* lymphomas (n=50) in comparison to control B-cells (n=10). Assessed was GSE7897. The center line of the box plot is the median. The box extends from the 25th to 75th percentiles. Whisker length is from minimum to maximum. Data are presented as mean +/- SD. *P-value* determined by unpaired t-test (two-tailed).

(b) *RNF4* mRNA expression in OCI-Ly1 control and *SENP6*^{KD} cells after transduction with either empty vector or a *RNF4* shRNA construct. *RNF4* expres sion was normalized to *GAPDH*. EV, empty vector. Data are presented as mean +/- SD of n=3 independent experiments. *P-value* determined by one-way ANOVA; Tukey's post hoc test.

(c) Immunoblot analysis of OCI-Ly1 control and *SENP6*^{KD} cells after transduction with either empty vector (EV) or a *RNF4* shRNA construct. The experiment was repeated three times with similar results.

(d) Quantification of flow cytometry results for Annexin V staining in cell lines described in Figure S14b after olaparib treatment for 72h. Data are presented as mean +/- SD of n=6 independent experiments. *P-value* determined by two-way ANOVA; Tukey's post hoc test.

SUPPLEMENTARY REFERENCES

1. Weber, J., *et al.* PiggyBac transposon tools for recessive screening identify B-cell lymphoma drivers in mice. *Nat Commun* **10**, 1415 (2019).
2. Rahrmann, E.P., *et al.* Sleeping Beauty Screen Identifies RREB1 and Other Genetic Drivers in Human B-cell Lymphoma. *Mol Cancer Res* **17**, 567-582 (2019).
3. Chapuy, B., *et al.* Molecular subtypes of diffuse large B cell lymphoma are associated with distinct pathogenic mechanisms and outcomes. *Nat Med* **24**, 679-690 (2018).
4. Liebelt, F., *et al.* The poly-SUMO2/3 protease SENP6 enables assembly of the constitutive centromere-associated network by group deSUMOylation. *Nat Commun* **10**, 3987 (2019).
5. Wagner, K., *et al.* The SUMO Isopeptidase SENP6 Functions as a Rheostat of Chromatin Residency in Genome Maintenance and Chromosome Dynamics. *Cell Rep* **29**, 480-494 e485 (2019).
