# Supplementary material for: Genetic alterations of the SUMO isopeptidase SENP6 drive lymphomagenesis and genetic instability in diffuse large B-cell lymphoma
Source: Nat Commun. 2022 Jan 12;13:281. doi: 10.1038/s41467-021-27704-8 (PMC8755833; doi:10.1038/s41467-021-27704-8)
Supplement: Supplementary file 2 — Description of Additional Supplementary Files [file 41467_2021_27704_MOESM2_ESM.pdf]

Description of Additional Supplementary Files

Supplementary Data 1. Common insertion sites (CISs) resulting from CIMPL analysis. List of CIS identified by CIMPL (Common Insertion site Mapping Platform) analysis using non-redundant insertions.

Supplementary Data 2. List of genes from overlapping and exclusive regions of VENN diagrams depicted in Figure 1.

Supplementary Data 3. Reactome pathway analysis of *Eμ-Myc* CISs.

Supplementary Data 4. Proteome of chromatin fractionation in SU-DHL-5 EV and SU-DHL-5 SENP6 DLBCL cell lines. Samples were grouped into triplicates and a Student's t test was performed with a randomization of 500 and a s0 factor of 0.1.

Supplementary Data 5. Whole-Cell Proteome in SU-DHL-5 SENP6 and EV DLBCL cell lines. Samples were grouped into triplicates and a Student's t test was performed with a randomization of 500 and a s0 factor of 0.1.

Supplementary Data 6. Genes corresponding to copy number alterations (CNAs) of Senp6-sgRNA lymphomas analyzed by low coverage WGS.
