# Supplementary material for: Genetic alterations of the SUMO isopeptidase SENP6 drive lymphomagenesis and genetic instability in diffuse large B-cell lymphoma
Source: Nat Commun. 2022 Jan 12;13:281. doi: 10.1038/s41467-021-27704-8 (PMC8755833; doi:10.1038/s41467-021-27704-8)
Supplement: Supplementary file 9 — Reporting summary [file 41467_2021_27704_MOESM9_ESM.pdf]

nature research

Corresponding author(s): Ulrich Keller, Stefan Müller

Last updated by author(s): 29.11.2021

Reporting Summary

Nature Research wishes to improve the reproducibility of the work that we publish. This form provides structure for consistency and transparency in reporting. For further information on Nature Research policies, see our Editorial Policies and the Editorial Policy Checklist.

Statistics

For all statistical analyses, confirm that the following items are present in the figure legend, table legend, main text, or Methods section.

n/a	Confirmed	
☐	☒	The exact sample size (*n*) for each experimental group/condition, given as a discrete number and unit of measurement
☐	☒	A statement on whether measurements were taken from distinct samples or whether the same sample was measured repeatedly
☐	☒	The statistical test(s) used AND whether they are one- or two-sided<br>*Only common tests should be described solely by name; describe more complex techniques in the Methods section.*
☐	☒	A description of all covariates tested
☐	☒	A description of any assumptions or corrections, such as tests of normality and adjustment for multiple comparisons
☐	☒	A full description of the statistical parameters including central tendency (e.g. means) or other basic estimates (e.g. regression coefficient) AND variation (e.g. standard deviation) or associated estimates of uncertainty (e.g. confidence intervals)
☐	☒	For null hypothesis testing, the test statistic (e.g. *F*, *t*, *r*) with confidence intervals, effect sizes, degrees of freedom and *P* value noted<br>*Give P values as exact values whenever suitable.*
☒	☐	For Bayesian analysis, information on the choice of priors and Markov chain Monte Carlo settings
☒	☐	For hierarchical and complex designs, identification of the appropriate level for tests and full reporting of outcomes
☐	☒	Estimates of effect sizes (e.g. Cohen's *d*, Pearson's *r*), indicating how they were calculated

Our web collection on statistics for biologists contains articles on many of the points above.

Software and code

Policy information about availability of computer code

Data collection	*MiSeq Control Software V2.6.2<br>*MiSeq RTA V.1.18.54<br>*Illumina CASAVA software within BaseSpace suit
Data analysis	*GraphPad Prism 8.3.0<br>*DeSeq2 (Love et al., 2014)<br>*Agilent Genomic Workbench software version 7.0.4.0)<br>*CIMPL (common insertion site mapping platform) (Weber et al., 2018)<br>*Trimmomatic v0.36<br>*GCRMA R package (version 2.64.0)<br>*pheatmap R package v1.0.12<br>*limma (Ritchie et al., 2015)<br>*FASTQC v0.11.9<br>*macs v1.4<br>*bedtools v2.29.0 suit<br>*deep tools v3.3.1<br>*bwa mem (Li, 2013)<br>*ImageJ v1.53f<br>*GATK toolkit v4<br>*MaxQuant Software (version 1.6.17.0)<br>*Perseus software (version 1.6.15.0)<br>*HISAT2 v2.2.0

*fgsea R package (Korotkevich et al., 2019)
* STAR (Dobin et al. 2013)
* Salmon software (v1.3.0)
* GeneTrail 3.0
* GeneTrail2

For manuscripts utilizing custom algorithms or software that are central to the research but not yet described in published literature, software must be made available to editors and reviewers. We strongly encourage code deposition in a community repository (e.g. GitHub). See the Nature Research guidelines for submitting code & software for further information.

Data

Policy information about availability of data

All manuscripts must include a data availability statement. This statement should provide the following information, where applicable:

- Accession codes, unique identifiers, or web links for publicly available datasets
- A list of figures that have associated raw data
- A description of any restrictions on data availability

The WGS data generated in this study have been deposited at EBI European Nucleo-tide Archive under accession PRJEB44263 [https://www.ebi.ac.uk/ena/browser/view/PRJEB44263]. The transcriptome data of OCI-Ly1 control and SENP6KD cells generated in this study have been deposited in the GEO database under accession code GSE180052 [https://www-ncbi-nlm-nih-gov.ezproxy.u-pec.fr/geo/query/acc.cgi?acc=GSE180052]. The mass spectrometry proteomics data generated in this study have been deposited in the ProteomeXchange Consortium via the PRIDE partner repository62 with the dataset identifier PXD027355. [https://www.ebi.ac.uk/pride/archive/projects/PXD027355]. The ChIP and transcriptome data of SU-DHL-5 EV and SENP6 cells generated in this study have been deposited in the GEO database under accession code GSE141913 [https://www-ncbi-nlm-nih-gov.ezproxy.u-pec.fr/geo/query/acc.cgi?acc=GSE141913]. The data generated in this study are provided in the Source Data file.

Field-specific reporting

Please select the one below that is the best fit for your research. If you are not sure, read the appropriate sections before making your selection.

[x] Life sciences   [ ] Behavioural & social sciences   [ ] Ecological, evolutionary & environmental sciences

For a reference copy of the document with all sections, see nature.com/documents/nr-reporting-summary-flat.pdf

Life sciences study design

All studies must disclose on these points even when the disclosure is negative.

Sample size	Sample size was determined for every set of experiments to reveal reproducibility and/or statistical significance of the data. Statistical determination of sample size was not performed.
Data exclusions	No data were excluded.
Replication	For in vivo validation of SENP6 as tumor suppressor on a Eμ-myc background, biological replicates were used (Senp6-sgRNA: n=5; control-sgRNA: n=15). For testing SENP6 loss without Eμ-myc, biological replicates were used (Senp6-sgRNA: n=6; control sg-RNA: n=5). For in vivo xenograft studies, biological replicates were used (SU-DHL-6 control: vehicle (n=5) and olaparib (n=6). SU-DHL-6 SENP6KD: vehicle (n=5) and olaparib (n=7)).<br>Cell culture based experiments have been repeated at least three times in independent experiments. The exact number of repetitions and similarity of results is indicated in the figure legends. qPCRs were performed in triplicates. Generally, all experiments have been independently repeated at least three times.
Randomization	Mice were allocated in cohorts based on genotype. No randomization was performed. For all experiments other than mouse experiments, samples were allocated into experimental groups in a random fashion.
Blinding	Investigators performing necropsies and histopathological analyses were blinded to the genotype. For all biochemical and cell biological experiments no blinding was performed due to the limited availability of persons performing the experiments.

Reporting for specific materials, systems and methods

We require information from authors about some types of materials, experimental systems and methods used in many studies. Here, indicate whether each material, system or method listed is relevant to your study. If you are not sure if a list item applies to your research, read the appropriate section before selecting a response.

Materials & experimental systems

n/a	Involved in the study
☐	☒ Antibodies
☐	☒ Eukaryotic cell lines
☒	☐ Palaeontology and archaeology
☐	☒ Animals and other organisms
☐	☒ Human research participants
☒	☐ Clinical data
☒	☐ Dual use research of concern

Methods

n/a	Involved in the study
☐	☒ ChIP-seq
☐	☒ Flow cytometry
☒	☐ MRI-based neuroimaging

Antibodies

Antibodies used

For western blotting: SUMO1 (1:1000, rabbit, Cell Signaling #4930), SUMO2/3 (1:1000, rabbit, Cell Signaling #4971), c-MYC (sc-764) (1:500, rabbit, Cell Signaling #9402), β-Actin (1:5000, mouse, Sigma-Aldrich A5316), RAD21 (1:1000, mouse, Santa Cruz sc-271601), STAG2 (1:1000, mouse, Santa Cruz sc-81852), p-CHK1 (1:1000, rabbit, Cell Signaling #2348), CHK1 (1:1000, mouse, Santa Cruz sc-56291), yH2AX (1:2000, rabbit, Abcam ab11174), CDC5L (1:1000, rabbit, Atlas HPA011361), PRP19 (1:1000, mouse, Santa Cruz sc-514338) ,Histone-H3 (1:2000, rabbit, Cell Signaling #4499), SENP6 (1:500, rabbit, Sigma-Aldrich HPA024376), SENP7 (1:500, rabbit, Abcam ab58422), β-Tubulin (0.4ug/ml, mouse, DSHB #E7), mouse IgG HRP (1:10000, sheep, GE Healthcare #NA931V), rabbit IgG HRP (1:10000, donkey, GE Healthcare #NA934V), RNF4 (1:2000, rabbit, provided by A. Vertegaal), Vinculin (1:1000, rabbit, Cell Signaling #13901).

For immunohistochemistry: SENP6 (1:100, rabbit, ER1 20 min pre-treatment, Sigma-Aldrich HPA024376), SENP7 (1:100, rabbit, ER1 30 min pre-treatment, Sigma-Aldrich HPA027259), IgM (1:75, rat, ER2 30 min pre-treatment, BD Biosciences #553435),B220 (1:50, ER1 20 min pre-treatment rat, BD Biosciences #550286).

For flow cytometry: CD45 (30-F11, 1:50, rat, eBioscience #53-0451-82), B220 (PE-Cyanine7, RA3-6B2, 1:50, rat, eBioscience #25-0452-82), IgM (PE, eB121-15F), 1:50, rat, eBiosci-ence #12-5890-82), AnnexinV (APC, 1:25, BioLegend #640919)

For ChiP: SUMO1 (15 µg, rabbit, Abcam ab32058), SUMO2/3 (15 µg, mouse, Abcam ab81371), MYC (15 µg, rabbit , Abcam ab32072) RAD21 (15 µg, rabbit, Bethyl Laboratories A300-080A).

For fluorescence microscopy: phospho-Histone H2A.X (Ser139) (2 µg/ml, mouse, Millipore #05-636), anti-Mouse IgG (H+L) Superclonal™ Secondary Antibody (Alexa Fluor 488, 1µg/ml, goat, Thermo Fisher #A28175).

Validation

All antibodies were validated by western blotting. The SENP6 antibody was validated by western blotting and by the use of a siRNA and an overexpression construct.

Eukaryotic cell lines

Policy information about cell lines

Cell line source(s)

DLBCL cell lines: SU-DHL-5, SU-DHL-6, Oci-Ly19, Oci-Ly1, HEK293T, NIH-3T3: these cell lines were purchased from DSMZ
U-2-OS tetON MYC: Walz et al. Nature 2013
Phoenix Eco (Citation: RRID:CVCL_H717): Gift

Authentication

Cell lines were authenticated by STR/DNA profiling by a company.

Mycoplasma contamination

All cell lines were tested negatively for mycoplasma contamination.

Commonly misidentified lines
(See ICLAC register)

No commonly misidentified cell lines were used.

Animals and other organisms

Policy information about studies involving animals; ARRIVE guidelines recommended for reporting animal research

Laboratory animals	Exclusively mice were used in the study.<br><br>*Eμ-myc: Adams et al. 1985: age for breeding between 8 and 20 weeks, male and female at equal ratios<br>*ATP2-H32: Rad et al. Science 2010: age for breeding between 8 and 20 weeks, male and female at equal ratios<br>*Rosa26PB: Rad et al. Science 2010: age for breeding between 8 and 20 weeks, male and female at equal ratios<br>*Rosa26Cas9 (Gt(ROSA)26Sortm1(Cas9)Rad): Weber et al. Nature Communications 2019: age for breeding between 8 and 20 weeks, male and female at equal ratios<br>*C57BL/6: Charles River: for transplantation 6-8 weeks at beginning of the experiment, female<br>*NOD.CB17-Prkdcscid/NCrCrl: Charles River: for xenograft experiments 6-8 weeks at beginning of the experiment, female<br><br>Mouse keeping: light/dark cycle of 12h each with dusk phases. Temperature was 22°C and humidity of 55% +/- 10%.
Wild animals	The study did not involve wild animals.
Field-collected samples	The study did not involve samples collected from the field.
Ethics oversight	All animal experiments were carried out in compliance the requirements of the European guidelines for the care and use of laboratory animals and were approved by the local authorities (Regierung von Oberbayern, Munich, Germany). Protocol numbers: 55.2-1-55-2532-76-15; ROB-55.2-2532.Vet_02-17-230; 55.2-1-54-2532-77-2014

Note that full information on the approval of the study protocol must also be provided in the manuscript.

Human research participants

Policy information about studies involving human research participants

Population characteristics	n/a
Recruitment	Informed consent for scientific use of biopsy material was obtained from patients.
Ethics oversight	The responsible ethics committees of the Technische Universität München approved data analysis (ethics approval 498/17s)

Note that full information on the approval of the study protocol must also be provided in the manuscript.

ChIP-seq

Data deposition

☒ Confirm that both raw and final processed data have been deposited in a public database such as GEO.

☒ Confirm that you have deposited or provided access to graph files (e.g. BED files) for the called peaks.

Data access links<br>*May remain private before publication.*	https://www.ncbi.nlm.nih.gov/geo/query/acc.cgi?acc=GSE141913
Files in database submission	GSM4216245 EV_SUMO1_ChipSeq<br>GSM4216246 SENP6_SUMO1_ChIPSeq<br>GSM4216247 EV_SUMO2_ChipSeq<br>GSM4216248 SENP6_SUMO2_ChIPSeq<br>GSM4216249 EV_Input<br>GSM4216250 SENP6_Input<br>GSM5456902 SUDLH5_EV_MYC_ChipSeq<br>GSM5456903 SUDLH5_SENP6_MYC_ChIPSeq<br>GSM5456904 SUDLH5_EV_RAD21_ChipSeq<br>GSM5456905 SUDLH5_SENP6_RAD21_ChIPSeq<br>GSM5456906 SUDLH5_Combined_Input
Genome browser session<br>(e.g. UCSC)	N/A

Methodology

Replicates	The experiment was performed with three independent repetitions per condition.
Sequencing depth	12.2 - 13.4 million reads per sample were sequenced; 10.3 million mapped reads per sample were eventually used.
Antibodies	SUMO1 (Abcam, ab32058); SUMO2/3 (Abcam, ab81371); MYC (ab32072), Abcam (ChIP); RAD21 (A300-080A), Bethyl Laboratories;

	(ChIP)
Peak calling parameters	Peak calling was carried out using macs v1.4 and peak annotation was performed with bedtools 583 v2.29.0 suit.<br>Parameters : -p 1e-6 and -keepdup 5
Data quality	Quality control of FASTQ files was performed using FASTQC.
Software	R and deeptools v1.3 (https://doi.org/10.1093/nar/gku365) was used for data analysis.

Flow Cytometry

Plots

Confirm that:

- [x] The axis labels state the marker and fluorochrome used (e.g. CD4-FITC).
- [x] The axis scales are clearly visible. Include numbers along axes only for bottom left plot of group (a 'group' is an analysis of identical markers).
- [x] All plots are contour plots with outliers or pseudocolor plots.
- [x] A numerical value for number of cells or percentage (with statistics) is provided.

Methodology

Sample preparation	Cells (primary murine lymphoma cells, HSPCs, human DLBCL cells) were washed 2x with PBS before FACS analysis. Samples were stained with antibodies in PBS+0.5%BSA for 30 minutes on ice, 1x washed with PBS and resuspended in DAPI or PI staining solution. For annexin V staining, cells were incubated with antibodies in annexin buffer containing either DAPI or PI for 15 minutes on 4°C and analysed without additional washing step.
Instrument	*CyAN (Beckmann Coulter)<br>*CytoFLEX S(Beckmann Coulter)
Software	*FlowJo (Tree Star Inc.)
Cell population abundance	HSPCs: 15-25% of cells were GFP postive prior to transplantation<br>SU-DHL-5 EV and SENP6: >90% GFP% following sort enrichment
Gating strategy	FSC/SSC gating was performed to exclude cell debris. Next, gating for either DAPI or PI negative cells was performed to distinguish viable cells from dead cells (DAPI or PI positive). For primary lymphoma cells, the viable cell population was analysed for the indicated surface antibodies to analyse the B-cell lymphoma phenotype.

- [x] Tick this box to confirm that a figure exemplifying the gating strategy is provided in the Supplementary Information.
