# Supplementary material for: Genetic alterations of the SUMO isopeptidase SENP6 drive lymphomagenesis and genetic instability in diffuse large B-cell lymphoma
Source: Nat Commun. 2022 Jan 12;13:281. doi: 10.1038/s41467-021-27704-8 (PMC8755833; doi:10.1038/s41467-021-27704-8)
Supplement: Supplementary file 10 — Source Data [file 41467_2021_27704_MOESM10_ESM.zip › Source Data II.pdf]

Figure 3b

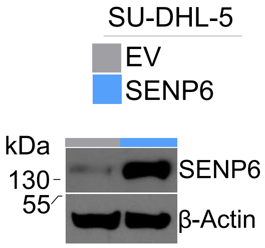

SU-DHL-5
EV
SENP6
kDa
130
55
SENP6
β-Actin

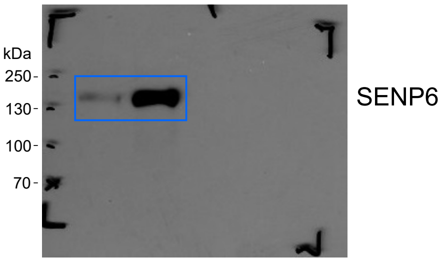

kDa
250
130
100
70
SENP6

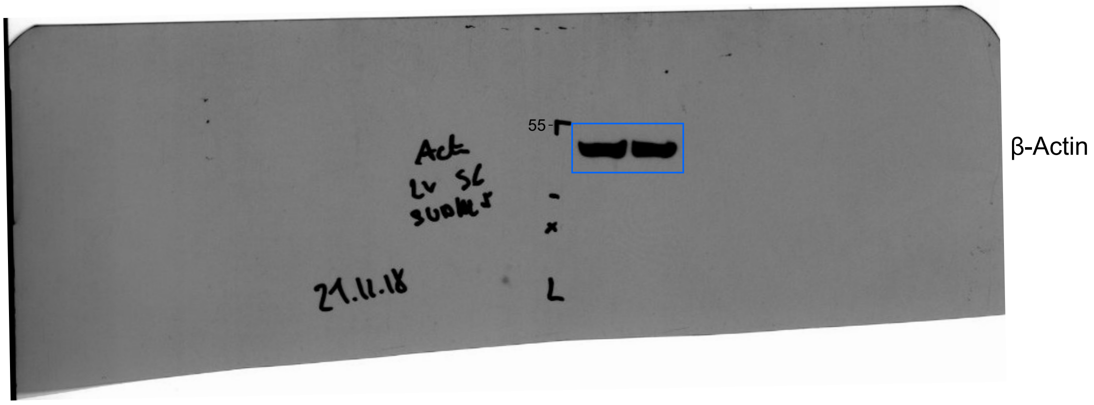

55
β-Actin
Act
EV S6
SUDHL5
21.11.18

Figure 4a

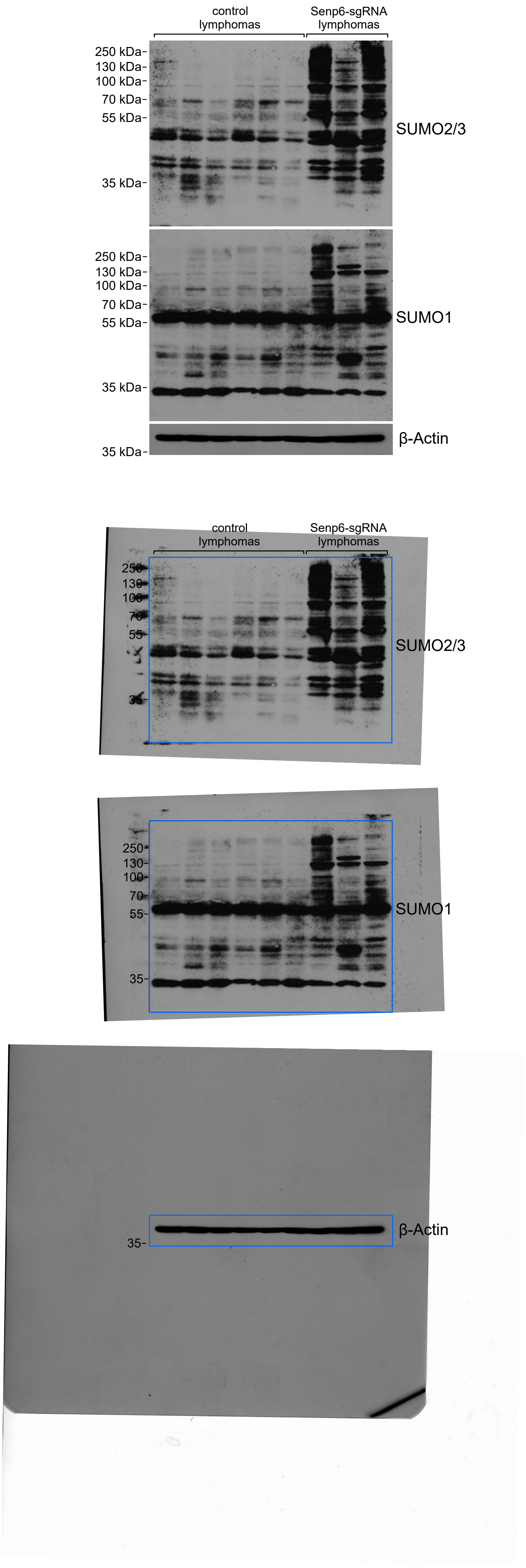
control lymphomas
Senp6-sgRNA lymphomas
250 kDa
130 kDa
100 kDa
70 kDa
55 kDa
35 kDa
SUMO2/3
SUMO1
β-Actin
250
130
100
70
55
35

Figure 4b

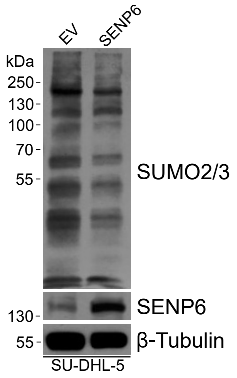
EV
SENP6
kDa
250
130
100
70
55
SUMO2/3
SENP6
130
55
β-Tubulin
SU-DHL-5

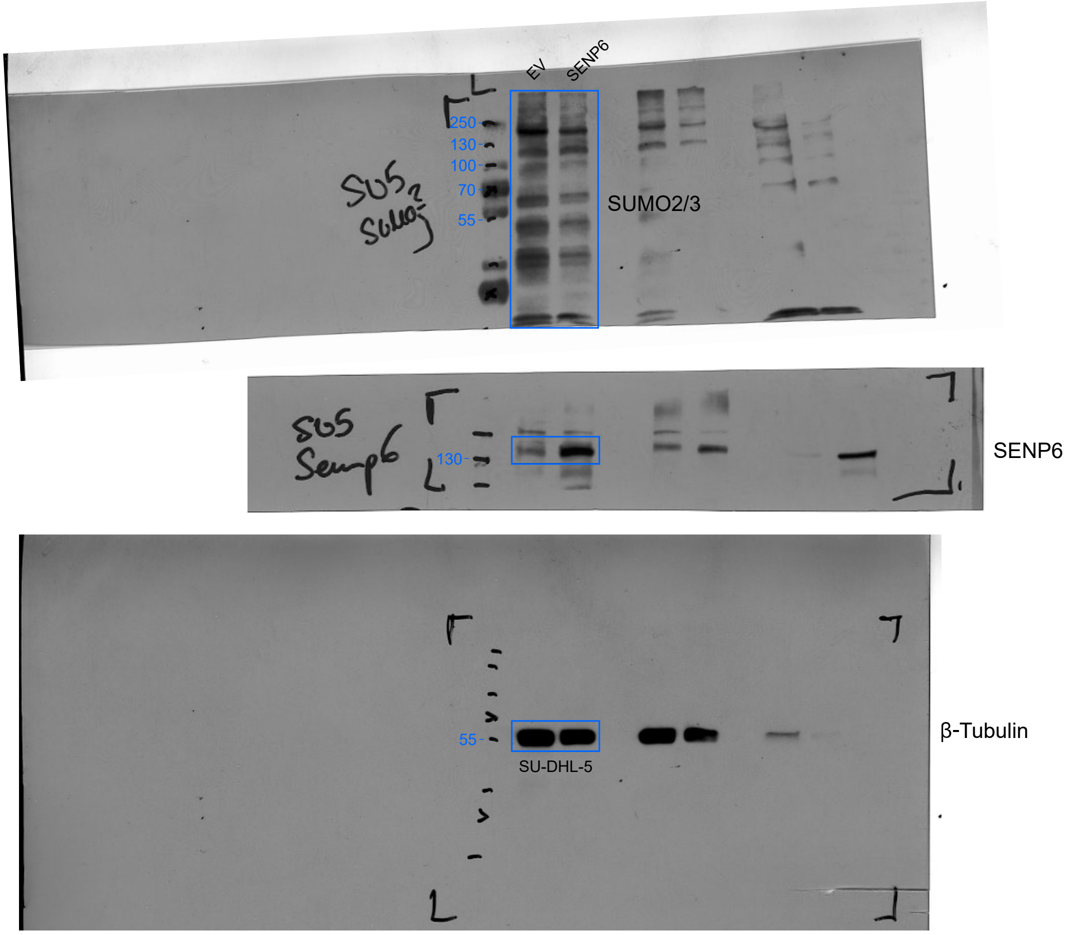
EV
SENP6
250
130
100
70
55
SUMO2/3
130
SENP6
55
β-Tubulin
SU-DHL-5

Figure 4c

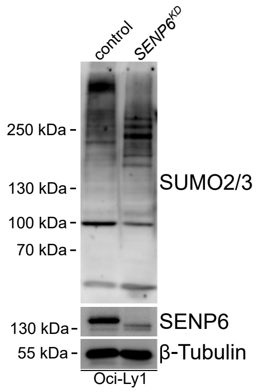
control
SENP6KD
250 kDa
130 kDa
100 kDa
70 kDa
SUMO2/3
130 kDa
SENP6
55 kDa
β-Tubulin
Oci-Ly1

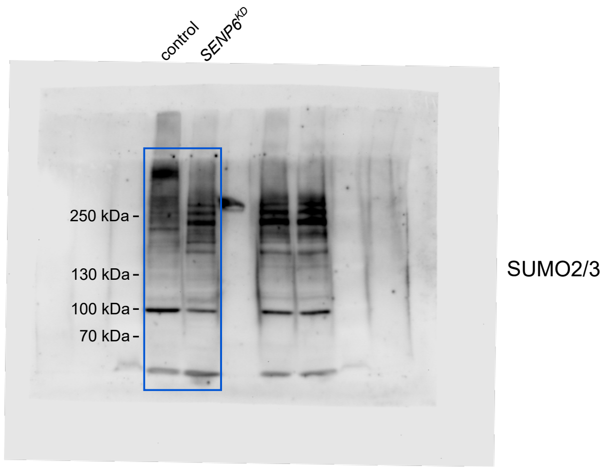
control
SENP6KD
250 kDa
130 kDa
100 kDa
70 kDa
SUMO2/3

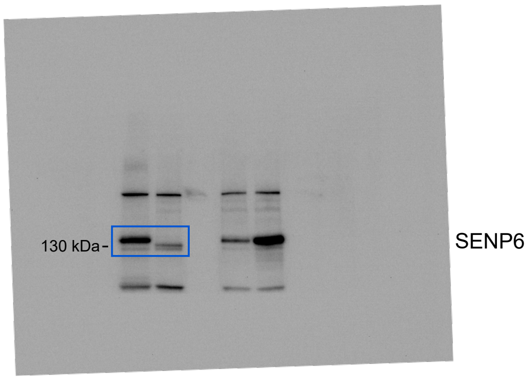
130 kDa
SENP6

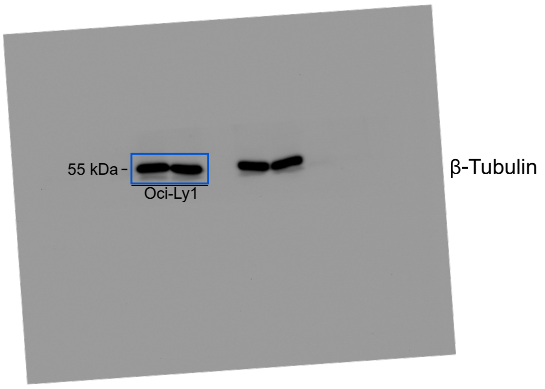
55 kDa
Oci-Ly1
β-Tubulin

Figure 4f

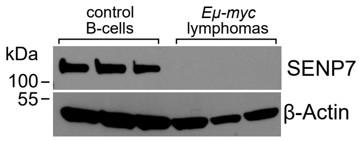
control
B-cells
Eμ-myc
lymphomas
kDa
100
55
SENP7
β-Actin

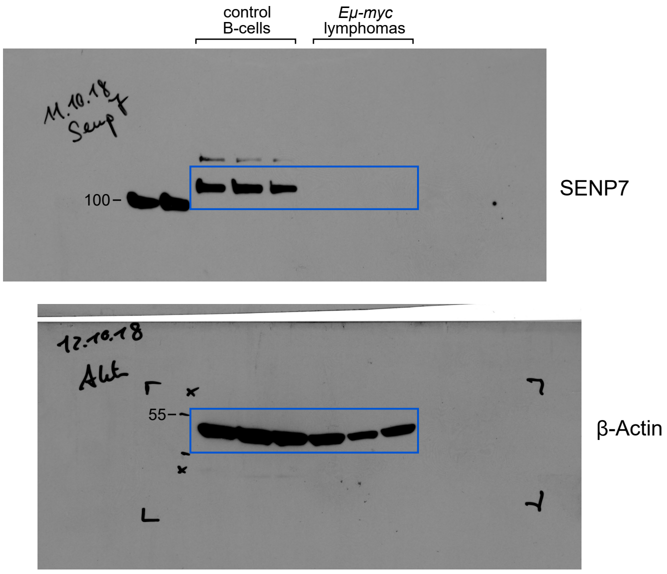
control
B-cells
Eμ-myc
lymphomas
100
SENP7
55
β-Actin

Figure 5a

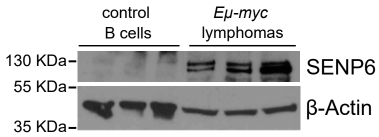
control
B cells
Eμ-myc
lymphomas
130 KDa
55 KDa
35 KDa
SENP6
β-Actin

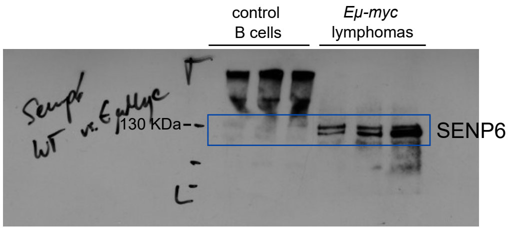
control
B cells
Eμ-myc
lymphomas
130 KDa
SENP6

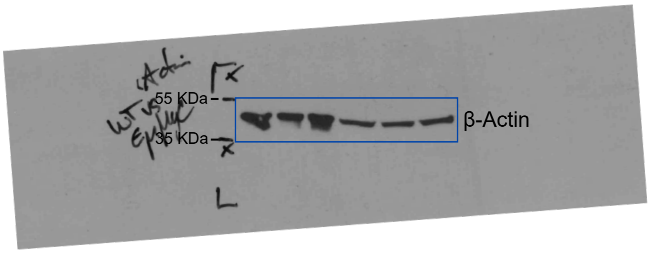
55 KDa
35 KDa
β-Actin

Figure 5b

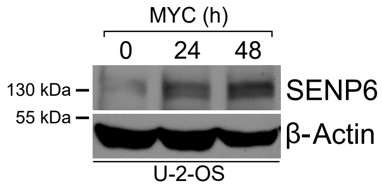

MYC (h)
0
24
48
130 kDa
55 kDa
SENP6
β-Actin
U-2-OS

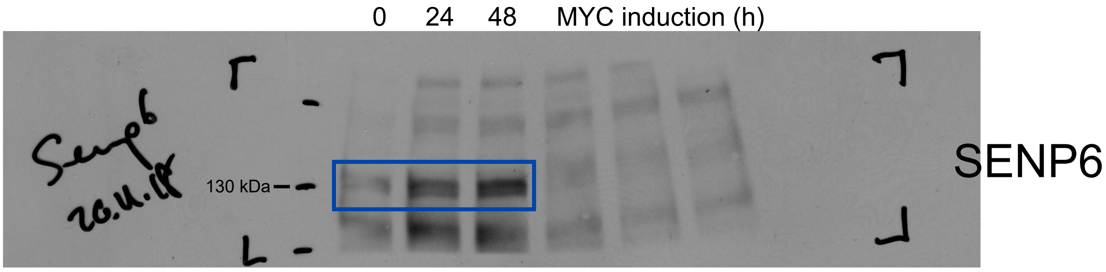

0
24
48
MYC induction (h)
130 kDa
SENP6

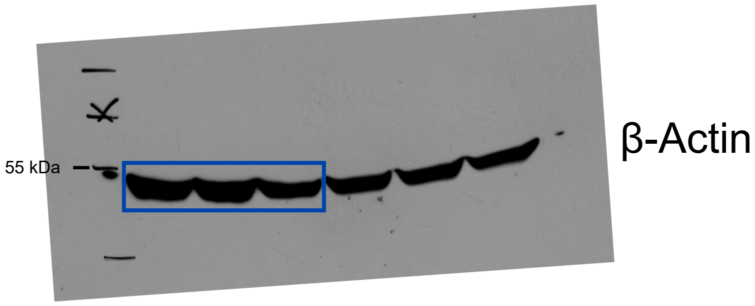

55 kDa
β-Actin

Figure 5d

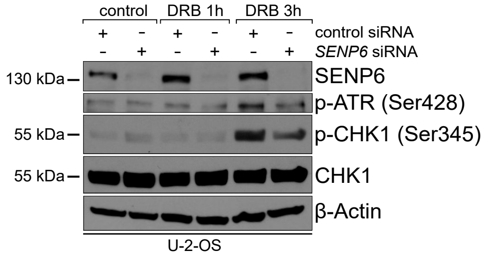

control
DRB 1h
DRB 3h
control siRNA
SENP6 siRNA
130 kDa
SENP6
p-ATR (Ser428)
55 kDa
p-CHK1 (Ser345)
55 kDa
CHK1
β-Actin
U-2-OS

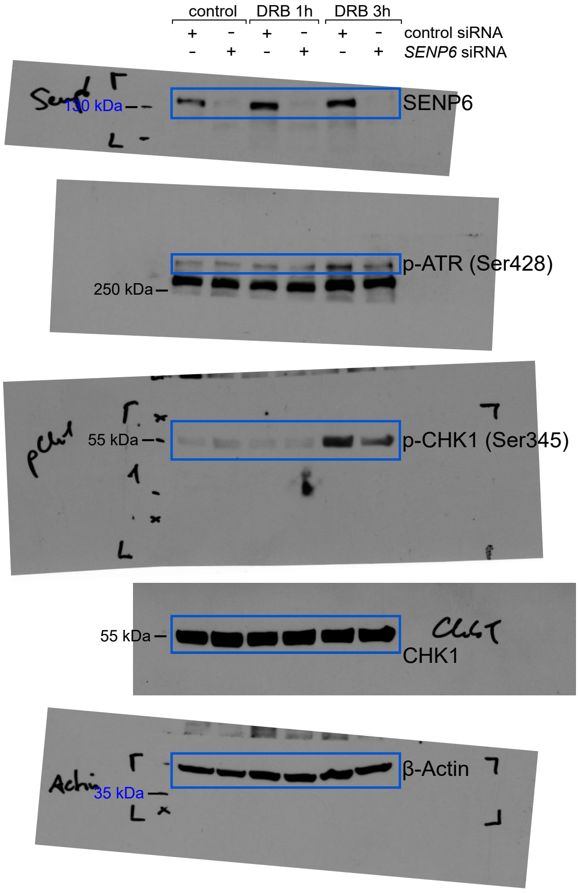

control
DRB 1h
DRB 3h
control siRNA
SENP6 siRNA
130 kDa
SENP6
p-ATR (Ser428)
250 kDa
55 kDa
p-CHK1 (Ser345)
55 kDa
CHK1
β-Actin
35 kDa

Figure 5e

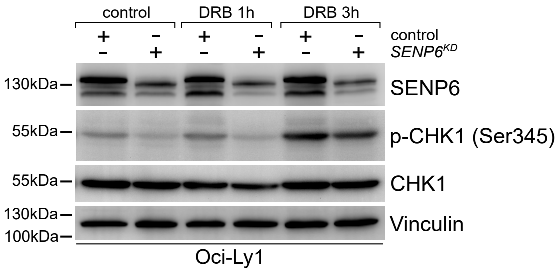
control
DRB 1h
DRB 3h
control
SENP6KD
130kDa
SENP6
55kDa
p-CHK1 (Ser345)
55kDa
CHK1
130kDa
100kDa
Vinculin
Oci-Ly1

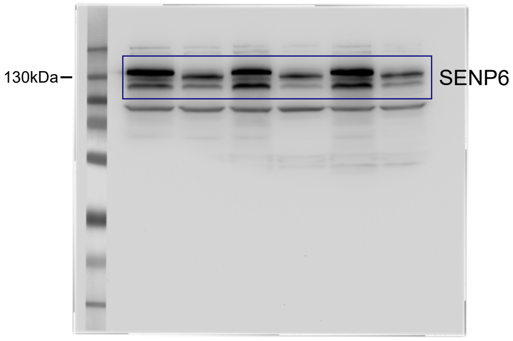
130kDa
SENP6

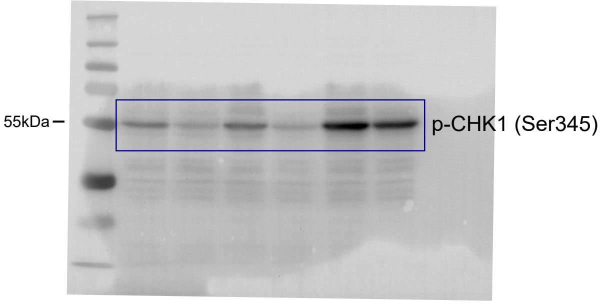
55kDa
p-CHK1 (Ser345)

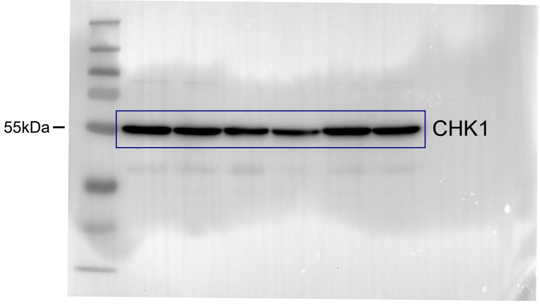
55kDa
CHK1

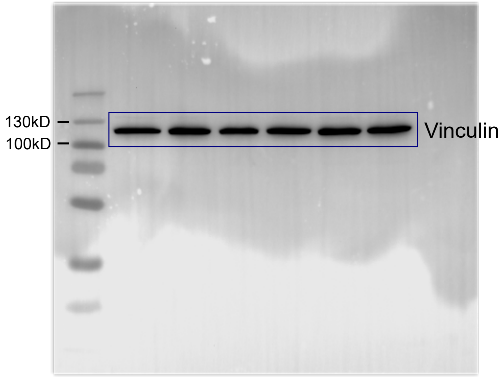
130kD
100kD
Vinculin

Figure 5f

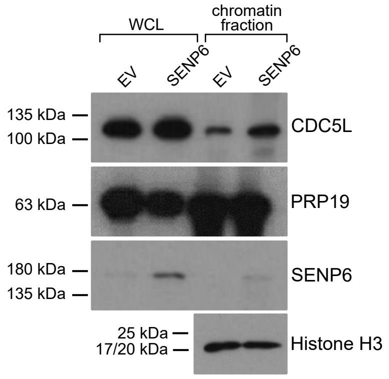
WCL
chromatin fraction
EV
SENP6
EV
SENP6
135 kDa
100 kDa
CDC5L
63 kDa
PRP19
180 kDa
135 kDa
SENP6
25 kDa
17/20 kDa
Histone H3

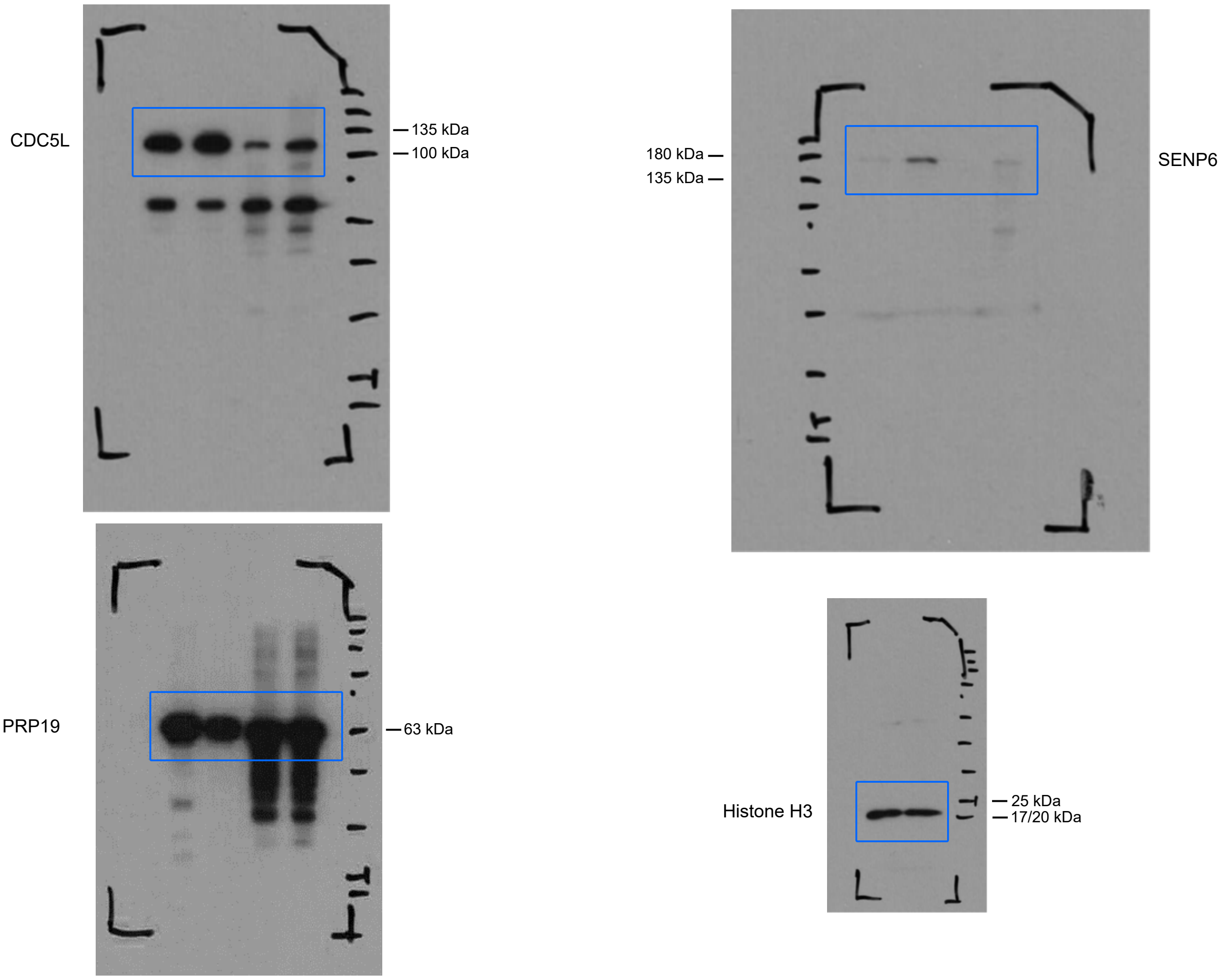
CDC5L
135 kDa
100 kDa
PRP19
63 kDa
180 kDa
135 kDa
SENP6
Histone H3
25 kDa
17/20 kDa

Figure 5g

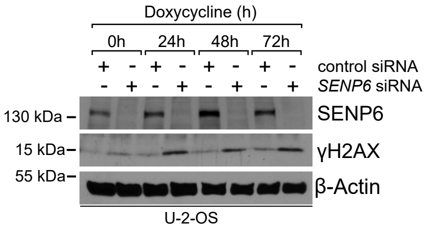
Doxycycline (h)
0h
24h
48h
72h
+ - + - + - + -
control siRNA
- + - + - + - +
SENP6 siRNA
130 kDa
SENP6
15 kDa
γH2AX
55 kDa
β-Actin
U-2-OS

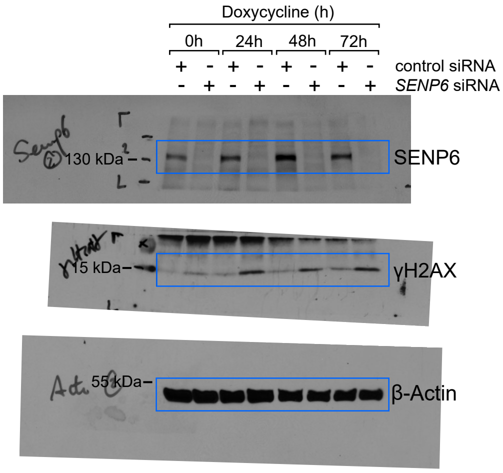
Doxycycline (h)
0h
24h
48h
72h
+ - + - + - + -
control siRNA
- + - + - + - +
SENP6 siRNA
130 kDa
SENP6
15 kDa
γH2AX
55 kDa
β-Actin

Figure 6c

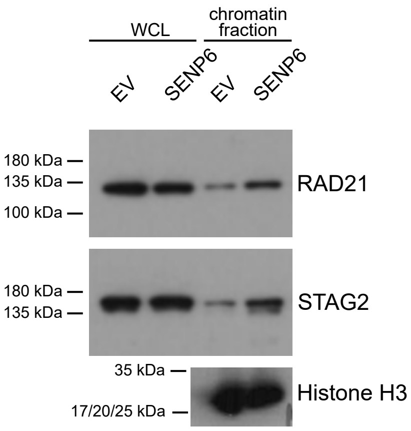
WCL
chromatin fraction
EV
SENP6
EV
SENP6
180 kDa
135 kDa
100 kDa
RAD21
180 kDa
135 kDa
STAG2
35 kDa
17/20/25 kDa
Histone H3

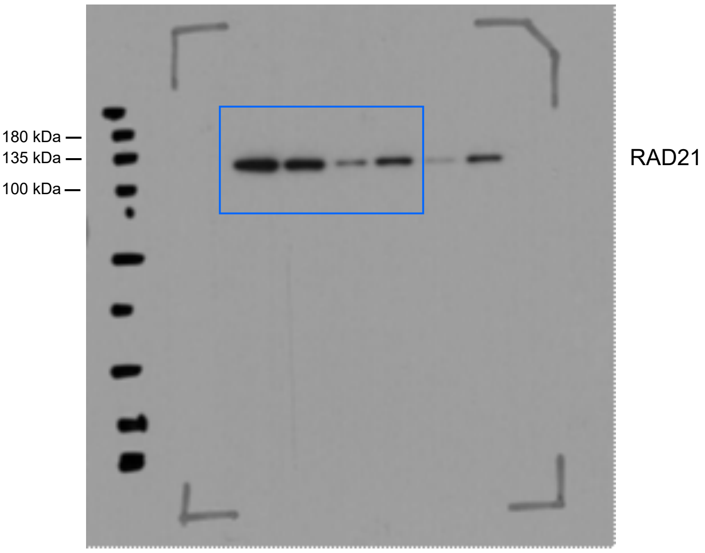
180 kDa
135 kDa
100 kDa
RAD21

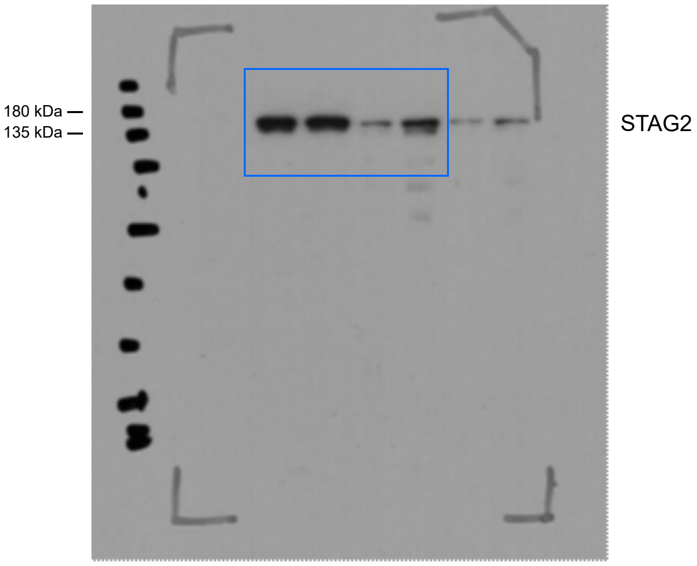
180 kDa
135 kDa
STAG2

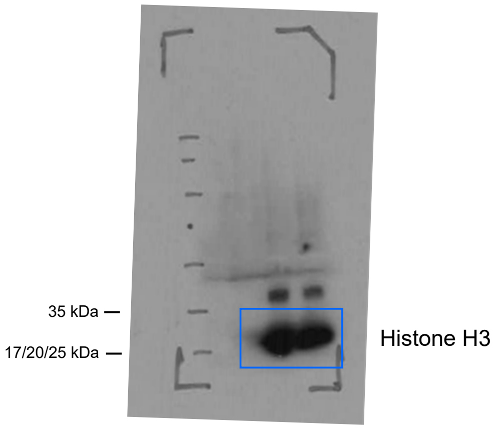
35 kDa
17/20/25 kDa
Histone H3

Figure S3g

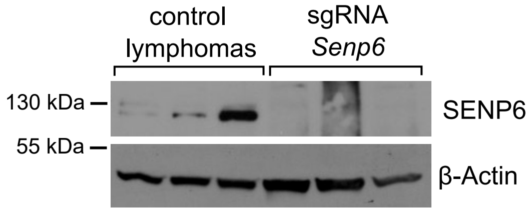
control
lymphomas
sgRNA
Senp6
130 kDa
55 kDa
SENP6
β-Actin

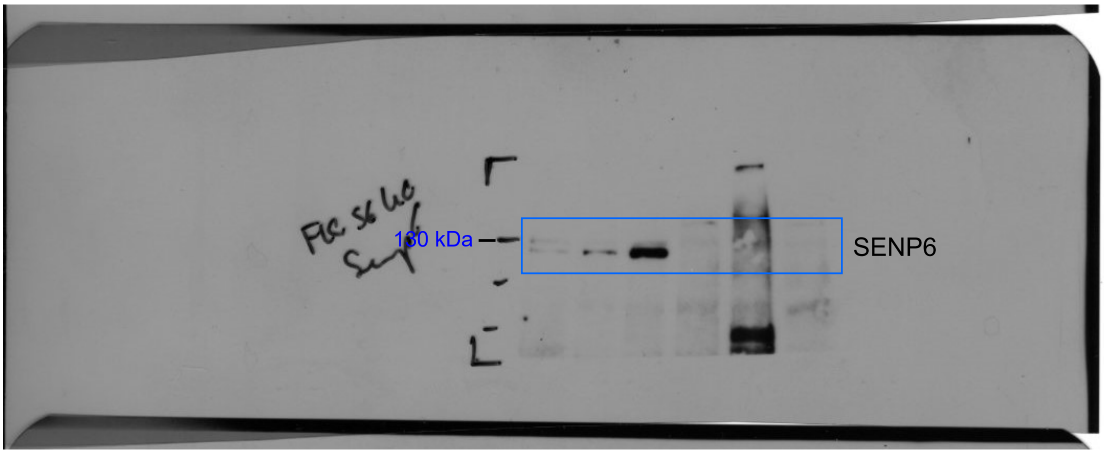
130 kDa
SENP6

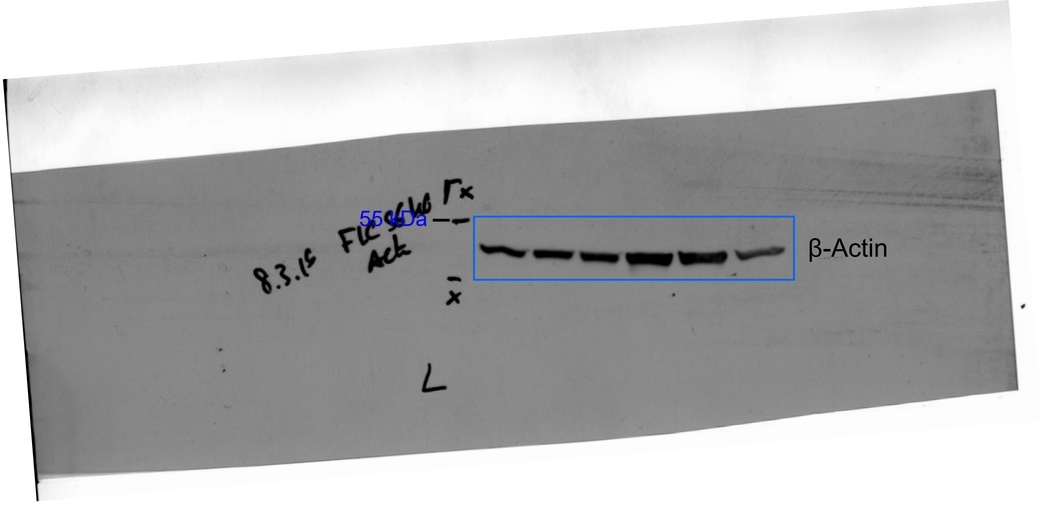
55 kDa
β-Actin

Figure S5a

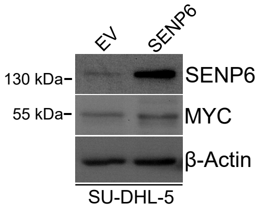
EV
SENP6
130 kDa
55 kDa
SENP6
MYC
β-Actin
SU-DHL-5

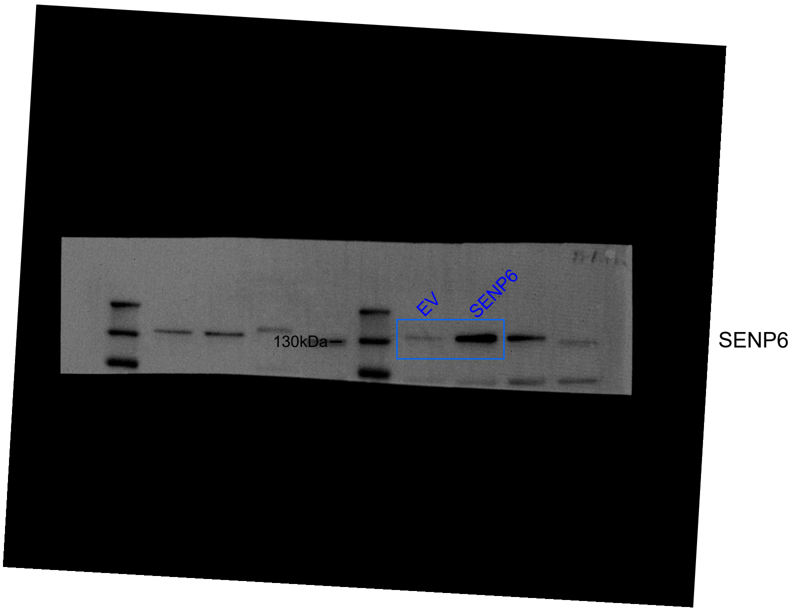
EV
SENP6
130kDa
SENP6

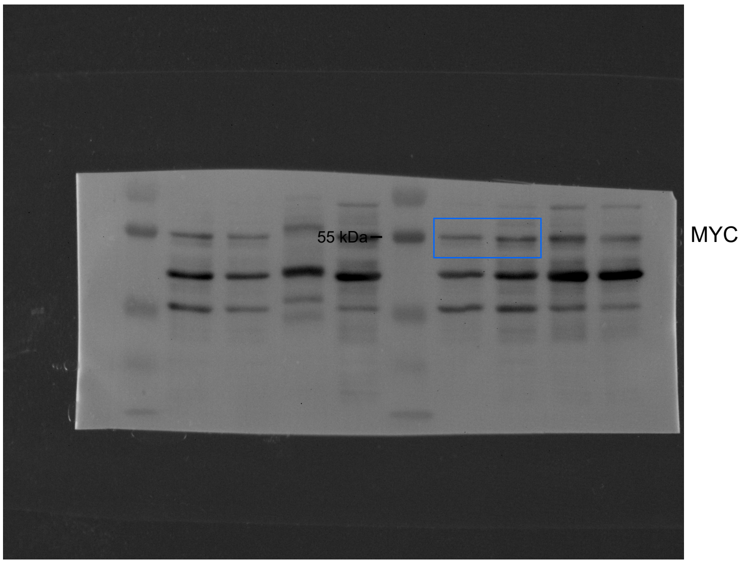
55 kDa
MYC

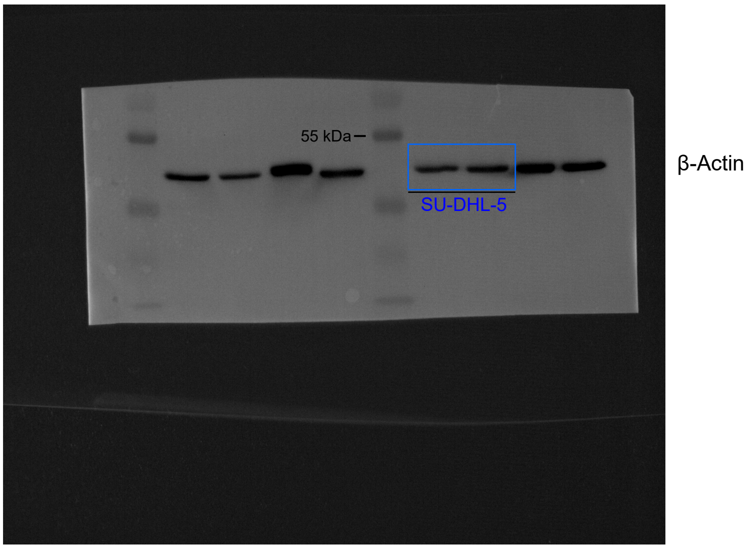
55 kDa
SU-DHL-5
β-Actin

Figure S8i

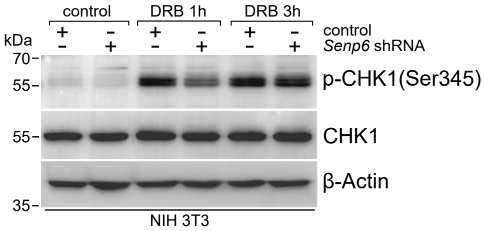

control
DRB 1h
DRB 3h
control
Senp6 shRNA
kDa
70
55
p-CHK1(Ser345)
55
CHK1
β-Actin
35
NIH 3T3

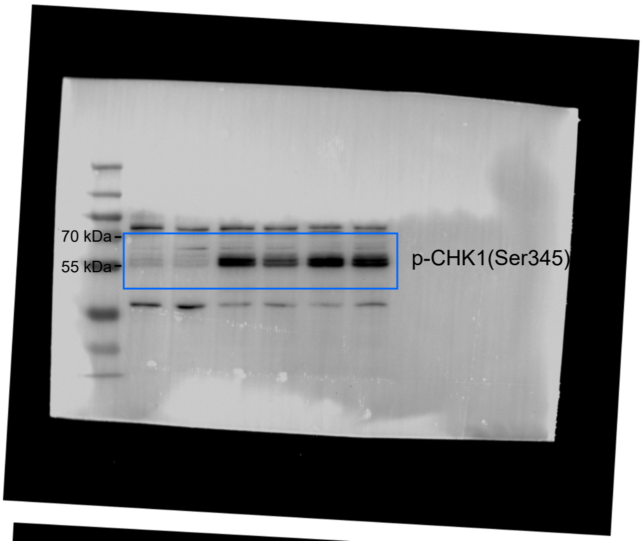

70 kDa
55 kDa
p-CHK1(Ser345)

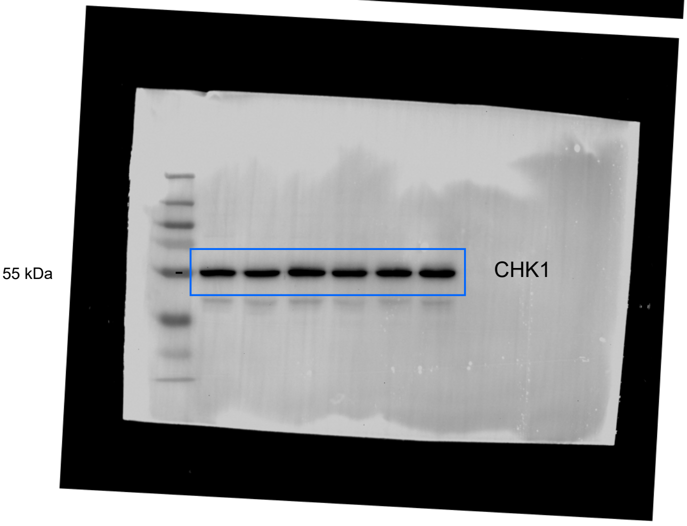

55 kDa
CHK1

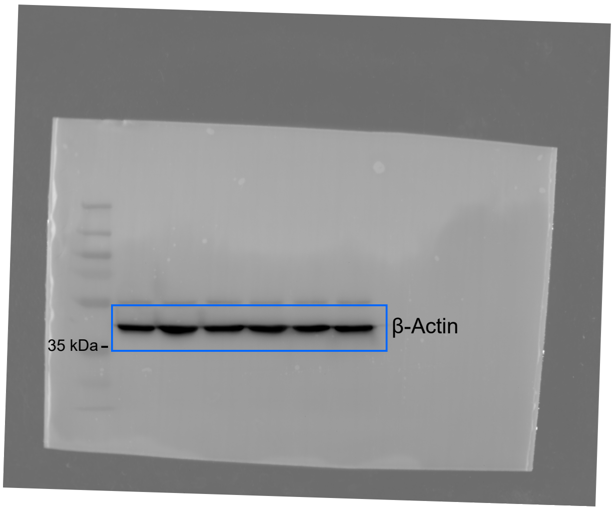

β-Actin
35 kDa

Figure S9a

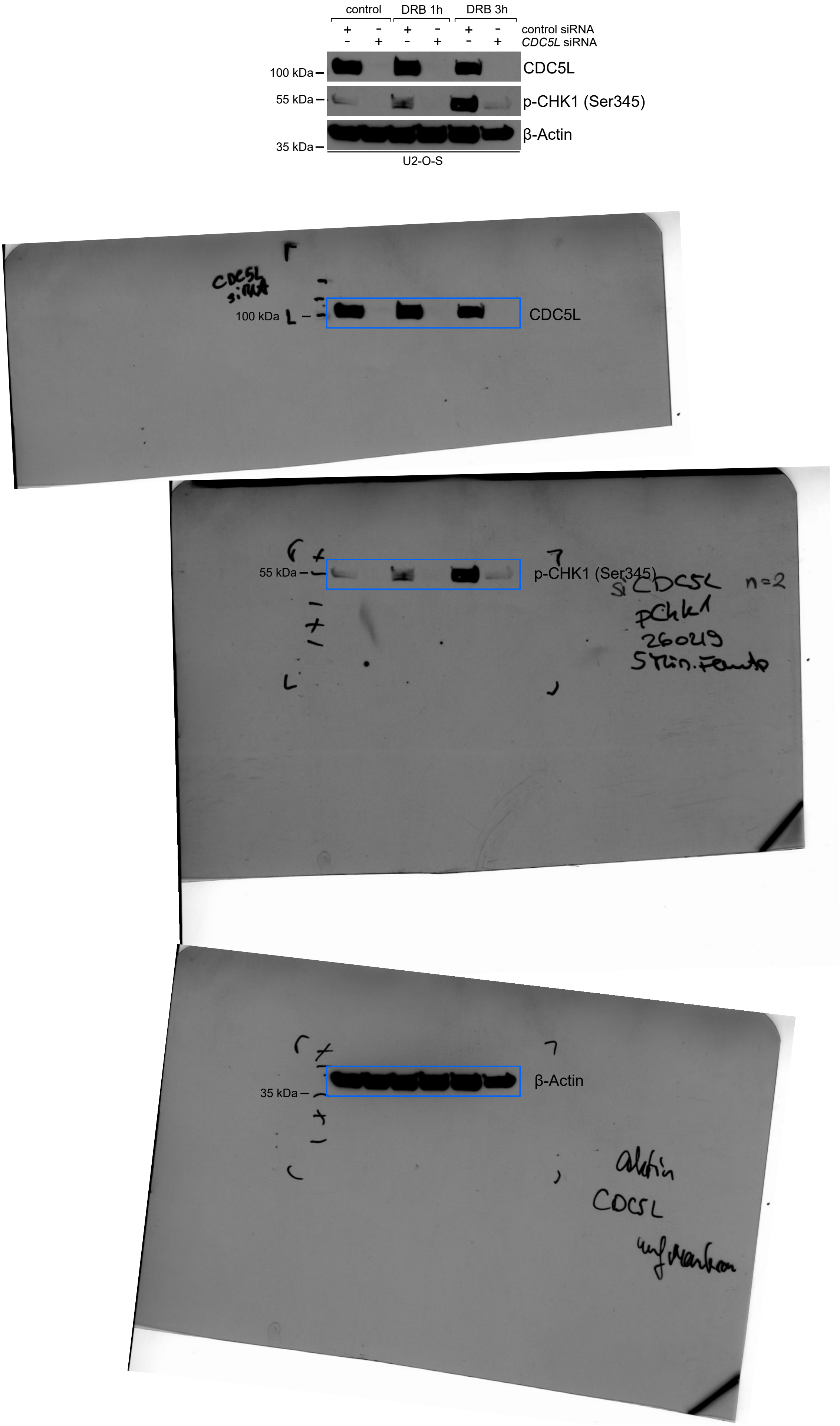
control
DRB 1h
DRB 3h
control siRNA
CDC5L siRNA
100 kDa
55 kDa
35 kDa
CDC5L
p-CHK1 (Ser345)
β-Actin
U2-O-S
CDC5L siRNA

Figure S9c

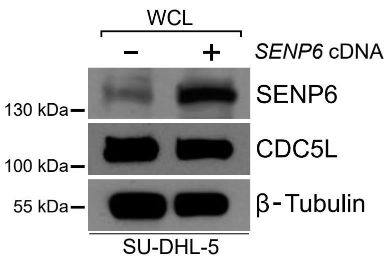

WCL
–
+
SENP6 cDNA
130 kDa
SENP6
100 kDa
CDC5L
55 kDa
β-Tubulin
SU-DHL-5

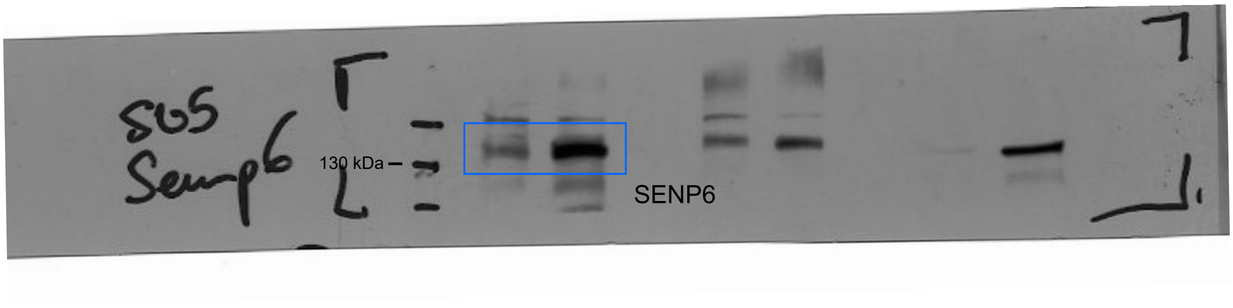

130 kDa
SENP6

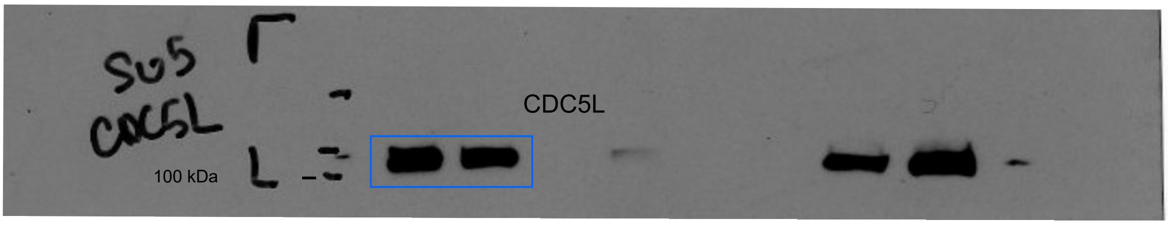

CDC5L
100 kDa

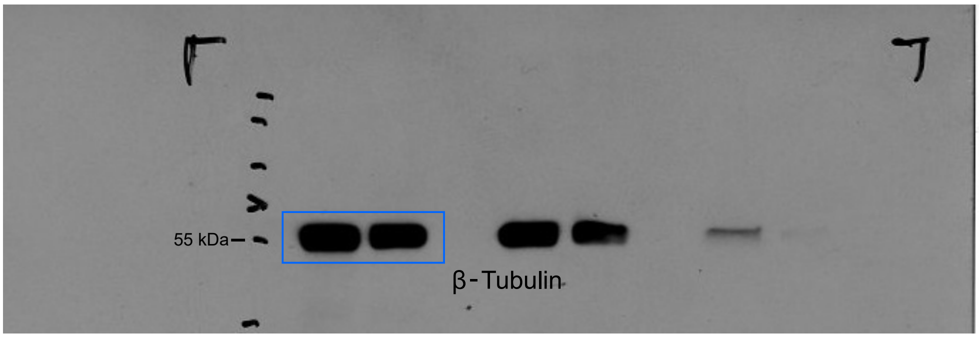

55 kDa
β-Tubulin

Figure S9d

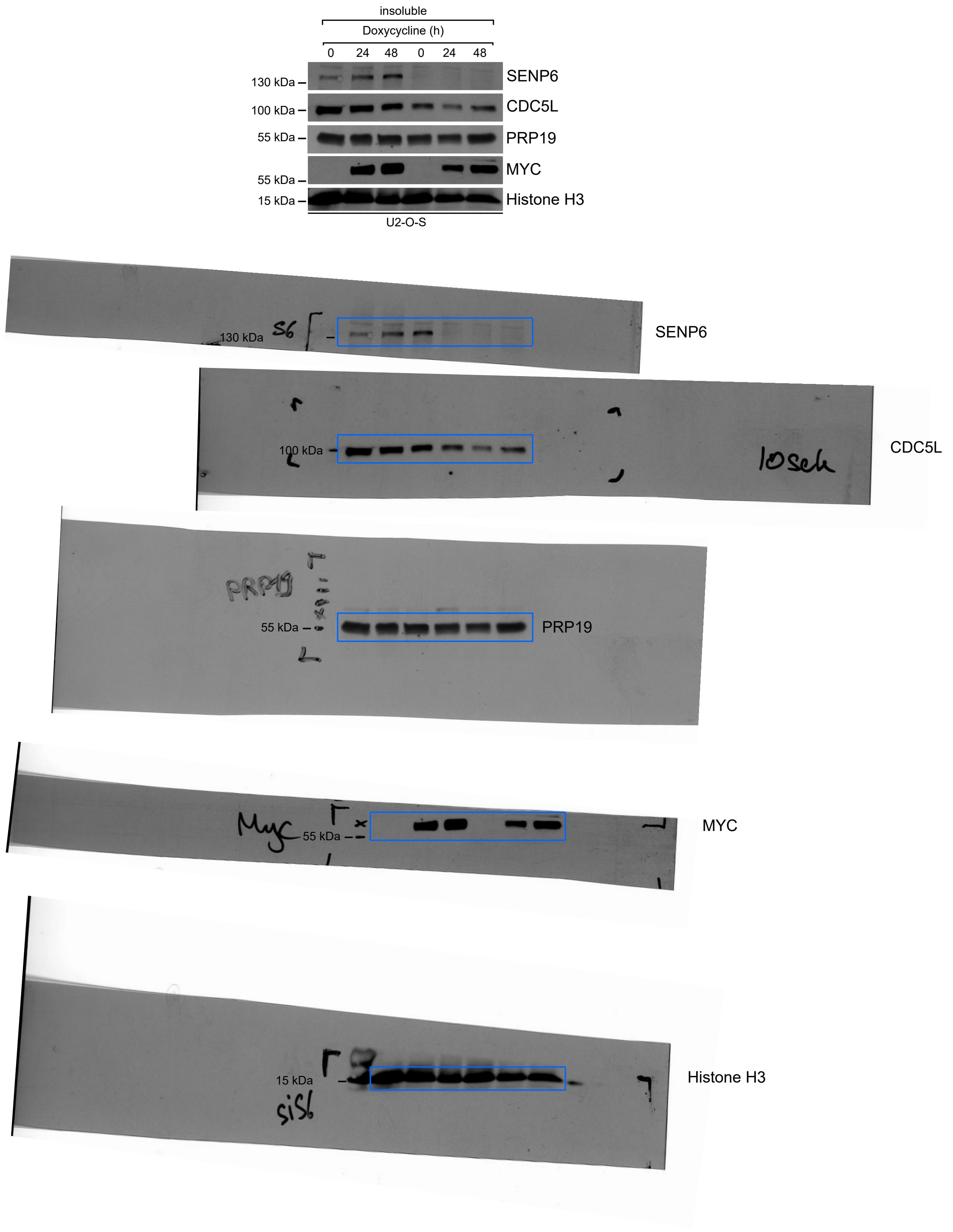

insoluble
Doxycycline (h)
0 24 48 0 24 48
130 kDa
100 kDa
55 kDa
55 kDa
15 kDa
SENP6
CDC5L
PRP19
MYC
Histone H3
U2-O-S
130 kDa
SENP6
100 kDa
CDC5L
10sek
55 kDa
PRP19
55 kDa
MYC
15 kDa
Histone H3

Figure S13a

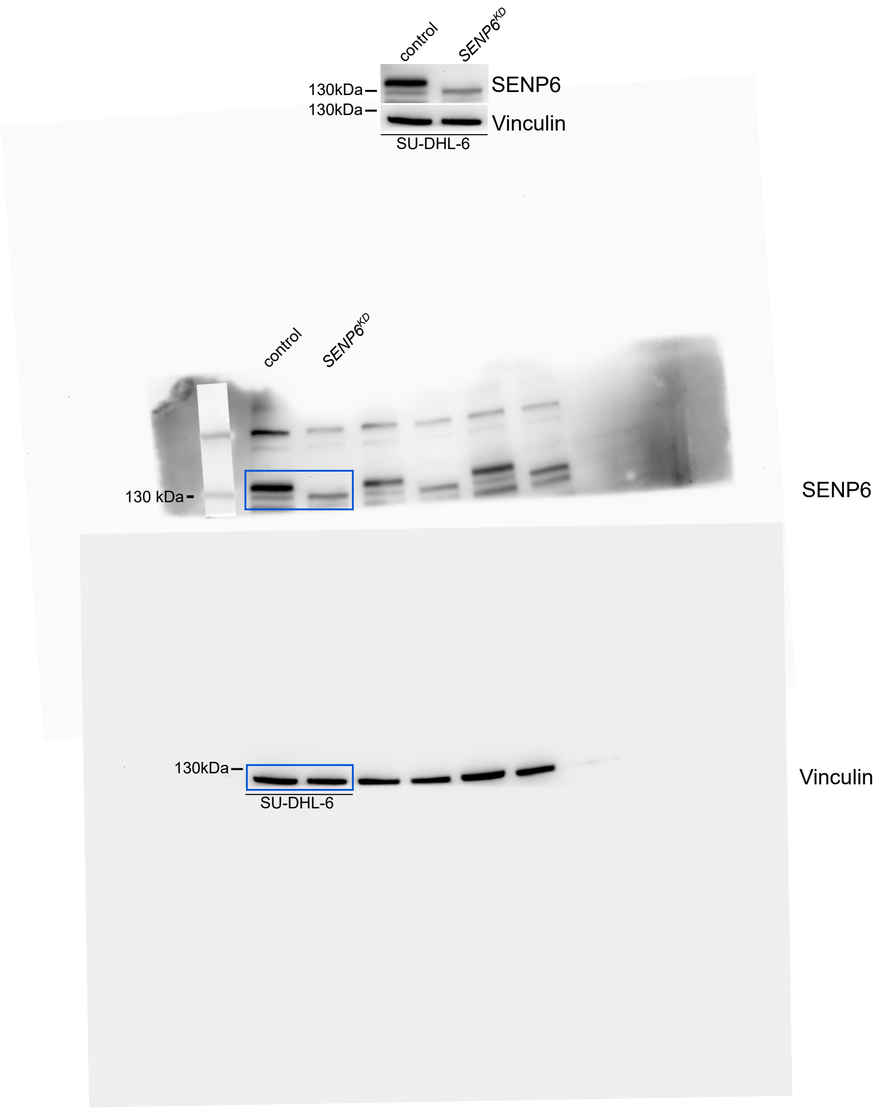

control
SENP6KD
130kDa
130kDa
SENP6
Vinculin
SU-DHL-6
control
SENP6KD
130 kDa
SENP6
130kDa
SU-DHL-6
Vinculin

Figure S14c

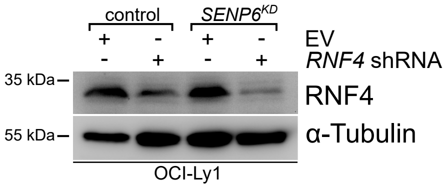

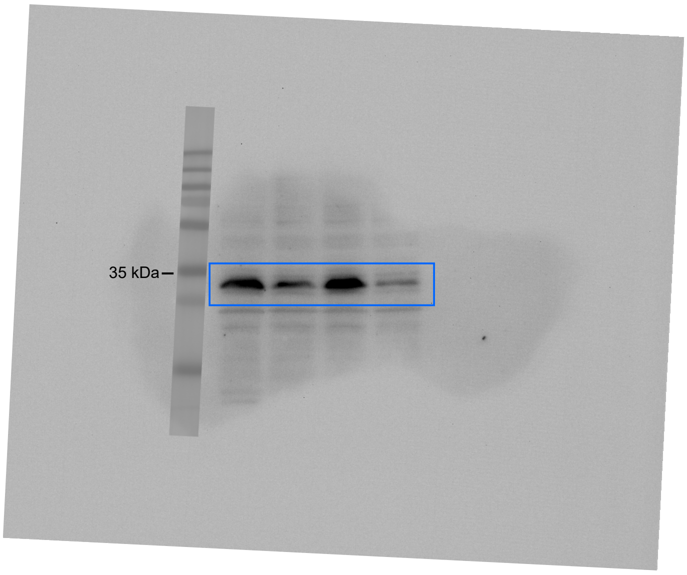

RNF4

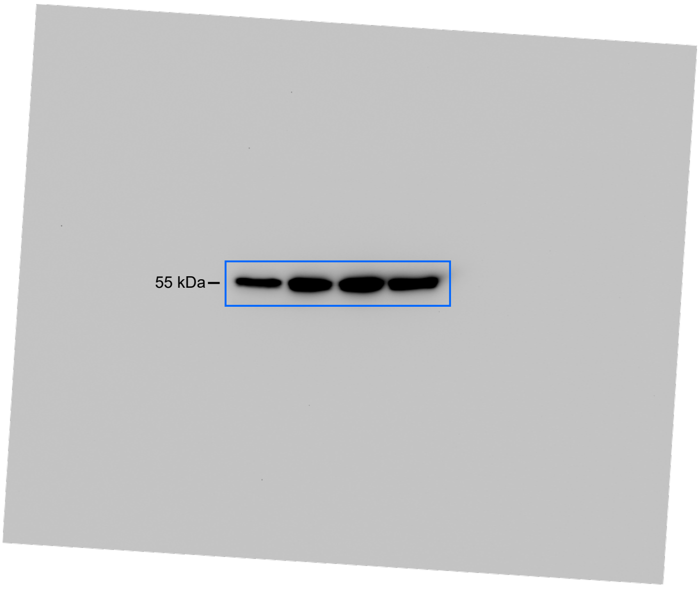

α-Tubulin
